# Supplementary material for: Deep phenotyping of post-infectious myalgic encephalomyelitis/chronic fatigue syndrome
Source: Nat Commun. 2024 Feb 21;15:907. doi: 10.1038/s41467-024-45107-3 (PMC10881493; doi:10.1038/s41467-024-45107-3)
Supplement: Supplementary file 1 — Supplementary Information [file 41467_2024_45107_MOESM1_ESM.pdf]

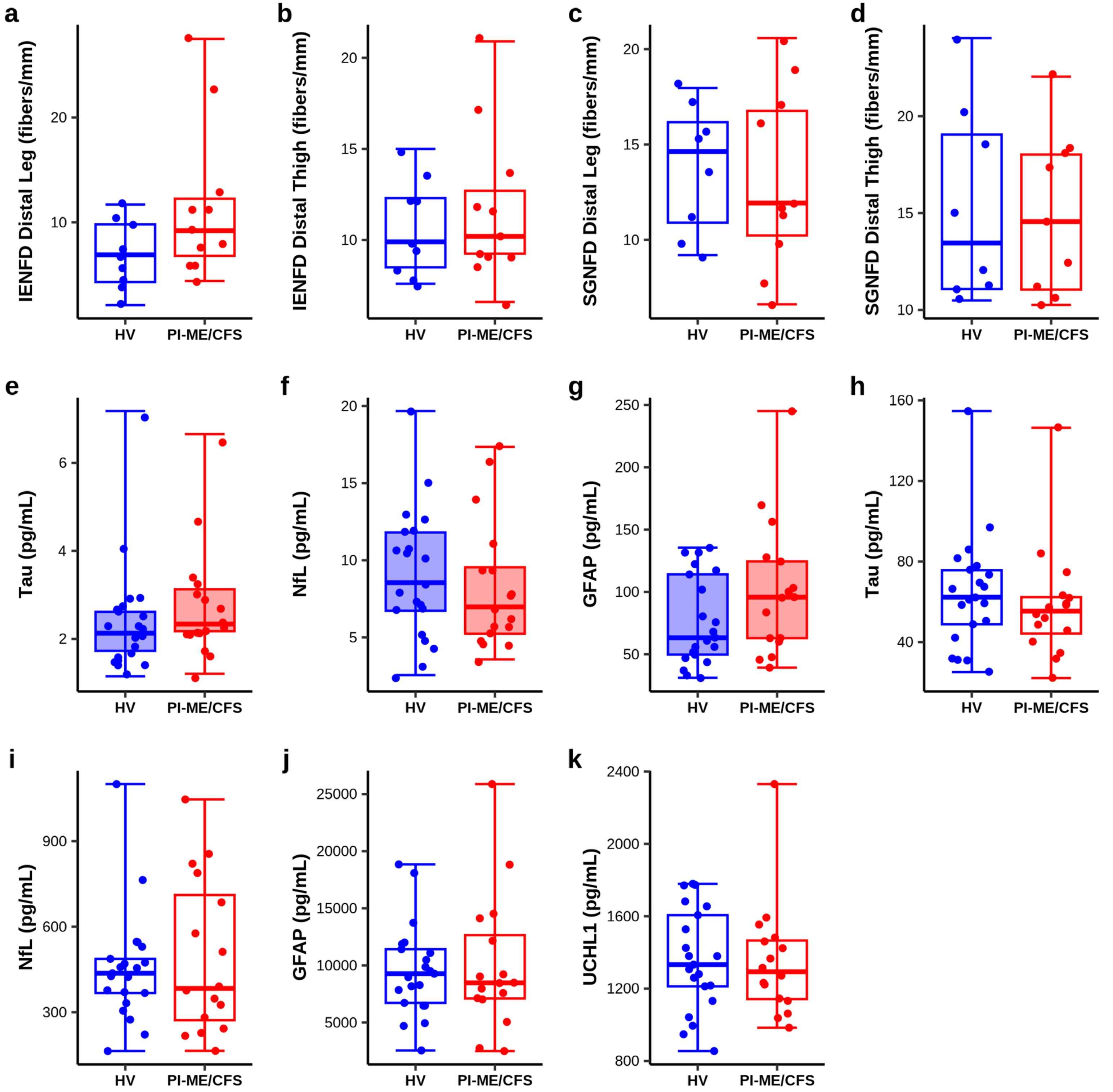

a
IENFD Distal Leg (fibers/mm)
b
IENFD Distal Thigh (fibers/mm)
c
SGNFD Distal Leg (fibers/mm)
d
SGNFD Distal Thigh (fibers/mm)
e
Tau (pg/mL)
f
NfL (pg/mL)
g
GFAP (pg/mL)
h
Tau (pg/mL)
i
NfL (pg/mL)
j
GFAP (pg/mL)
k
UCHL1 (pg/mL)
HV
PI-ME/CFS

Figure S1: Small nerve fiber density measurements and markers of neuronal injury:
A-D. Boxplots of the indicated small nerve density measurement on the y axis in HV and PI-ME/CFS groups. A-B in HVs (blue; n = 9 independent participants) and PI-ME/CFS (red; n = 11 independent participants) groups, C-D in HVs (blue; n = 8 independent participants) and PI-ME/CFS (red; n = 10 independent participants) groups. No statistical differences were noted between groups. E-G. Boxplots of the indicated neuronal injury marker measured in plasma samples. No statistical differences were noted between groups of HVs (blue; n = 21 independent participants) and PI-ME/CFS patients (red; n = 17 independent participants). H-K. Boxplots of the indicated neuronal injury marker measured in cerebrospinal fluid samples. No statistical differences were noted between groups of HVs (blue; n = 21 independent participants) and PI-ME/CFS patients (red; n = 16 independent participants). For box plots a-k boxes depict the median (horizontal line) within quartiles 1–3 (bounds of box). Whiskers extend to minimum and maximum values. Abbreviations. IENFD: intraepithelial nerve fiber density, SGNFD: sweat gland nerve fiber density, NfL: neurofilament light chain, GFAP: glial fibrillary acidic protein, UCHL1: ubiquitin carboxyl-terminal esterase L1. Source data are provided as a Source Data file.

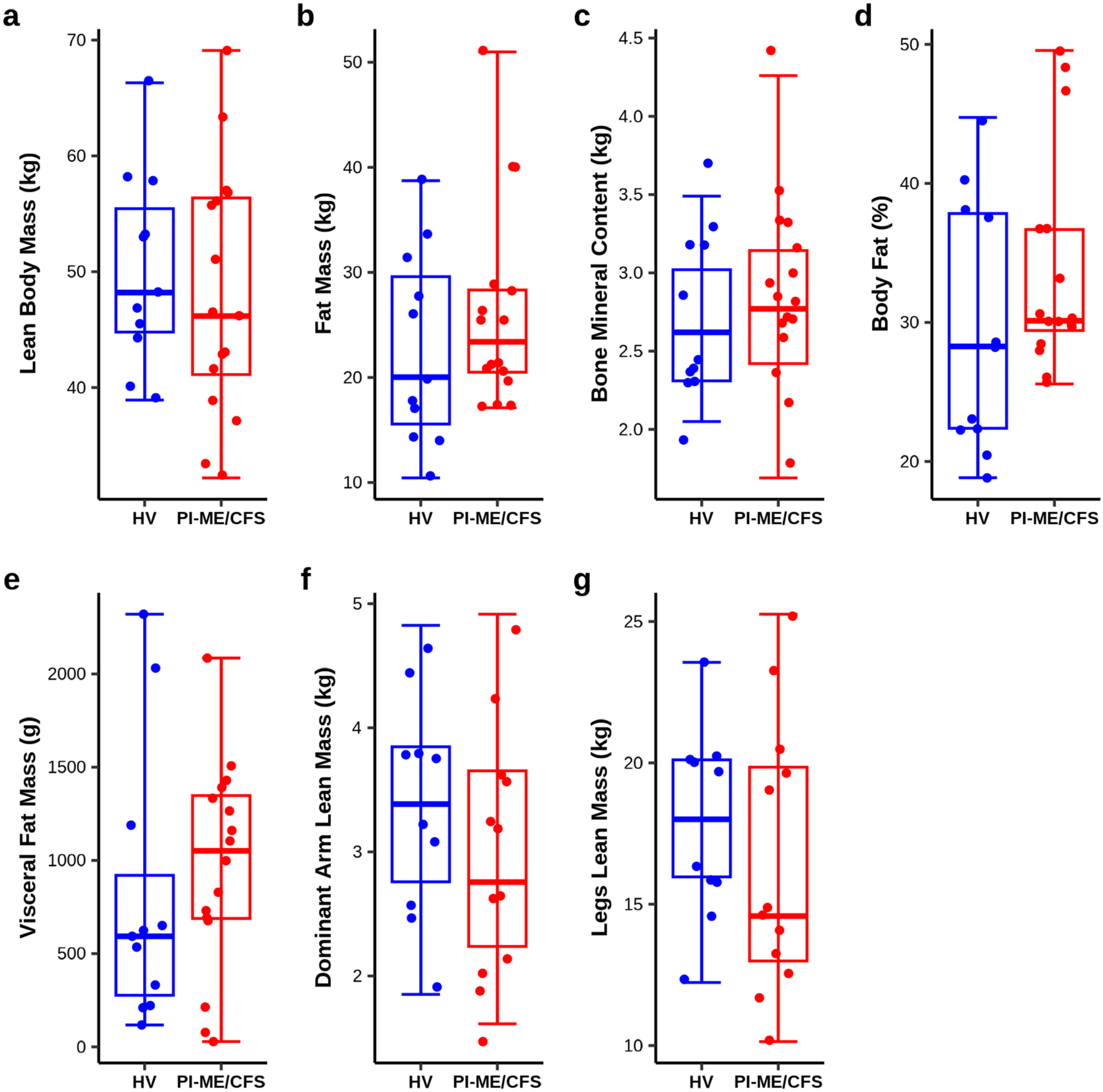
a
Lean Body Mass (kg)
70
60
50
40
HV
PI-ME/CFS
b
Fat Mass (kg)
50
40
30
20
10
HV
PI-ME/CFS
c
Bone Mineral Content (kg)
4.5
4.0
3.5
3.0
2.5
2.0
HV
PI-ME/CFS
d
Body Fat (%)
50
40
30
20
HV
PI-ME/CFS
e
Visceral Fat Mass (g)
2000
1500
1000
500
0
HV
PI-ME/CFS
f
Dominant Arm Lean Mass (kg)
5
4
3
2
HV
PI-ME/CFS
g
Legs Lean Mass (kg)
25
20
15
10
HV
PI-ME/CFS

Figure S2: Body mass composition measurements:
A-G. Boxplots of the indicated body mass composition measurements on the y axis in HV and PI-ME/CFS groups. No statistical differences were noted between groups of A-E) HVs (blue; n = 11 independent participants) and PI-ME/CFS patients (red; n = 16 independent participants) and F-G) HVs (blue; n = 10 independent participants) and PI-ME/CFS (red; n = 12 independent participants) groups. For box plots a-g boxes depict the median (horizontal line) within quartiles 1–3 (bounds of box). Whiskers extend to minimum and maximum values. Source data are provided as a Source Data file.

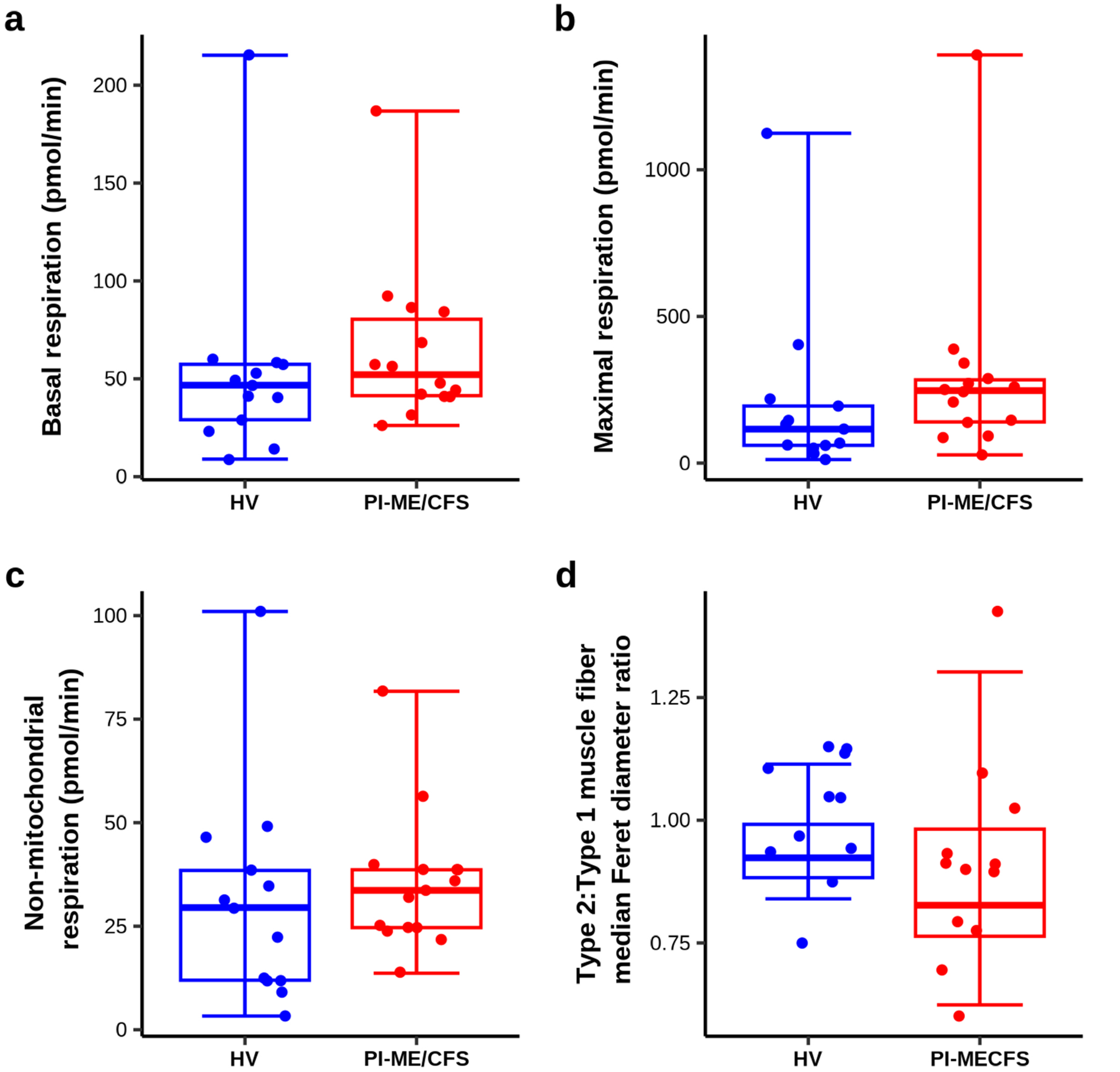

a
Basal respiration (pmol/min)
200
150
100
50
0
HV
PI-ME/CFS
b
Maximal respiration (pmol/min)
1000
500
0
HV
PI-ME/CFS
c
Non-mitochondrial respiration (pmol/min)
100
75
50
25
0
HV
PI-ME/CFS
d
Type 2:Type 1 muscle fiber median Feret diameter ratio
1.25
1.00
0.75
HV
PI-MECFS

Figure S3: Mitochondrial flux assay and Type2:Type1 ratio of muscle fibers measurements:
A-C. Boxplots of the indicated mitochondrial flux measurements on the y axis in HV (blue; n = 13 independent participants) and PI-ME/CFS (red; n = 14 independent participants) groups in A-B and C) in HVs (blue; n = 13 independent participants) and PI-ME/CFS (red; n = 15 independent participants) groups. No statistical differences were noted between groups. D. Boxplot of the Type2:Type1 muscle fiber median Feret diameter ratio obtained from biopsies of the vastus lateralis. No statistical difference was noted between groups of HVs (blue; n = 11 independent participants) and PI-ME/CFS (red; n = 12 independent participants) groups. For box plots a-d boxes depict the median (horizontal line) within quartiles 1–3 (bounds of box). Whiskers extend to minimum and maximum values. Source data are provided as a Source Data file.

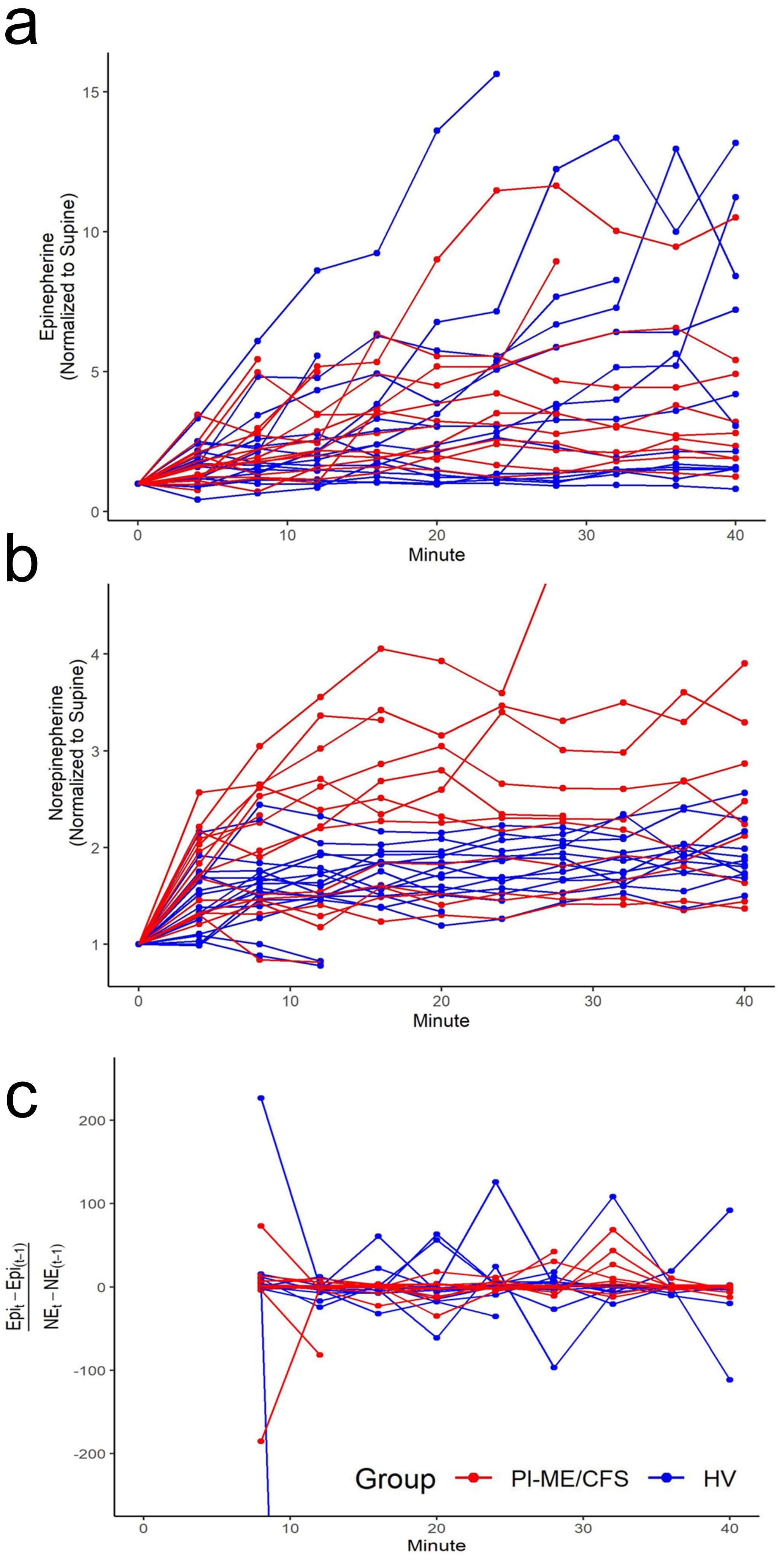
a
Epinepherine (Normalized to Supine)
Minute
b
Norepinepherine (Normalized to Supine)
Minute
c
$\frac{Epi_t - Epi_{(t-1)}}{NE_t - NE_{(t-1)}}$
Minute
Group
PI-ME/CFS
HV

Figure S4: Serial plasma catecholamine levels measures during an orthostatic challenge:
A. Plasma epinephrine levels normalized to resting value on the y axis and elapsed time on the x axis in HV (blue; n = 16 independent participants) and PI-ME/CFS (red; n = 16 independent participants). B. Plasma norepinephrine levels normalized to resting value on the y axis and elapsed time on the x axis in HV (blue; n = 16 independent participants) and PI-ME/CFS (red; n = 16 independent participants). C. Ratio of the change in Epinephrine:Norepinephrine levels on the y axis and elapsed time on the x axis in HV (blue; n = 16 independent participants) and PI-ME/CFS (red; n = 16 independent participants) volunteers. No difference in sympathoadrenal balance was noted between the groups. Abbreviations. Epi: epinephrine; NE: norepinephrine. Source data are provided as a Source Data file.

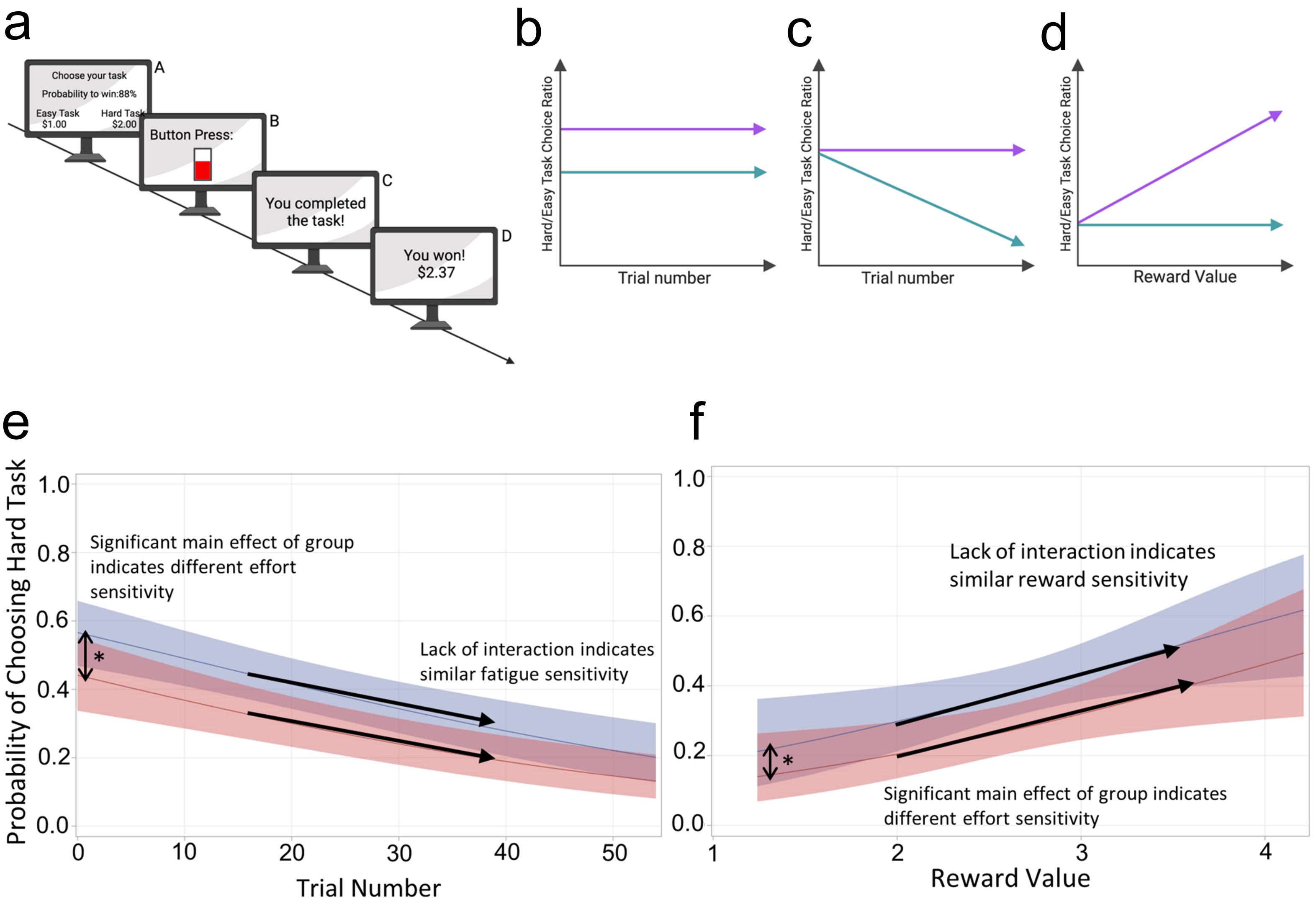
a
Choose your task
Probability to win:88%
Easy Task $1.00
Hard Task $2.00
A
Button Press:
B
You completed the task!
C
You won! $2.37
D
b
Hard/Easy Task Choice Ratio
Trial number
c
Hard/Easy Task Choice Ratio
Trial number
d
Hard/Easy Task Choice Ratio
Reward Value
e
Probability of Choosing Hard Task
1.0
0.8
0.6
0.4
0.2
0.0
Significant main effect of group indicates different effort sensitivity
Lack of interaction indicates similar fatigue sensitivity
*
0
10
20
30
40
50
Trial Number
f
1.0
0.8
0.6
0.4
0.2
0.0
Lack of interaction indicates similar reward sensitivity
Significant main effect of group indicates different effort sensitivity
*
1
2
3
4
Reward Value

Figure S5: The Effort-Expenditure for Rewards Task (EEfRT):
A. Schematic diagram of a single trial of the modified Effort Expenditure for Rewards Task ('EEfRT'). *A*. 5s choice period in which subjects are presented with information regarding the reward magnitude of the hard task for that trial and probability of receiving any reward for that trial, B. Subjects make rapid button presses to complete the chosen task for 7s (easy task) or 21s (hard task), *B*. Subjects make rapid button presses to complete the chosen task for 7s (easy task) or 21s (hard task), *C*. Subjects receive feedback on whether they have completed the task, D. Subjects receive reward feedback as to whether they received a score increase for that trial (adapted from Treadway, 2009). B-D. Examples of the interpretation of results of the binary choice EEfRT task. Trial number is on the x-axis and the hard/easy task choice ratio or reward value on the Y-axis. (B) A difference in effort sensitivity is represented by a constant reduction in hard task choices throughout the entire task, with the blue group having lower effort sensitivity than gray. (C) A difference in fatigue sensitivity is represented by a gradual decline in hard task choices as trials increase, with the blue group demonstrating complete fatigue insensitivity and the gray group demonstrating fatigue sensitivity. (D) A difference in reward sensitivity is represented by an increase in hard task choices with increasing reward value, with the blue group demonstrating reward sensitivity and the gray being completely reward-insensitive. E. Effort and fatigue sensitivity of HV (blue; n = 17 independent participants) and PI-ME/CFS (red; n = 15 independent participants). PI-ME/CFS volunteers demonstrate significantly more effort sensitivity (p=0.04) than HVs. No difference in fatigue sensitivity was noted between the groups. F. Reward sensitivity of HV (blue; n = 17 independent participants) and PI-ME/CFS (red; n = 15 independent participants). No significant difference in reward sensitivity was noted between the groups. A difference in effort sensitivity is again noted. Figure S5A created with Biorender. Source data are provided as a Source Data file.

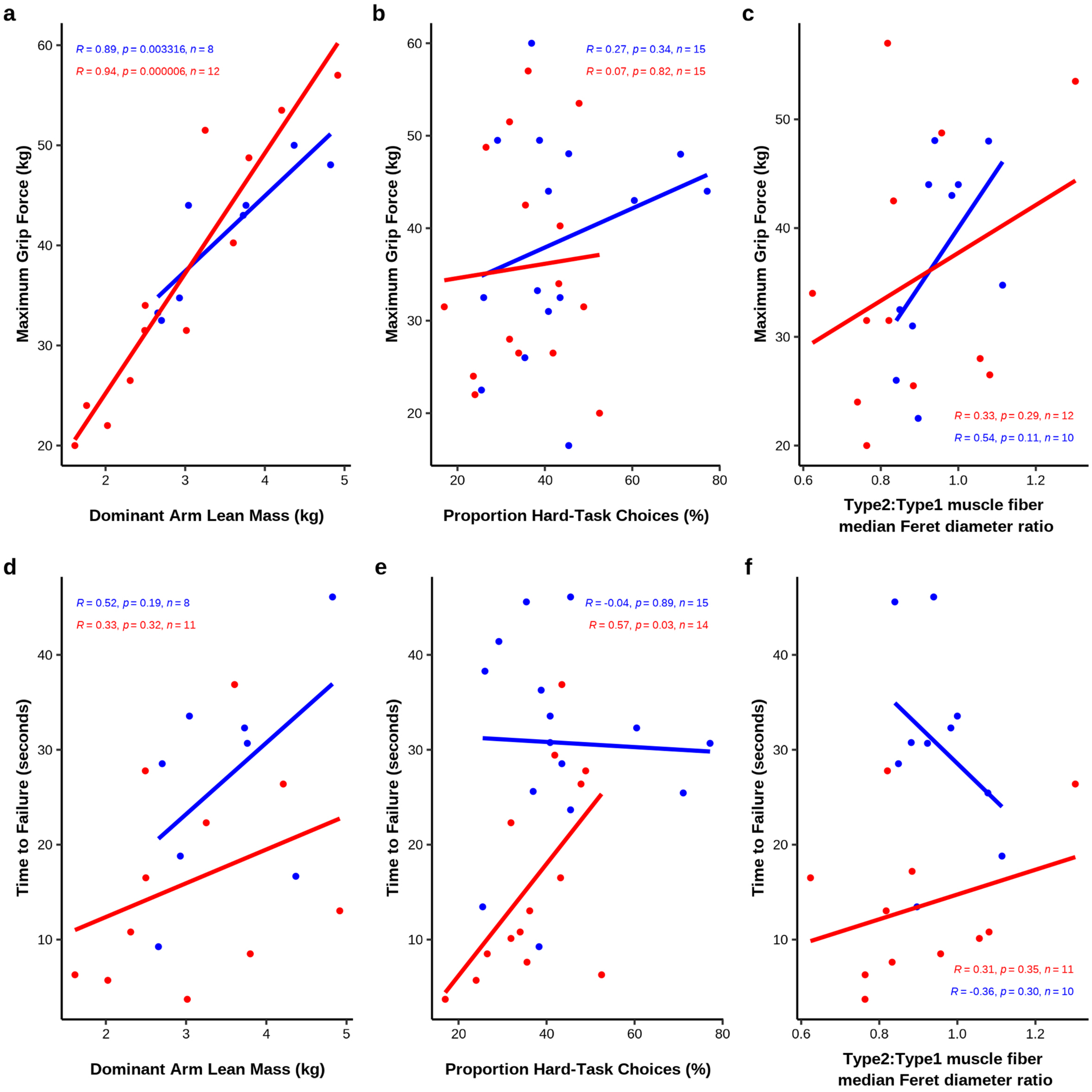

a
R = 0.89, p = 0.003316, n = 8
R = 0.94, p = 0.000006, n = 12
Maximum Grip Force (kg)
Dominant Arm Lean Mass (kg)
b
R = 0.27, p = 0.34, n = 15
R = 0.07, p = 0.82, n = 15
Maximum Grip Force (kg)
Proportion Hard-Task Choices (%)
c
R = 0.33, p = 0.29, n = 12
R = 0.54, p = 0.11, n = 10
Maximum Grip Force (kg)
Type2:Type1 muscle fiber median Feret diameter ratio
d
R = 0.52, p = 0.19, n = 8
R = 0.33, p = 0.32, n = 11
Time to Failure (seconds)
Dominant Arm Lean Mass (kg)
e
R = -0.04, p = 0.89, n = 15
R = 0.57, p = 0.03, n = 14
Time to Failure (seconds)
Proportion Hard-Task Choices (%)
f
R = 0.31, p = 0.35, n = 11
R = -0.36, p = 0.30, n = 10
Time to Failure (seconds)
Type2:Type1 muscle fiber median Feret diameter ratio

Figure S6: Correlations between single grip task performance and lean arm mass, effort preference, and Type2:Type1 median Feret diameter ratio of muscle fibers
A-C. Correlation between maximum grip force on the x axis and (A) dominant lean arm mass in HVs (blue; n = 8 independent participants) and PI-ME/CFS (red; n = 12 independent participants) groups, (B) proportion of hard task choices (i.e. effort preference) in HVs (blue; n = 15 independent participants) and PI-ME/CFS (red; n = 15 independent participants) groups, and (C) Type2:Type1 muscle fiber median Feret diameter ratio for HV (blue; n = 10 independent participants) and PI-ME/CFS (red; n = 12 independent participants) volunteers. D-F. Correlation between Time to Failure on the x axis and (D) dominant lean arm mass in HVs (blue; n = 8 independent participants) and PI-ME/CFS (red; n = 11 independent participants) groups, (E) proportion of hard task choices (i.e. effort preference) in HVs (blue; n = 15 independent participants) and PI-ME/CFS (red; n = 14 independent participants) groups, and (F) Type2:Type1 muscle fiber median Feret diameter ratio for HV (blue; n = 10 independent participants) and PI-ME/CFS (red; n = 11 independent participants) volunteers. The relationship between indicated variables in x and y axis were fitted by linear regression in each group with a linear regression t-test to determine non-zero slope. The exact p value of the regressions are presented on the graph. Source data are provided as a Source Data file.

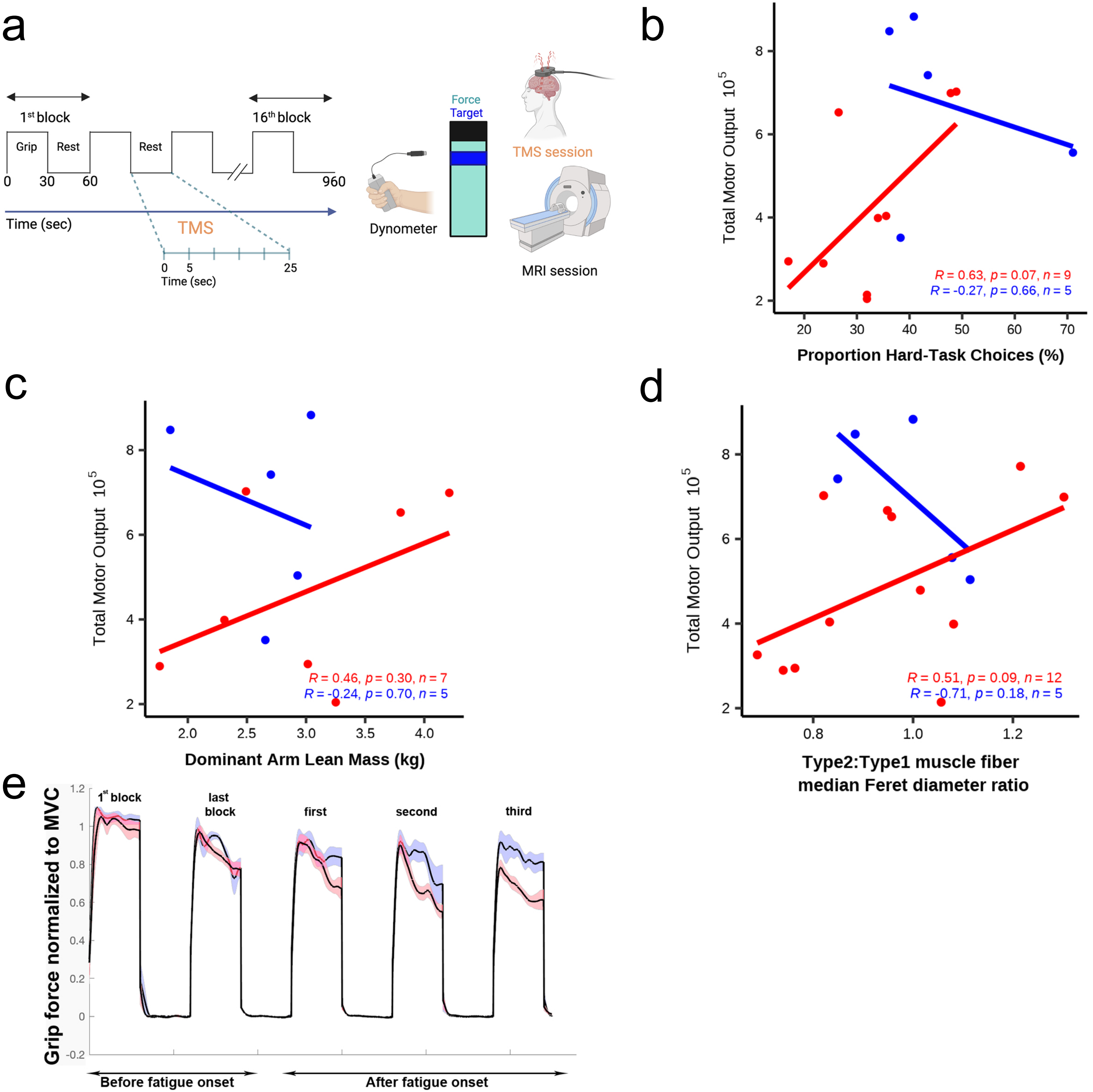

a
1st block
16th block
Grip
Rest
Rest
0
30
60
960
Time (sec)
TMS
0
5
25
Time (sec)
Dynometer
Force
Target
TMS session
MRI session
b
Total Motor Output 10^5
Proportion Hard-Task Choices (%)
R = 0.63, p = 0.07, n = 9
R = -0.27, p = 0.66, n = 5
c
Total Motor Output 10^5
Dominant Arm Lean Mass (kg)
R = 0.46, p = 0.30, n = 7
R = -0.24, p = 0.70, n = 5
d
Total Motor Output 10^5
Type2:Type1 muscle fiber
median Feret diameter ratio
R = 0.51, p = 0.09, n = 12
R = -0.71, p = 0.18, n = 5
e
Grip force normalized to MVC
1st block
last block
first
second
third
Before fatigue onset
After fatigue onset

Figure S7: Repetitive grip strength testing and correlations between total motor output and lean arm mass, effort preference, and Type2:Type1 ratio of muscle fibers
A. Schema of repetitive grip strength testing. Volunteers sought to maintain their grip at 50% of maximum voluntary contraction in successive blocks of 30 seconds interspaced with 30 second rest blocks. For the transcranial magnetic stimulation (TMS) experiment, single pulse TMS was done to probe excitability of the primary motor cortex (M1) via the motor evoked potential (MEP) of the abductor pollicis brevis during the rest blocks. For the functional magnetic resonance imaging (fMRI) experiment, the paradigm was performed while undergoing MRI scanning of the brain. B-D. Correlation between total motor output on the x axis and (B) proportion of hard tasks choices (i.e. effort preference) in HVs (blue; n = 5 independent participants) and PI-ME/CFS (red; n = 9 independent participants) groups, (C) dominant lean arm mass in HVs (blue; n = 5 independent participants) and PI-ME/CFS (red; n = 7 independent participants) groups, and (D) Type2:Type1 muscle fiber median Feret diameter ratio for HV (blue; n = 5 independent participants) and PI-ME/CFS (red; n = 12 independent participants) volunteers during the TMS experiment. E. Grip force during fMRI task normalized to maximum voluntary contraction in the first block, the last block prior to fatigue onset, and the first three blocks after fatigue onset in HV (blue; n = 10 independent participants) and PI-ME/CFS cohorts (red; n = 8 independent participants). A significant difference was noted between the groups (Decline rate: -6.3 ±5 versus -11.9 ±3, $t(16) = 2.83$, $p = 0.01$). Figure S7A created with Biorender. Source data are provided as a Source Data file.

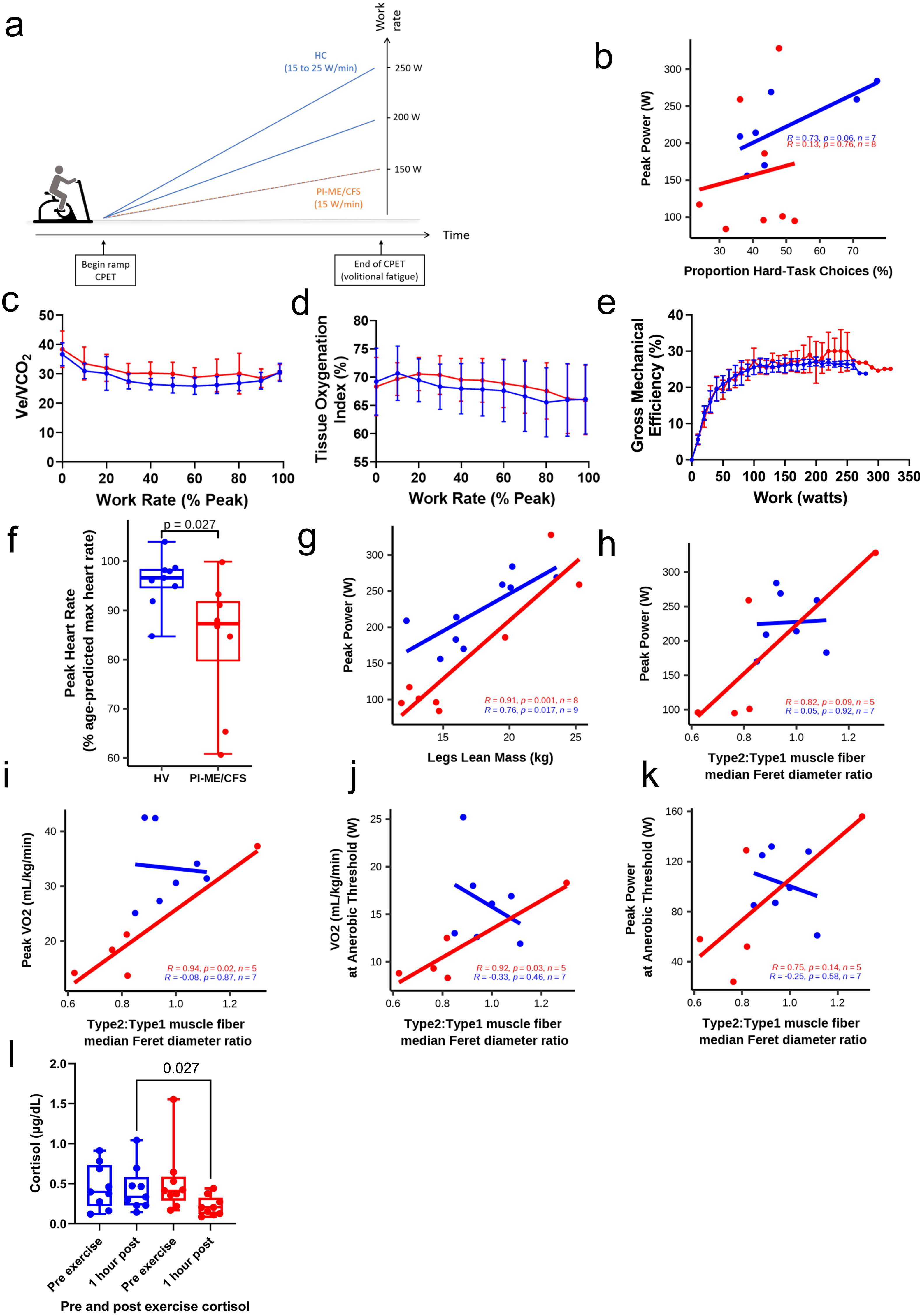

a
Work rate
HC
(15 to 25 W/min)
250 W
200 W
150 W
PI-ME/CFS
(15 W/min)
Time
Begin ramp CPET
End of CPET (volitional fatigue)
b
Peak Power (W)
Proportion Hard-Task Choices (%)
R = 0.73, p = 0.06, n = 7
R = 0.13, p = 0.76, n = 8
c
Ve/VCO2
Work Rate (% Peak)
d
Tissue Oxygenation Index (%)
Work Rate (% Peak)
e
Gross Mechanical Efficiency (%)
Work (watts)
f
Peak Heart Rate (% age-predicted max heart rate)
p = 0.027
HV
PI-ME/CFS
g
Peak Power (W)
Legs Lean Mass (kg)
R = 0.91, p = 0.001, n = 8
R = 0.76, p = 0.017, n = 9
h
Peak Power (W)
Type2:Type1 muscle fiber median Feret diameter ratio
R = 0.82, p = 0.09, n = 5
R = 0.05, p = 0.92, n = 7
i
Peak VO2 (mL/kg/min)
Type2:Type1 muscle fiber median Feret diameter ratio
R = 0.94, p = 0.02, n = 5
R = -0.08, p = 0.87, n = 7
j
VO2 (mL/kg/min) at Anerobic Threshold (W)
Type2:Type1 muscle fiber median Feret diameter ratio
R = 0.92, p = 0.03, n = 5
R = -0.33, p = 0.46, n = 7
k
Peak Power at Anerobic Threshold (W)
Type2:Type1 muscle fiber median Feret diameter ratio
R = 0.75, p = 0.14, n = 5
R = -0.25, p = 0.58, n = 7
l
Cortisol (µg/dL)
0.027
Pre exercise
1 hour post
Pre exercise
1 hour post
Pre and post exercise cortisol

Figure S8: Cardiopulmonary Exercise Test (CPET)
A. Schema of CPET. Upright cycle ergometry was used to evaluate cardiorespiratory function. To achieve the peak work rate during a testing time of 8-12 minutes, groups were tested at 15 Watts/min (PI-ME/CFS) and 15-25 Watts/min (HV). Testing was ended when volunteers were unable to keep up with the increasing work rate. B. Correlation between peak power and proportion of hard task choices for HV and PI-ME/CFS volunteers. C. Ratio of Ventilation/VCO_2 with peak work (%) for HV (blue; n = 9 independent participants) and PI-ME/CFS (red; n = 8 independent participants) volunteers. D. Oxygenation of the quadriceps muscle measured with Near Infrared Spectroscopy with work rate (% of peak) for HV (blue; n = 6 independent participants) and PI-ME/CFS (red; n = 6 independent participants) volunteers. E. Gross mechanical efficiency (%) with work (Watts) for HV (blue; n = 9 independent participants) and PI-ME/CFS (red; n = 8 independent participants) volunteers. F. Boxplot of % age predicted heart rate for HV (blue; n = 9 independent participants) and PI-ME/CFS (red; n = 8 independent participants) volunteers using unadjusted two-sided t-test for independent samples with equal variance (p = 0.042). For F, boxes depict the median (horizontal line) within quartiles 1–3 (bounds of box). Whiskers extend to minimum and maximum values. G-H. Correlation between peak power and (G) lean leg mass and (H) Type2:Type1 muscle fiber median Feret diameter ratio for HV and PI-ME/CFS volunteers. I-K. Correlation between Type2:Type1 muscle fiber median Feret diameter ratio and (I) peak $V0_2$, (J) VO2 at the anaerobic threshold, and (K) peak power at the anaerobic threshold for HV and PI-ME/CFS volunteers. For figures B and G-K, the relationship between indicated variables were fitted by linear regression in each group with linear regression t-tests used to determine significant correlations. The exact p value and sample sizes are presented on the graph. L. Salivary cortisol levels measured the morning prior to CPET and one hour after CPET for HV (blue; n = 11 independent participants) and PI-ME/CFS (red; n = 15 independent participants) volunteers. Source data are provided as a Source Data file.

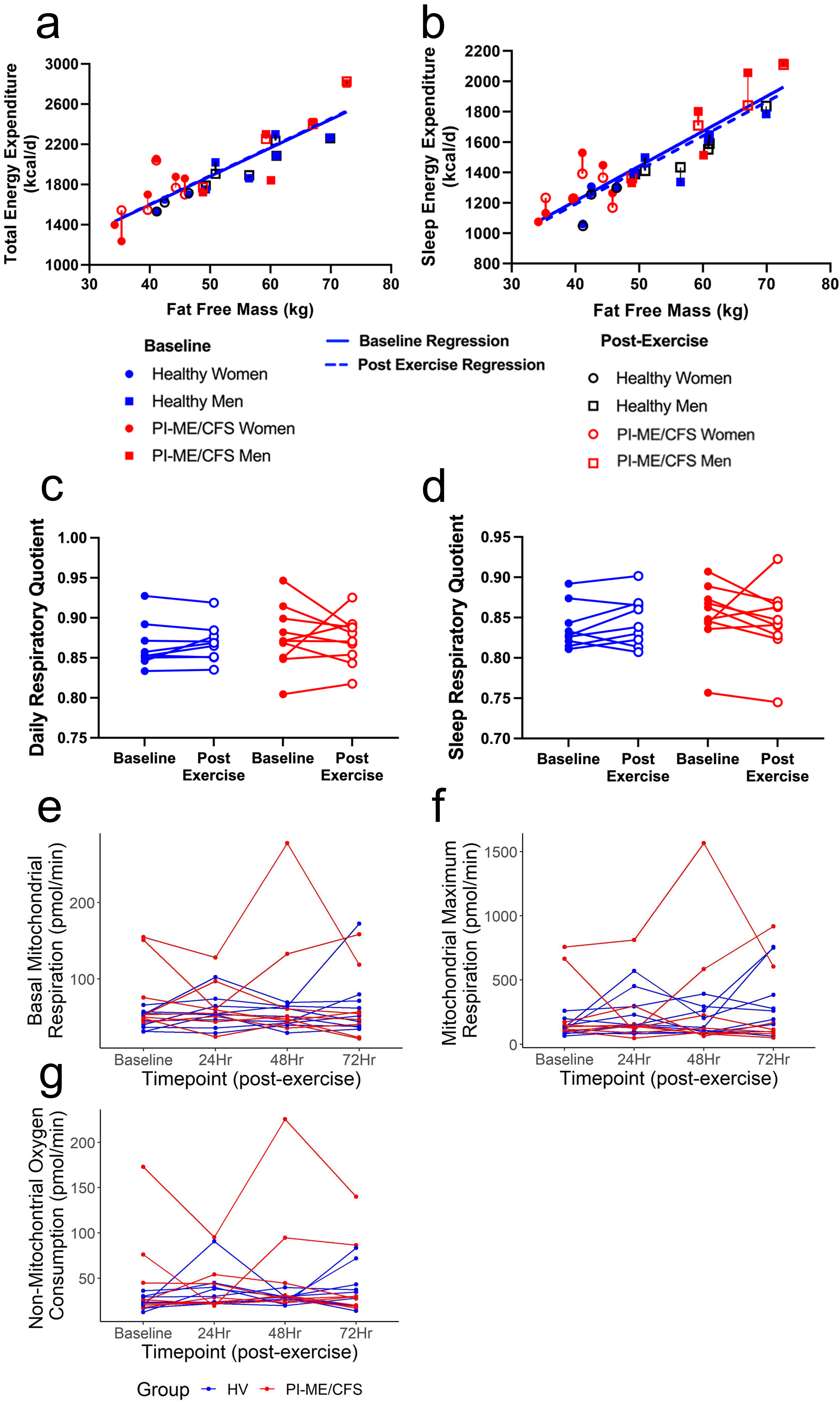

a
b
Total Energy Expenditure (kcal/d)
Sleep Energy Expenditure (kcal/d)
Fat Free Mass (kg)
Baseline
Healthy Women
Healthy Men
PI-ME/CFS Women
PI-ME/CFS Men
Baseline Regression
Post Exercise Regression
Post-Exercise
Healthy Women
Healthy Men
PI-ME/CFS Women
PI-ME/CFS Men
c
d
Daily Respiratory Quotient
Sleep Respiratory Quotient
Baseline
Post Exercise
Baseline
Post Exercise
e
f
Basal Mitochondrial Respiration (pmol/min)
Mitochondrial Maximum Respiration (pmol/min)
Baseline
24Hr
48Hr
72Hr
Timepoint (post-exercise)
g
Non-Mitochondrial Oxygen Consumption (pmol/min)
Group
HV
PI-ME/CFS

Figure S9: Total body energy expenditure, sleeping energy expenditure, and respiratory quotient measurements at baseline and 48 hours after cardiopulmonary exercise testing (CPET) A-B. Correlation of fat free mass on the x axis with (A) total body energy expenditure and (B) sleeping energy expenditure on the y axis for HV (blue; n = 10 independent participants) and PI-ME/CFS (red; n = 14 independent participants). Squares represent males and circles represent females. Solid data points are baseline measurements, open data points are measurements 48 hours after CPET. No statistically significant differences in total or sleeping energy expenditure was noted between the groups. C-D. Respiratory quotient measurements taken (C) daily and (D) sleeping at baseline and 48 hours after CPET for HV (blue; n = 9 independent participants) and PI-ME/CFS (red; n = 10 independent participants). E-G. Serial mitochondrial flux assay measurements of peripheral blood mononuclear cells immediate before (baseline), 24, 48, and 72 hours after CPET for HV (blue; n = 9 independent participants) and PI-ME/CFS (red; n = 8 independent participants). No differences were observed in (E) basal, (F) maximum respiration, and (G) non-mitochondrial oxygen consumption between the groups. Source data are provided as a Source Data file.

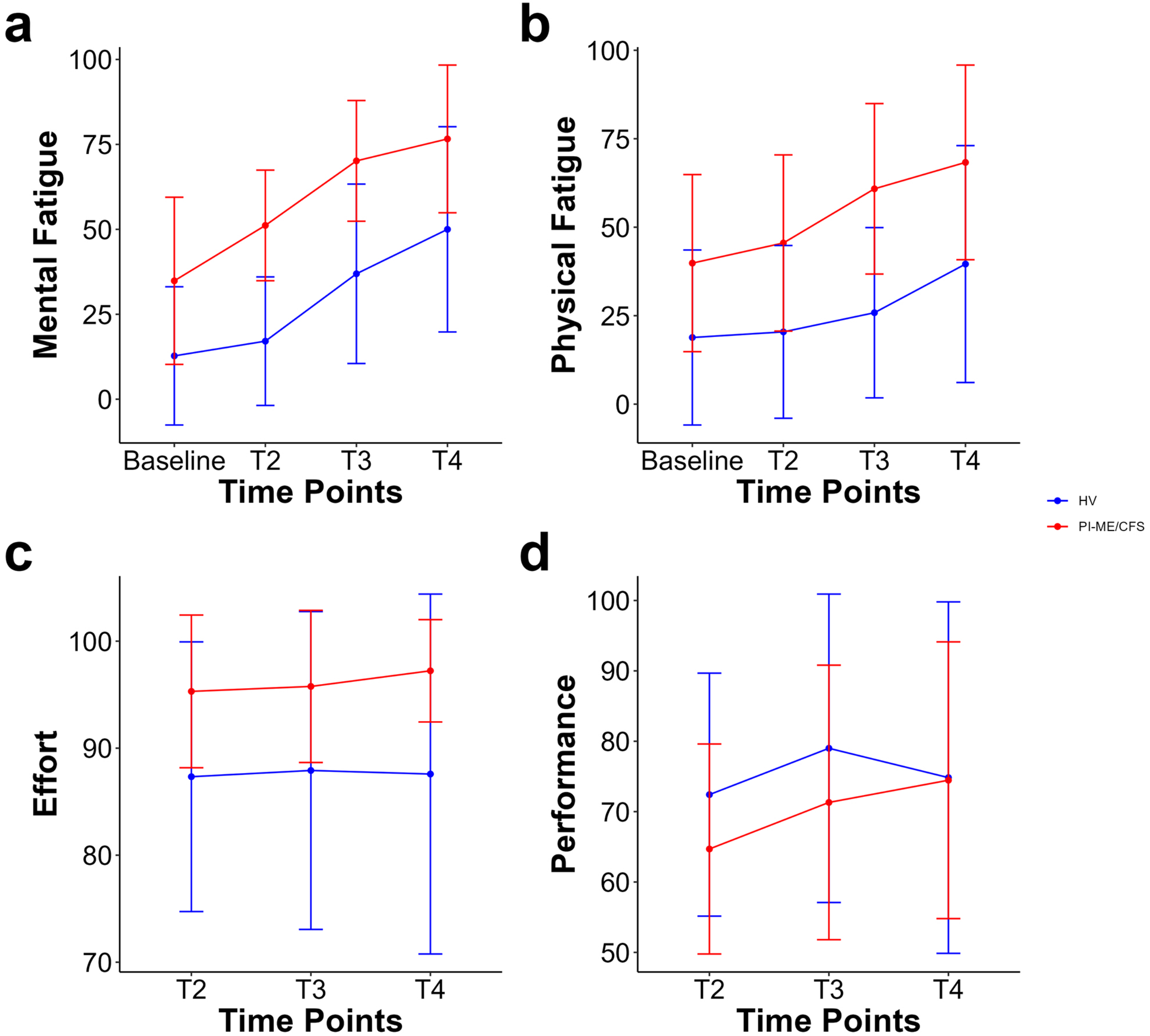
a
Mental Fatigue
100
75
50
25
0
Baseline
T2
T3
T4
Time Points
b
Physical Fatigue
100
75
50
25
0
Baseline
T2
T3
T4
Time Points
HV
PI-ME/CFS
c
Effort
100
90
80
70
T2
T3
T4
Time Points
d
Performance
100
90
80
70
60
50
T2
T3
T4
Time Points

Figure S10: Serial visual analog scales of mental and physical fatigue, perceived effort, and perceived performance during a cognitive testing battery
A-D. Visual analog scale from 0 -100 points of (A) mental fatigue, (B) physical fatigue, (C) perceived effort, and (D) perceived performance on the y axis and elapsed time on the x axis for HV (blue; n = 12 independent participants) and PI-ME/CFS (red; n = 13 independent participants). Each time point represents approximately one elapsed hour. Data represented as means and standard deviations. Source data are provided as a Source Data file.

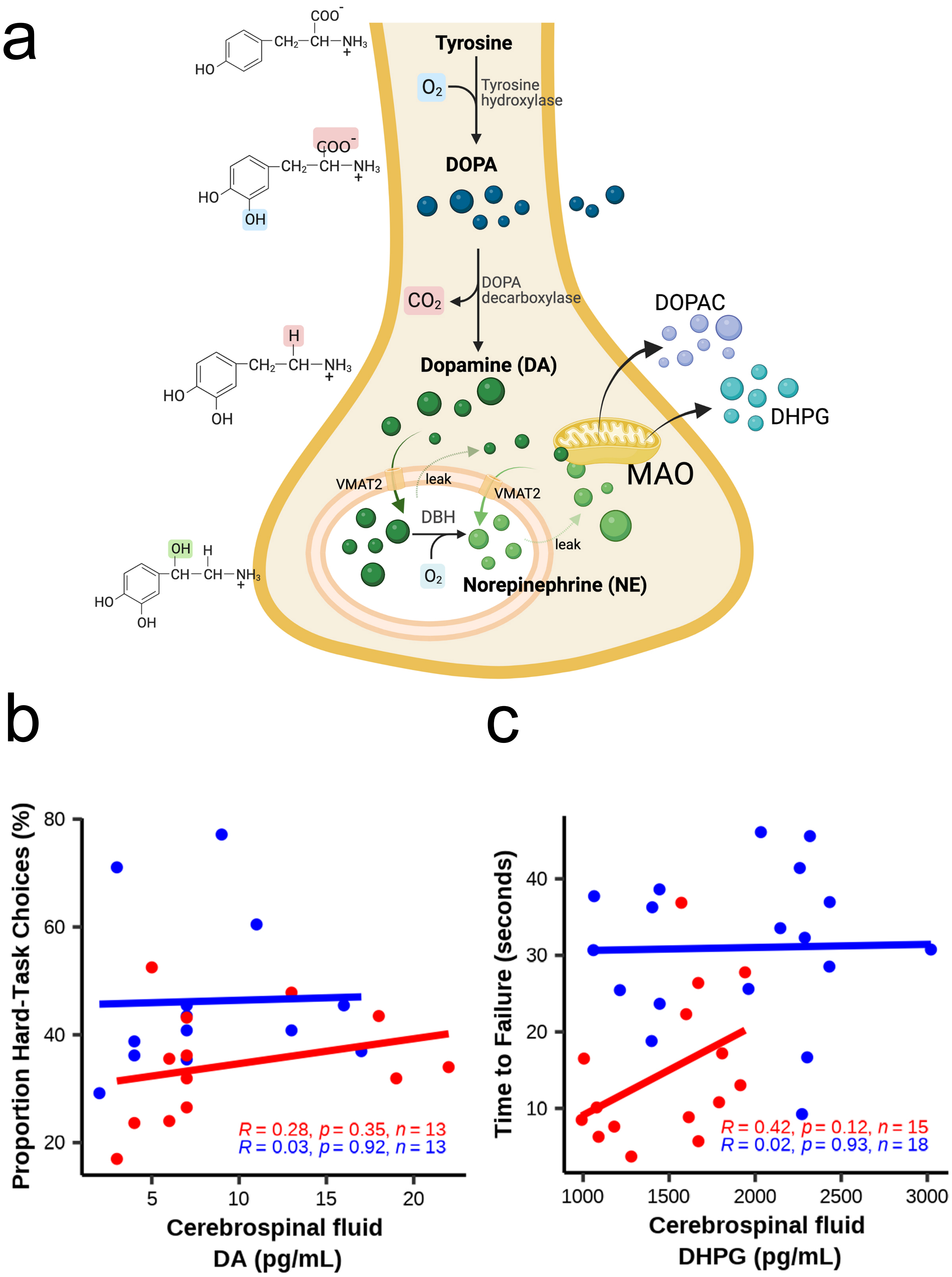

a
Tyrosine
O_2
Tyrosine hydroxylase
DOPA
CO_2
DOPA decarboxylase
Dopamine (DA)
DOPAC
DHPG
MAO
VMAT2
leak
VMAT2
DBH
leak
O_2
Norepinephrine (NE)
b
Proportion Hard-Task Choices (%)
Cerebrospinal fluid DA (pg/mL)
R = 0.28, p = 0.35, n = 13
R = 0.03, p = 0.92, n = 13
c
Time to Failure (seconds)
Cerebrospinal fluid DHPG (pg/mL)
R = 0.42, p = 0.12, n = 15
R = 0.02, p = 0.93, n = 18

Figure S11: Correlations between cerebrospinal fluid catecholamines with time to failure on the single grip task and effort preference.
A. Concept diagram relating cerebrospinal levels of catechols to intra-neuronal reactions and reactants in central catecholaminergic neurons. DOPAC and DHPG are the main respective neuronal metabolites of DA and NE. Alterations in processes within catecholaminergic neurons produce predictable patterns of cerebrospinal levels of catechols. B. Correlation of cerebrospinal fluid dopamine (DA) with proportion of hard task choices (i.e., effort preference) for HV (blue; n = 13 independent participants) and PI-ME/CFS participants (red; n = 13 independent participants). C. Correlation of cerebrospinal fluid dihydroxyphenylglycol (DHPG) with Time to failure in HVs (blue; n = 14 independent participants) and PI-ME/CFS (red; n = 14 independent participants) groups. The relationship between indicated variables in x and y axis were fitted by linear regression. The exact p value of the regression is presented on the graph. Abbreviations.TH: Tyrosine hydroxylase, DOPA: Precursor to dopamine, LAAAD: L-aromatic-amino-acid decarboxylase, DA: Dopamine, DBH: Dopamine beta-hydroxylase, NE: Norepinephrine, VMAT: Vesicular monoamine transporter, MAO: Monoamine oxidase, ALDH: Aldehyde dehydrogenase, AR: Androgen Receptor, DOPAC: 3,4-Dihydroxyphenylacetic acid, DHPG: (S)-3,5-Dihydroxyphenylglycine. Figure 11A created with Biorender. Source data are provided as a Source Data file.

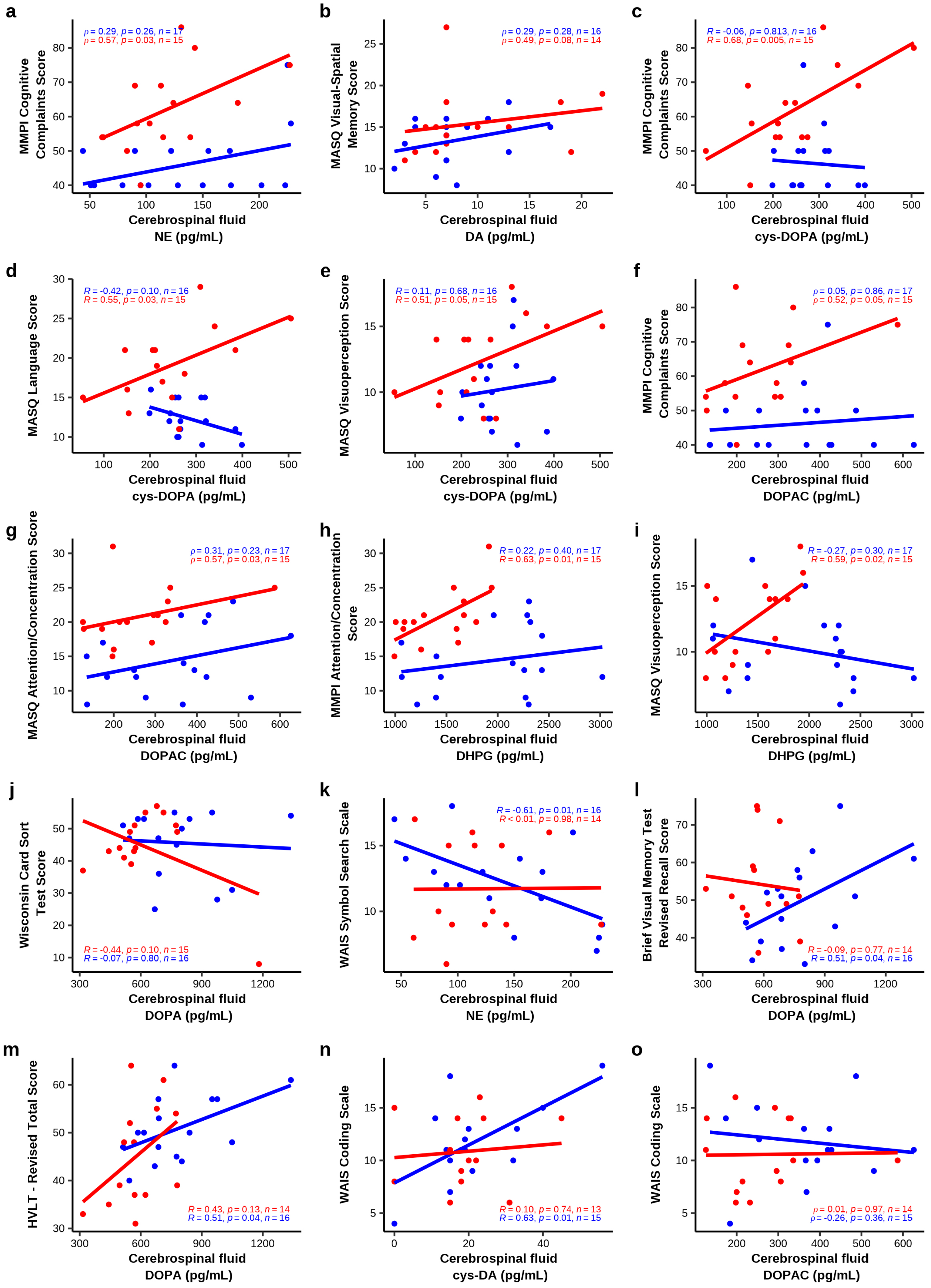

a
MMPI Cognitive Complaints Score
ρ = 0.29, p = 0.26, n = 17
ρ = 0.57, p = 0.03, n = 15
Cerebrospinal fluid NE (pg/mL)
b
MASQ Visual-Spatial Memory Score
ρ = 0.29, p = 0.28, n = 16
ρ = 0.49, p = 0.08, n = 14
Cerebrospinal fluid DA (pg/mL)
c
MMPI Cognitive Complaints Score
R = -0.06, p = 0.813, n = 16
R = 0.68, p = 0.005, n = 15
Cerebrospinal fluid cys-DOPA (pg/mL)
d
MASQ Language Score
R = -0.42, p = 0.10, n = 16
R = 0.55, p = 0.03, n = 15
Cerebrospinal fluid cys-DOPA (pg/mL)
e
MASQ Visuoperception Score
R = 0.11, p = 0.68, n = 16
R = 0.51, p = 0.05, n = 15
Cerebrospinal fluid cys-DOPA (pg/mL)
f
MMPI Cognitive Complaints Score
ρ = 0.05, p = 0.86, n = 17
ρ = 0.52, p = 0.05, n = 15
Cerebrospinal fluid DOPAC (pg/mL)
g
MASQ Attention/Concentration Score
ρ = 0.31, p = 0.23, n = 17
ρ = 0.57, p = 0.03, n = 15
Cerebrospinal fluid DOPAC (pg/mL)
h
MMPI Attention/Concentration Score
R = 0.22, p = 0.40, n = 17
R = 0.63, p = 0.01, n = 15
Cerebrospinal fluid DHPG (pg/mL)
i
MASQ Visuoperception Score
R = -0.27, p = 0.30, n = 17
R = 0.59, p = 0.02, n = 15
Cerebrospinal fluid DHPG (pg/mL)
j
Wisconsin Card Sort Test Score
R = -0.44, p = 0.10, n = 15
R = -0.07, p = 0.80, n = 16
Cerebrospinal fluid DOPA (pg/mL)
k
WAIS Symbol Search Scale
R = -0.61, p = 0.01, n = 16
R < 0.01, p = 0.98, n = 14
Cerebrospinal fluid NE (pg/mL)
l
Brief Visual Memory Test Revised Recall Score
R = -0.09, p = 0.77, n = 14
R = 0.51, p = 0.04, n = 16
Cerebrospinal fluid DOPA (pg/mL)
m
HVLT - Revised Total Score
R = 0.43, p = 0.13, n = 14
R = 0.51, p = 0.04, n = 16
Cerebrospinal fluid DOPA (pg/mL)
n
WAIS Coding Scale
R = 0.10, p = 0.74, n = 13
R = 0.63, p = 0.01, n = 15
Cerebrospinal fluid cys-DA (pg/mL)
o
WAIS Coding Scale
ρ = 0.01, p = 0.97, n = 14
ρ = -0.26, p = 0.36, n = 15
Cerebrospinal fluid DOPAC (pg/mL)

Figure S12: Correlations between cerebrospinal fluid catecholamines with cognitive complaints and performance on neuropsychological tests
A. Correlation of cerebrospinal fluid norepinephrine (NE) with total cognitive complaints score for HV (blue) and PI-ME/CFS (red). B. Correlation of cerebrospinal fluid dopamine (DA) with visual-spatial memory score for HV and PI-ME/CFS. C-E. Correlation of cerebrospinal fluid cys-DOPA with (C) total cognitive complaints, (D) language, and (E) visuoperception scores for HV and PI-ME/CFS. F-G. Correlation of cerebrospinal fluid dopamine DOPAC with (F) total cognitive complaints and (G) attention/concentration scores for HV and PI-ME/CFS. H-I. Correlation of cerebrospinal fluid DHPG with (H) attention/concentration complaints and (I) visuoperception scores for HV and PI-ME/CFS. J. Correlation of cerebrospinal fluid DOPA with the Wisconsin Card Sort Test score for HV and PI-ME/CFS. K. Correlation of cerebrospinal fluid norepinephrine (NE) with WAIS Symbol Search Scale score for HV and PI-ME/CFS. L-M. Correlation of cerebrospinal fluid DOPA with (L) Brief Visual Memory Test-Revised Recall and (M) Hopkins Verbal Learning Test-Revised Total scores for HV and PI-ME/CFS. N. Correlation of cerebrospinal fluid cys-dopamine with the WAIS Coding Scale for HV and PI-ME/CFS. O. Correlation of cerebrospinal fluid DOPAC with the WAIS Coding Scale for HV and PI-ME/CFS. The relationship between indicated variables in x and y axis were fitted by linear regression with linear regression t-tests used to determine non-zero slope. The exact p values, correlation coefficients, and sample sizes are presented in each panel of the figure for the respective groups. Correlation was determined by performing a Shapiro-Wilk test to determine normality of the data and then a Pearson correlation was used for normally distributed data and a Spearman correlation was used for non-normally distributed data. Source data are provided as a Source Data file.

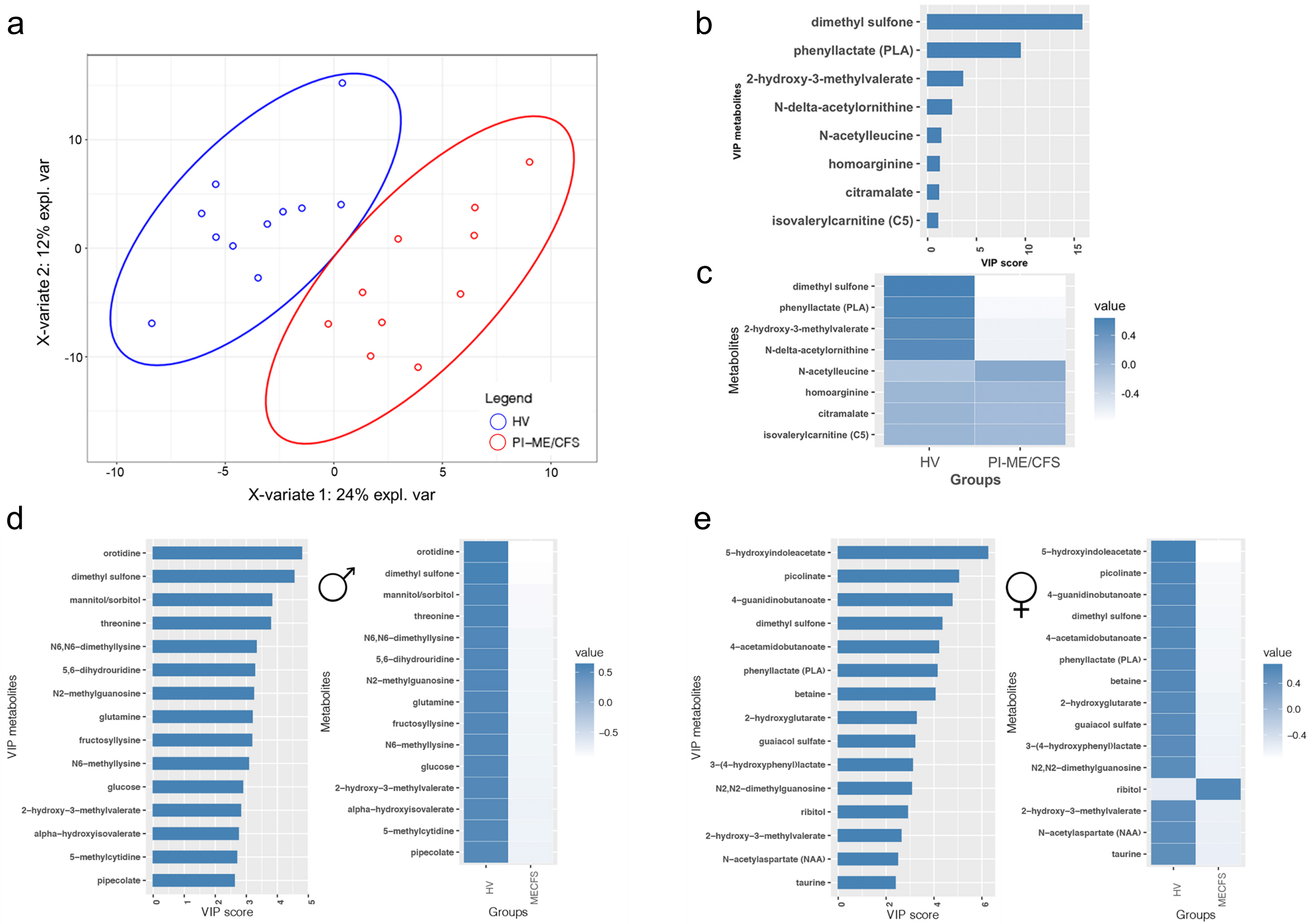
a
X-variate 2: 12% expl. var
X-variate 1: 24% expl. var
Legend
HV
PI–ME/CFS
b
dimethyl sulfone
phenyllactate (PLA)
2-hydroxy-3-methylvalerate
N-delta-acetylornithine
N-acetylleucine
homoarginine
citramalate
isovalerylcarnitine (C5)
VIP metabolites
VIP score
c
Metabolites
Groups
HV
PI-ME/CFS
value
d
orotidine
dimethyl sulfone
mannitol/sorbitol
threonine
N6,N6–dimethyllysine
5,6–dihydrouridine
N2–methylguanosine
glutamine
fructosyllysine
N6–methyllysine
glucose
2–hydroxy–3–methylvalerate
alpha–hydroxyisovalerate
5–methylcytidine
pipecolate
VIP metabolites
VIP score
Metabolites
Groups
MECFS
e
5–hydroxyindoleacetate
picolinate
4–guanidinobutanoate
4–acetamidobutanoate
betaine
2–hydroxyglutarate
guaiacol sulfate
3–(4–hydroxyphenyl)lactate
N2,N2–dimethylguanosine
ribitol
N–acetylaspartate (NAA)
taurine

Figure S13: Multivariate analysis of the metabolomics on the cerebrospinal fluid (CSF) samples in HV and PI-ME/CFS cohorts:
A. Final partial least square discriminant analysis (PLSDA) supervised clustering showing HV (blue; n = 21 independent participants) and PI-ME/CFS (red; n = 17 independent participants) sample groups. B. Variable importance in prediction (VIP) scores of metabolites with VIP score >1. C. The heatmap of the average scaled expression values for the indicated VIP metabolites. D-E. Variable importance in prediction (VIP) scores of the metabolites after PLSDA multivariate analysis and the heatmap of the average scaled expression values for the indicated VIP metabolites, in (D) male cohorts (HV n = 10 independent participants and PI-ME/CFS n = 7 independent participants) and (E) female cohorts (HV n = 11 independent participants and PI-ME/CFS n = 10 independent participants). Source data are provided as a Source Data file.

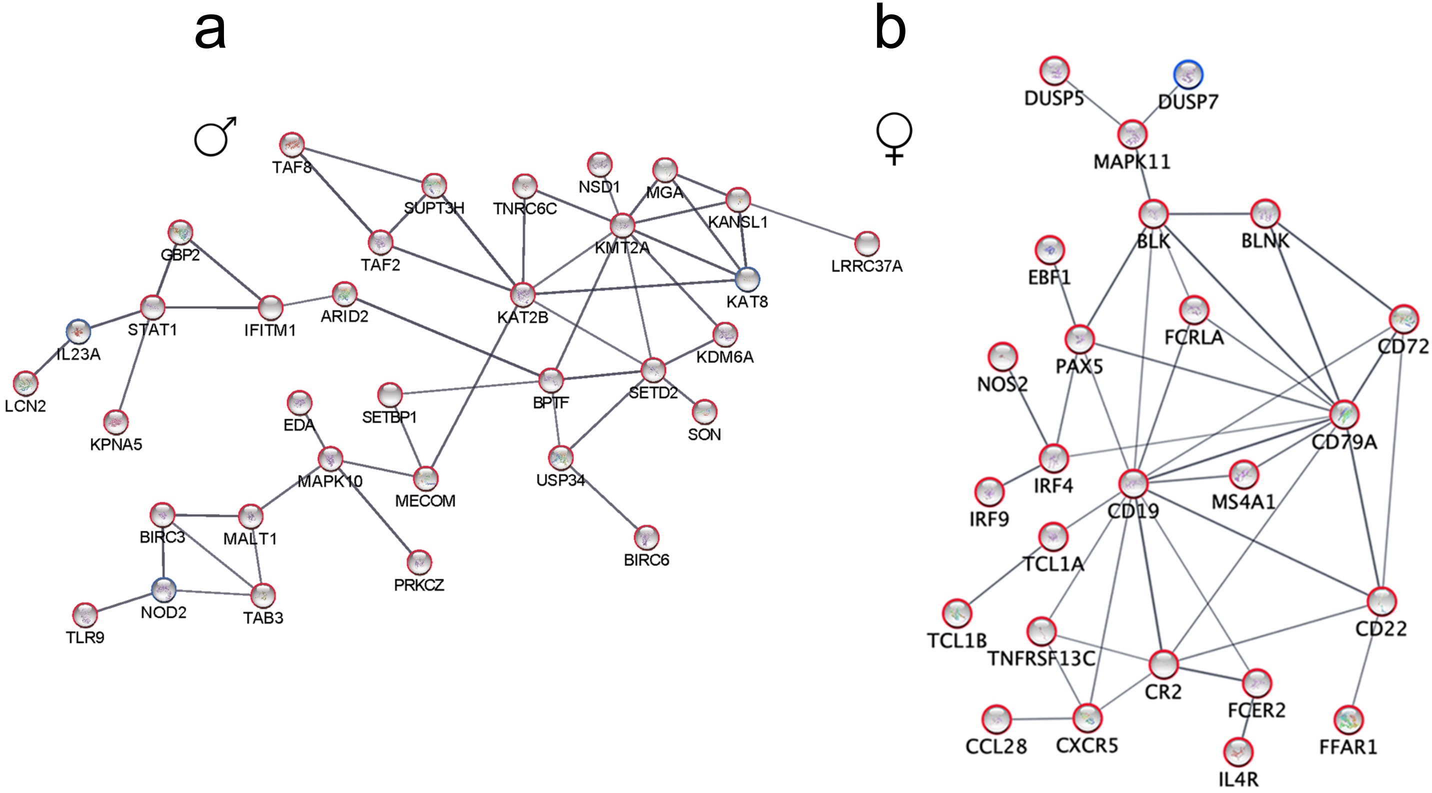
a
♂
TAF8
SUPT3H
TAF2
TNRC6C
NSD1
MGA
KANSL1
KMT2A
LRRC37A
KAT8
KAT2B
GBP2
STAT1
IFITM1
ARID2
IL23A
LCN2
KPNA5
KDM6A
SETD2
BPTF
SETBP1
EDA
SON
MAPK10
MECOM
USP34
BIRC3
MALT1
NOD2
TAB3
TLR9
PRKCZ
BIRC6
b
♀
DUSP5
DUSP7
MAPK11
BLK
BLNK
EBF1
FCRLA
CD72
PAX5
NOS2
CD79A
IRF4
IRF9
CD19
MS4A1
TCL1A
TCL1B
TNFRSF13C
CD22
CR2
FCER2
CCL28
CXCR5
FFAR1
IL4R

Figure S14: Male and female cohorts have distinct perturbation in immune cell subpopulation and biological processes in PBMCs:
A-B. Protein-Protein interactome of a subset of DE genes from PBMC samples analyzed in the (A) male and (B) female (cohorts and the fold change information are overlaid on the nodes. Red color indicates upregulation and blue color indicated downregulation in PI-ME/CFS group. Source data are provided as a Source Data file.

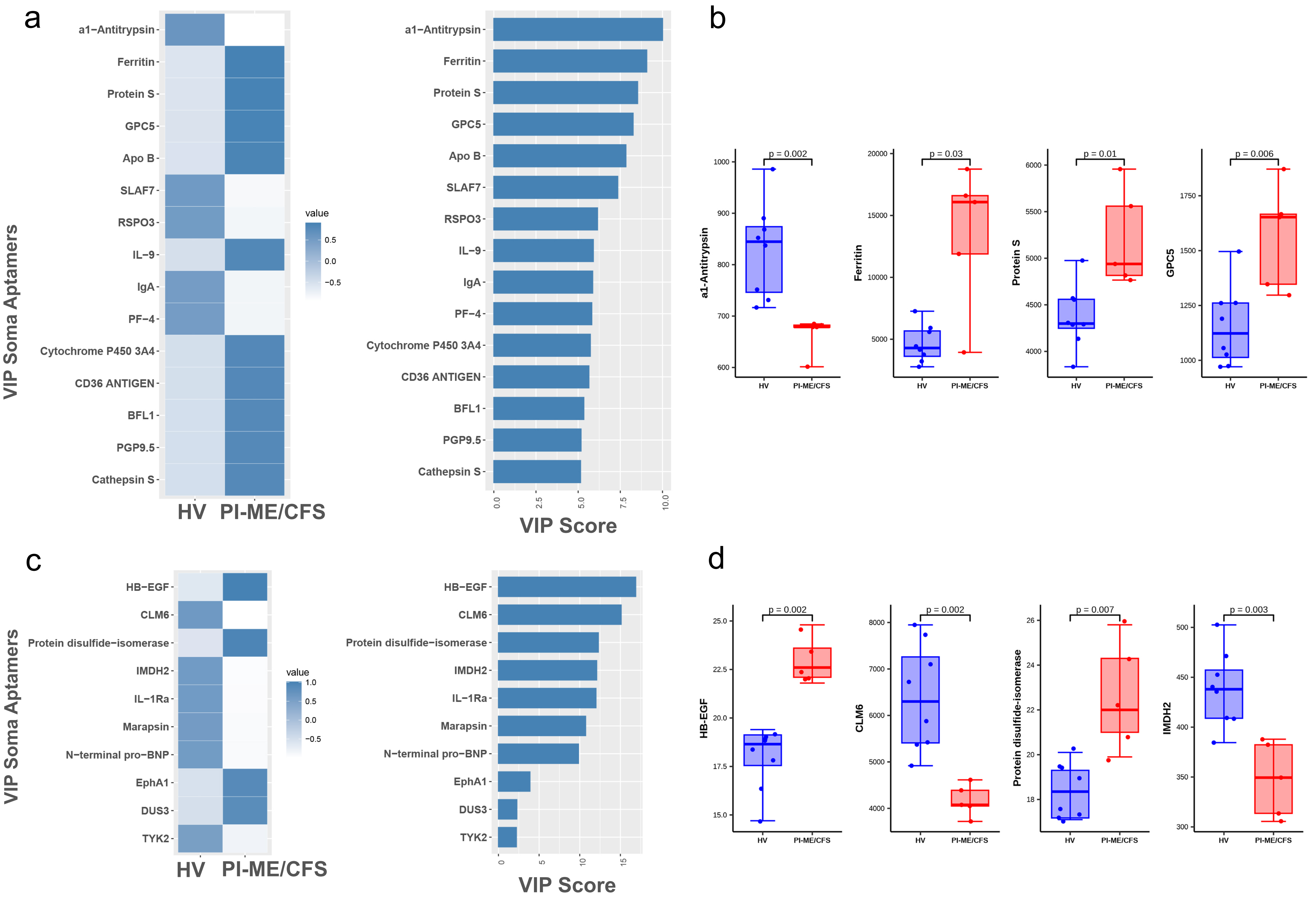
a
b
c
d
VIP Soma Aptamers
VIP Score
HV
PI-ME/CFS
value
a1−Antitrypsin
Ferritin
Protein S
GPC5
Apo B
SLAF7
RSPO3
IL−9
IgA
PF−4
Cytochrome P450 3A4
CD36 ANTIGEN
BFL1
PGP9.5
Cathepsin S
p = 0.002
p = 0.03
p = 0.01
p = 0.006
HB−EGF
CLM6
Protein disulfide−isomerase
IMDH2
IL−1Ra
Marapsin
N−terminal pro−BNP
EphA1
DUS3
TYK2
p = 0.002
p = 0.002
p = 0.007
p = 0.003

Figure S15: Multivariate and univariate analysis of the soma protein aptamers measured in the serum and cerebrospinal fluid (CSF) samples of male HV and PI-ME/CFS cohorts.
A Heatmaps of the average scaled expression values for the top15 indicated serum soma protein aptamers on y axis and the variable importance in prediction (VIP) scores of soma protein aptamers in the male cohort. B Expression box plots of the top 4 VIP protein aptamers for male cohort [HV (blue; n = 8 independent male participants) and PI-ME/CFS (red; n = 6 independent male participants)] in serum. C. The heatmap of the average scaled expression values for the top15 indicated cerebrospinal fluid soma protein aptamers on y axis and the variable importance in prediction (VIP) scores of indicated soma protein aptamers in the male cohort. D. Expression box plots of the top 4 VIP protein aptamers for male cohort [HV (blue; n = 8 independent male participants) and PI-ME/CFS (red; n = 6 independent male participants)] in cerebrospinal fluid. For box plots b and d, boxes depict the median (horizontal line) within quartiles 1–3 (bounds of box). Whiskers extend to minimum and maximum values. Exact p-values are from unadjusted Mann-Whitney U tests. No aptamer was significant after FDR-correction. Source data are provided as a Source Data file.

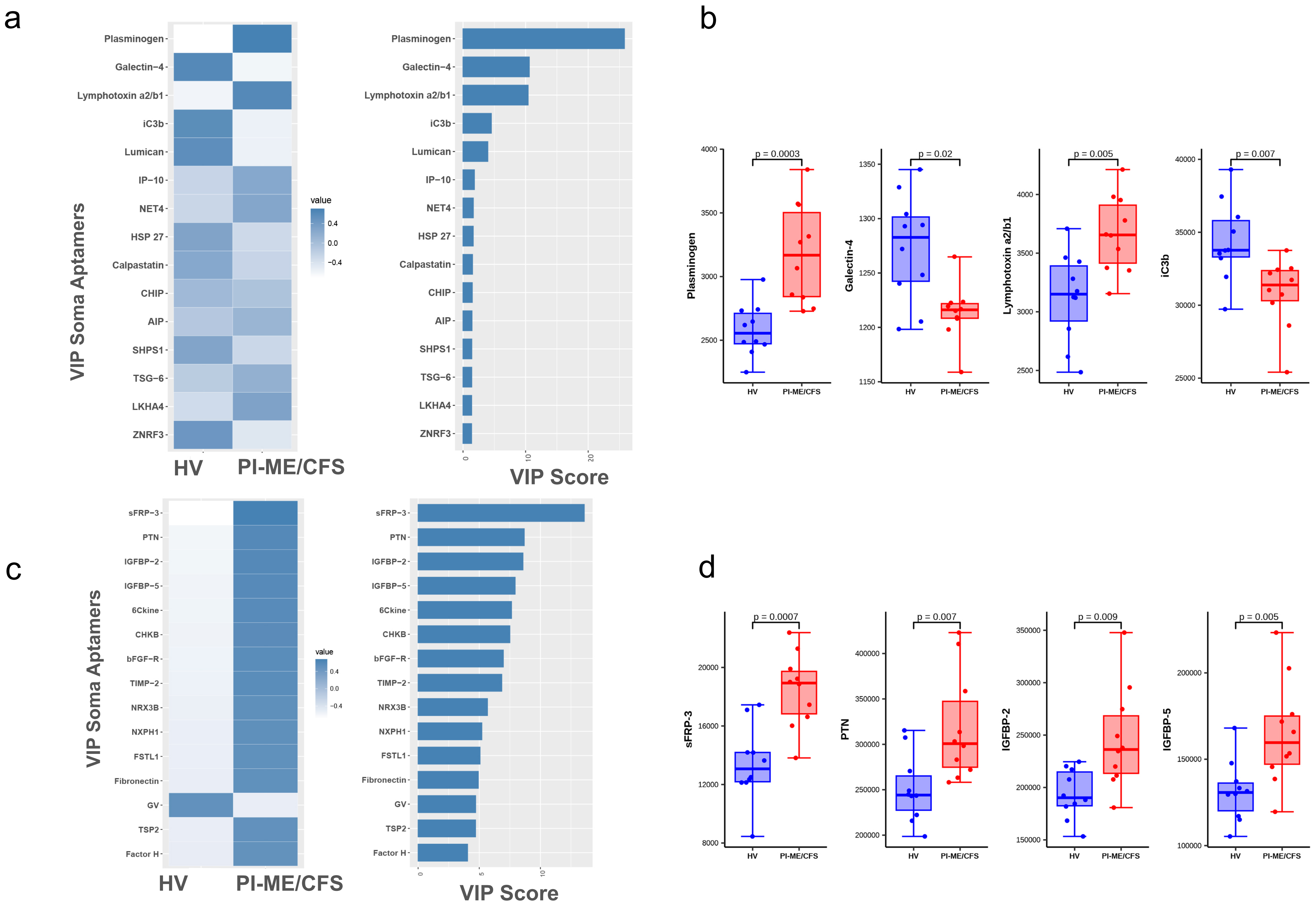

a
b
c
d
VIP Soma Aptamers
Plasminogen
Galectin−4
Lymphotoxin a2/b1
iC3b
Lumican
IP−10
NET4
HSP 27
Calpastatin
CHIP
AIP
SHPS1
TSG−6
LKHA4
ZNRF3
value
HV
PI-ME/CFS
VIP Score
p = 0.0003
p = 0.02
p = 0.005
p = 0.007
sFRP−3
PTN
IGFBP−2
IGFBP−5
6Ckine
CHKB
bFGF−R
TIMP−2
NRX3B
NXPH1
FSTL1
Fibronectin
GV
TSP2
Factor H
p = 0.0007
p = 0.007
p = 0.009
p = 0.005

Figure S16: Multivariate and univariate analysis of the soma protein aptamers measured in the serum and cerebrospinal fluid (CSF) samples of female HV and PI-ME/CFS cohorts.
A. Heatmaps of the average scaled expression values for the top15 indicated serum soma protein aptamers on y axis and the variable importance in prediction (VIP) scores of soma protein aptamers in the female cohort. B. Expression box plots of the top 4 VIP protein aptamers for female cohort HV [(blue; n = 10 independent female participants) and PI-ME/CFS (red; n = 9 independent female participants)]. C. The heatmap of the average scaled expression values for the top15 indicated cerebrospinal fluid soma protein aptamers on y axis and the variable importance in prediction (VIP) scores of indicated soma protein aptamers in the female cohort. D. Expression box plots of the top 4 VIP protein aptamers for the female cohort [(H; HV (blue; n = 10 independent female participants) and PI-ME/CFS (red; n = 9 independent female participants)]. For box plots b and d, boxes depict the median (horizontal line) within quartiles 1–3 (bounds of box). Whiskers extend to minimum and maximum values. Exact p-values are from unadjusted Mann-Whitney U tests. No aptamer was significant after FDR-correction. Source data are provided as a Source Data file.

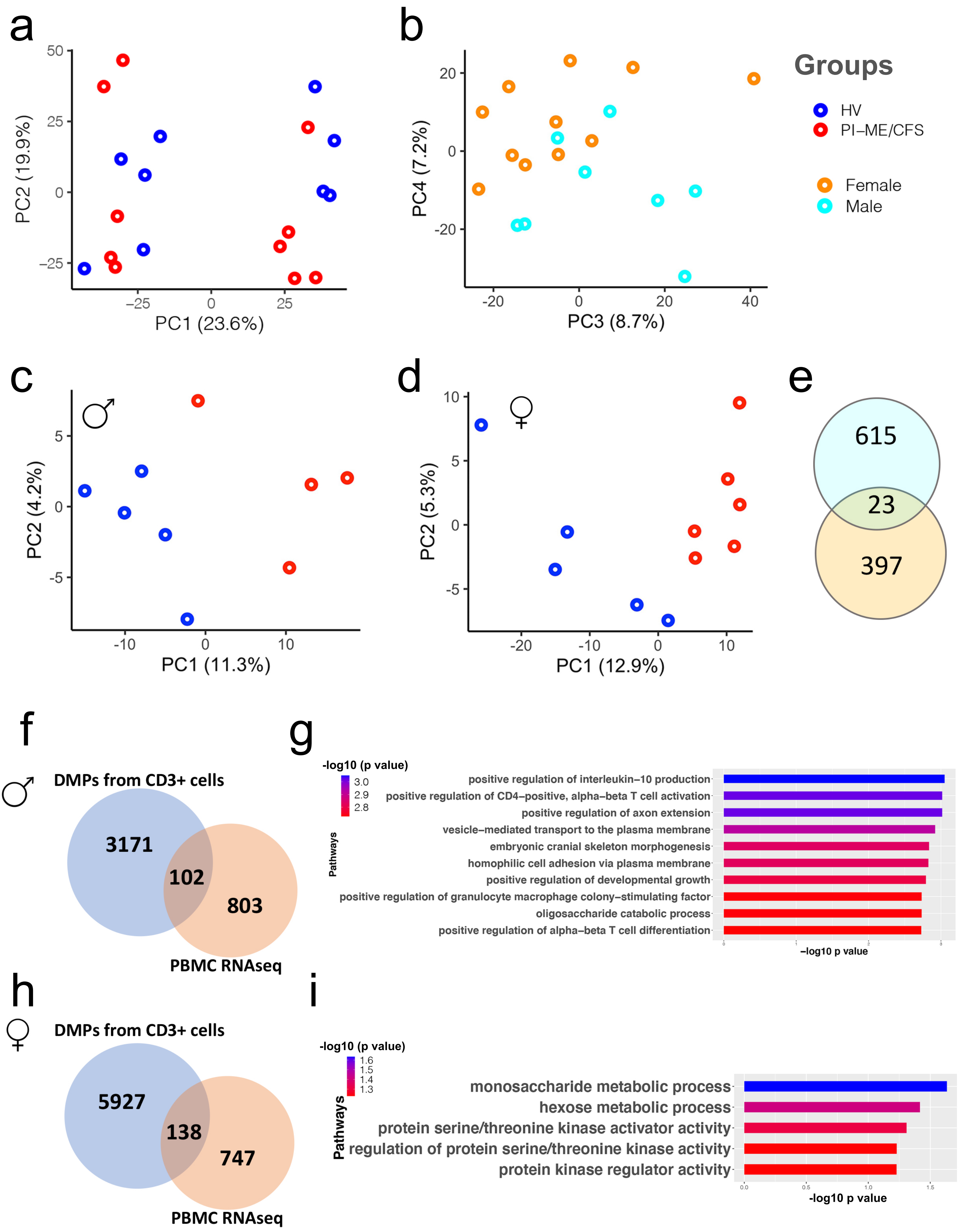
a
PC2 (19.9%)
PC1 (23.6%)
b
PC4 (7.2%)
PC3 (8.7%)
Groups
HV
PI–ME/CFS
Female
Male
c
PC2 (4.2%)
PC1 (11.3%)
d
PC2 (5.3%)
PC1 (12.9%)
e
615
23
397
f
DMPs from CD3+ cells
3171
102
803
PBMC RNAseq
g
-log10 (p value)
Pathways
positive regulation of interleukin−10 production
positive regulation of CD4−positive, alpha−beta T cell activation
positive regulation of axon extension
vesicle−mediated transport to the plasma membrane
embryonic cranial skeleton morphogenesis
homophilic cell adhesion via plasma membrane
positive regulation of developmental growth
positive regulation of granulocyte macrophage colony−stimulating factor
oligosaccharide catabolic process
positive regulation of alpha−beta T cell differentiation
−log10 p value
h
DMPs from CD3+ cells
5927
138
747
PBMC RNAseq
i
-log10 (p value)
Pathways
monosaccharide metabolic process
hexose metabolic process
protein serine/threonine kinase activator activity
regulation of protein serine/threonine kinase activity
protein kinase regulator activity
-log10 p value

Figure S17: Analysis of external chronic fatigue syndrome RNAseq and methylation datasets showed distinct subset of genes are perturbed in male and female cohorts.

A-B. PCA computed from all gene expression values measured in the monocytes with indicated groups: HV (blue; n = 9 independent participants) and PI-ME/CFS (red; n = 10 independent participants) or males (turquoise; n = 8 independent participants) and females (orange; n = 11 independent participants) highlighted for the indicated PCs. C-D. PCA computed from DE gene expression values in (C) male (HV blue; n = 5 independent participants and PI-ME/CFS (red; n = 4 independent participants) and (D) female cohorts (HV blue; n = 5 independent participants and PI-ME/CFS (red; n = 6 independent participants. E. Venn diagram showing common DE genes identified from male and female cohorts (DE genes are genes with p value <0.05). F. Venn diagram showing overlap between differentially methylated genes identified in CD3+ cells of female CFS cohorts that were identified as DE genes from the PBMC samples of male cohorts analyzed in this study. G. Pathway enrichment analysis of genes commonly perturbed in the methylation profiling and gene expression profiling in male cohorts. H. Venn diagram showing overlap between differentially methylated genes identified in CD3+ cells of female CFS cohorts that were also DE genes from the PBMC samples analyzed in the female cohorts of this study. I. Pathway enrichment analysis of genes commonly perturbed in the methylation profiling and gene expression profiling in female cohort. DE: differentially expressed; PBMC: peripheral blood mononuclear cells; PC: principal component. Source data are provided as a Source Data file.

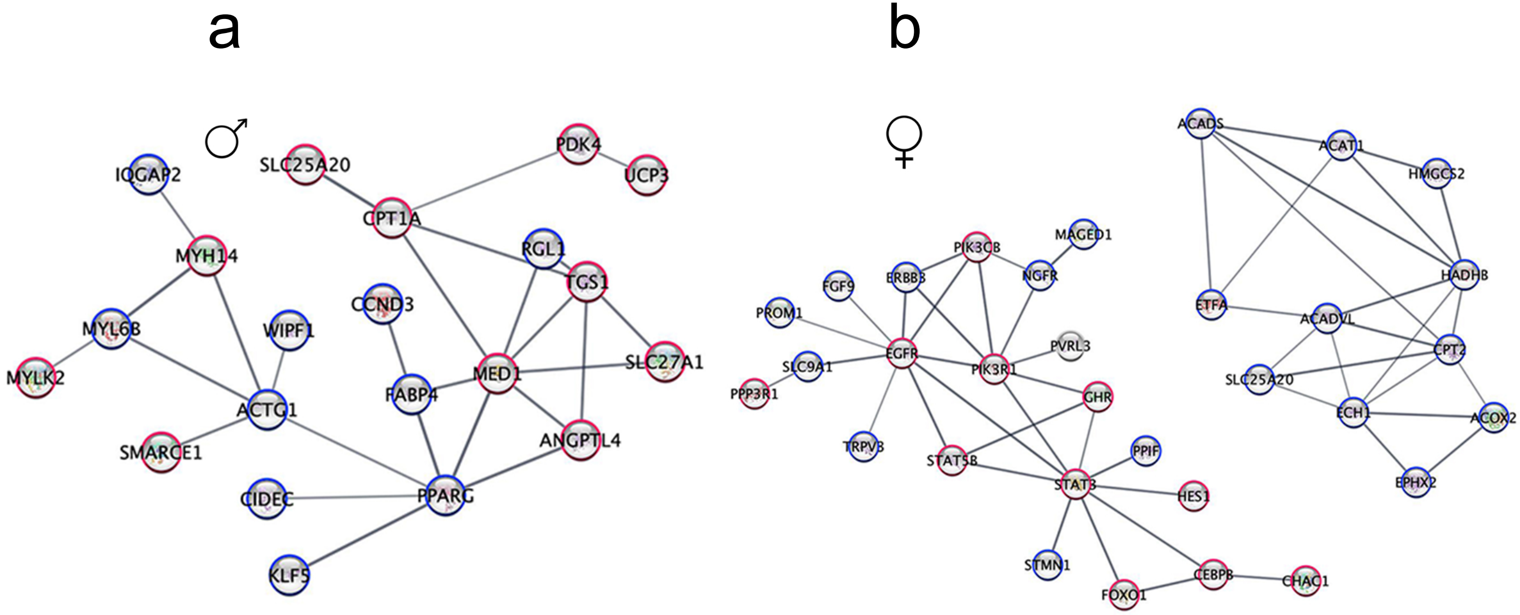

a
♂
SLC25A20
PDK4
UCP3
IQGAP2
CPT1A
MYH14
RGL1
TGS1
CCND3
MYL6B
WIPF1
MED1
SLC27A1
MYLK2
FABP4
ACTG1
ANGPTL4
SMARCE1
CIDEC
PPARG
KLF5
b
♀
ACADS
ACAT1
HMGCS2
MAGED1
PIK3CB
ERBB3
NGFR
FGF9
HADHB
PROM1
ETFA
ACADVL
PVRL3
EGFR
PIK3R1
SLC9A1
CPT2
SLC25A20
PPP3R1
GHR
ECH1
ACOX2
TRPV3
STAT5B
PPIF
STAT3
HES1
EPHX2
STMN1
CEBPB
CHAC1
FOXO1

Figure S18: Male and female cohorts have distinct differential gene expression profiles in the muscle: A-B. Protein-Protein interactome of a subset of DE genes from (A) male and (B) female cohorts and the fold change information are overlaid on the nodes. Red color indicates upregulation and blue color indicated downregulation in PI-ME/CFS group compared to healthy group. Source data are provided as a Source Data file.

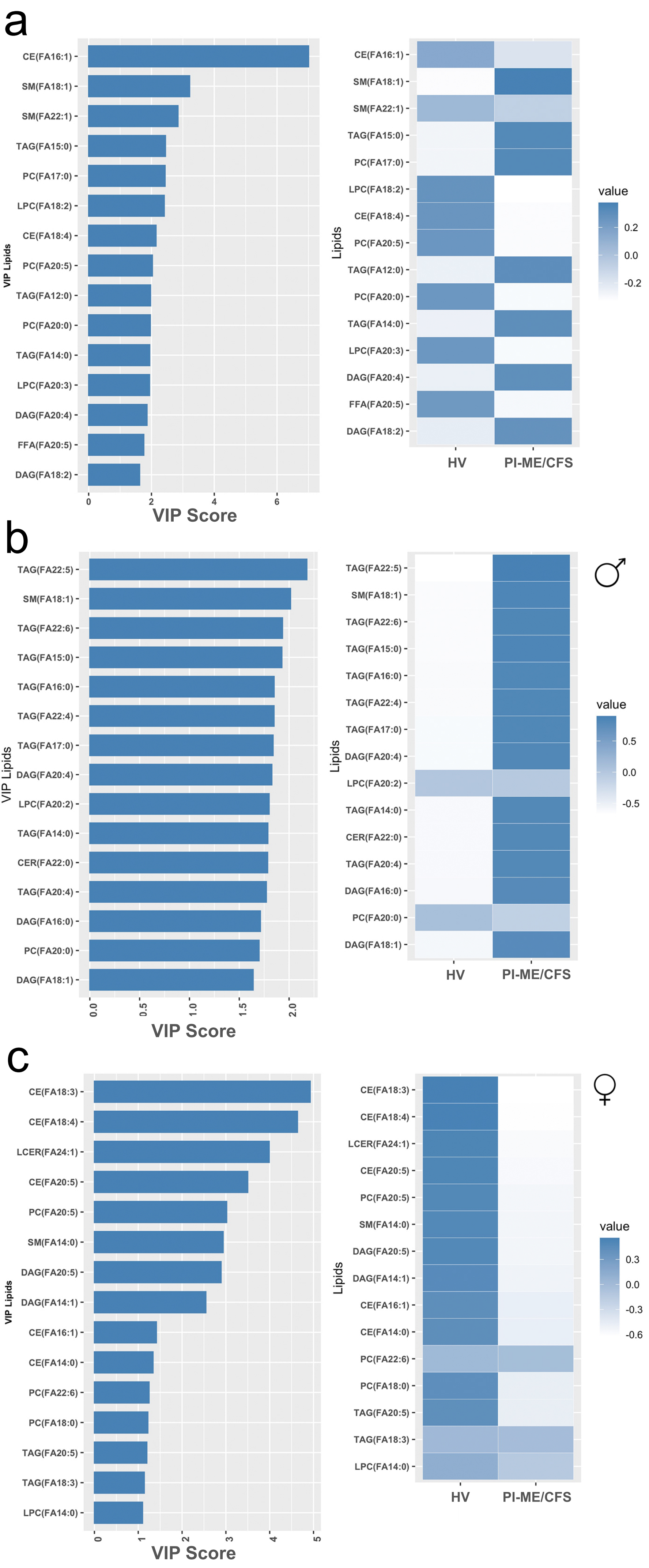

a
CE(FA16:1)
SM(FA18:1)
SM(FA22:1)
TAG(FA15:0)
PC(FA17:0)
LPC(FA18:2)
CE(FA18:4)
PC(FA20:5)
TAG(FA12:0)
PC(FA20:0)
TAG(FA14:0)
LPC(FA20:3)
DAG(FA20:4)
FFA(FA20:5)
DAG(FA18:2)
VIP Lipids
VIP Score
Lipids
HV
PI-ME/CFS
value
b
TAG(FA22:5)
SM(FA18:1)
TAG(FA22:6)
TAG(FA15:0)
TAG(FA16:0)
TAG(FA22:4)
TAG(FA17:0)
DAG(FA20:4)
LPC(FA20:2)
TAG(FA14:0)
CER(FA22:0)
TAG(FA20:4)
DAG(FA16:0)
PC(FA20:0)
DAG(FA18:1)
c
CE(FA18:3)
CE(FA18:4)
LCER(FA24:1)
CE(FA20:5)
PC(FA20:5)
SM(FA14:0)
DAG(FA20:5)
DAG(FA14:1)
CE(FA16:1)
CE(FA14:0)
PC(FA22:6)
PC(FA18:0)
TAG(FA20:5)
TAG(FA18:3)
LPC(FA14:0)

Figure S19: Multivariate analysis of serum lipidomics data:
A. Variable importance in prediction (VIP) scores of lipids with VIP score >1 and heatmap of the average scaled expression values of the indicated VIP metabolites in all samples. B. Variable importance in prediction (VIP) scores of lipids with VIP score >1 and heatmap of the average scaled expression values of the indicated VIP metabolites in the male cohort. C. Variable importance in prediction (VIP) scores of lipids with VIP score >1 and heatmap of the average scaled expression values of the indicated VIP metabolites in the female cohort. Source data are provided as a Source Data file.

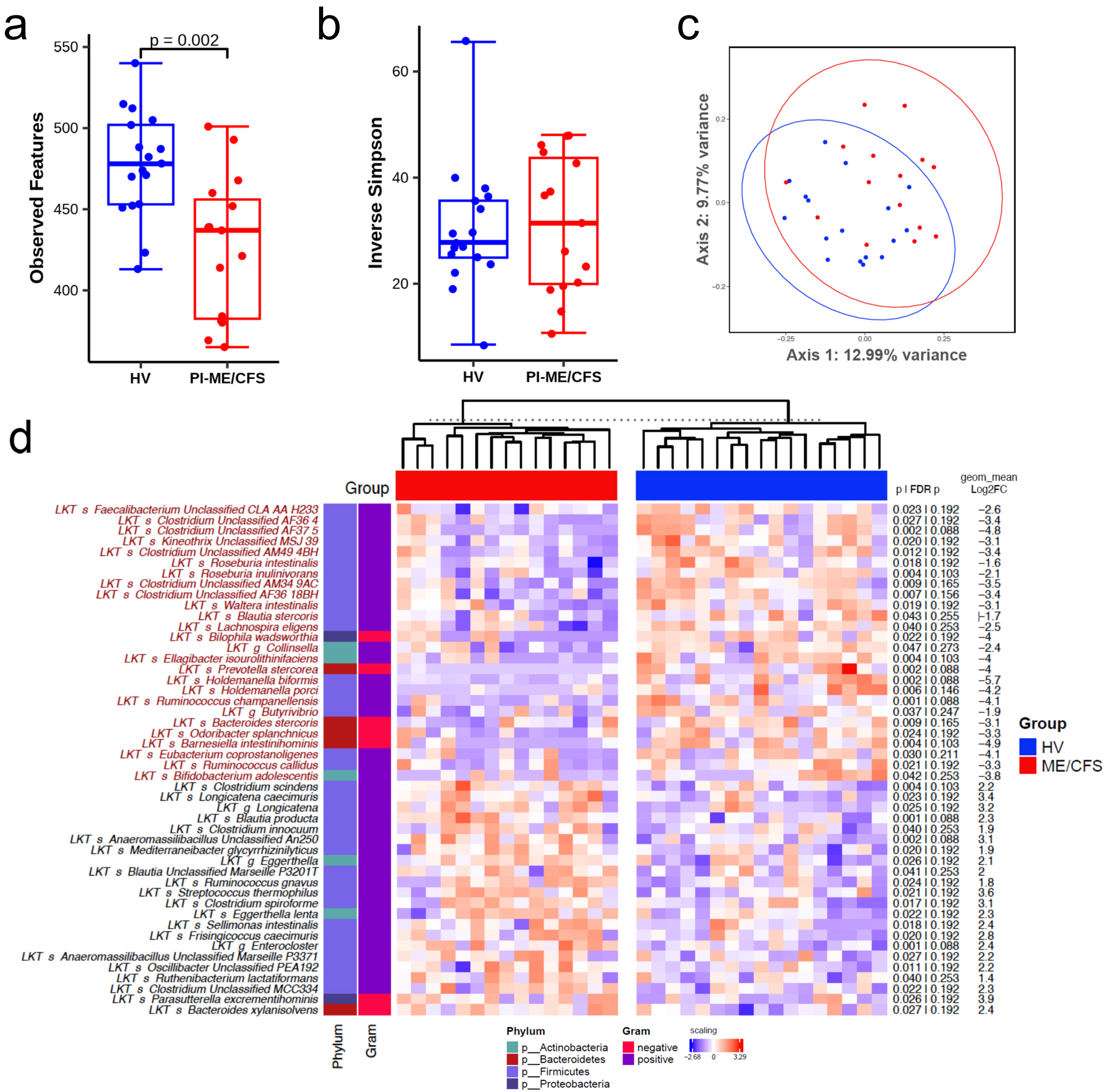

a
p = 0.002
Observed Features
550
500
450
400
HV
PI-ME/CFS
b
Inverse Simpson
60
40
20
HV
PI-ME/CFS
c
Axis 2: 9.77% variance
0.2
0.0
−0.2
−0.25
0.00
0.25
Axis 1: 12.99% variance
d
Group
p | FDR p
geom_mean Log2FC
LKT s Faecalibacterium Unclassified CLA AA H233 0.023 | 0.192 −2.6
LKT s Clostridium Unclassified AF36 4 0.027 | 0.192 −3.4
LKT s Clostridium Unclassified AF37 5 0.002 | 0.088 −4.8
LKT s Kineothrix Unclassified MSJ 39 0.020 | 0.192 −3.1
LKT s Clostridium Unclassified AM49 4BH 0.012 | 0.192 −3.4
LKT s Roseburia intestinalis 0.018 | 0.192 −1.6
LKT s Roseburia inulinivorans 0.004 | 0.103 −2.1
LKT s Clostridium Unclassified AM34 9AC 0.009 | 0.165 −3.5
LKT s Clostridium Unclassified AF36 18BH 0.007 | 0.156 −3.4
LKT s Waltera intestinalis 0.019 | 0.192 −3.1
LKT s Blautia stercoris 0.043 | 0.255 −1.7
LKT s Lachnospira eligens 0.040 | 0.253 −2.5
LKT s Bilophila wadsworthia 0.022 | 0.192 −4
LKT g Collinsella 0.047 | 0.273 −2.4
LKT s Ellagibacter isourolithinifaciens 0.004 | 0.103 −4
LKT s Prevotella stercorea 0.002 | 0.088 −4
LKT s Holdemanella biformis 0.002 | 0.088 −5.7
LKT s Holdemanella porci 0.006 | 0.146 −4.2
LKT s Ruminococcus champanellensis 0.001 | 0.088 −4.1
LKT g Butyrivibrio 0.037 | 0.247 −1.9
LKT s Bacteroides stercoris 0.009 | 0.165 −3.1
LKT s Odoribacter splanchnicus 0.024 | 0.192 −3.3
LKT s Barnesiella intestinihominis 0.004 | 0.103 −4.9
LKT s Eubacterium coprostanoligenes 0.030 | 0.211 −4.1
LKT s Ruminococcus callidus 0.021 | 0.192 −3.3
LKT s Bifidobacterium adolescentis 0.042 | 0.253 −3.8
LKT s Clostridium scindens 0.004 | 0.103 2.2
LKT s Longicatena caecimuris 0.023 | 0.192 3.4
LKT g Longicatena 0.025 | 0.192 3.2
LKT s Blautia producta 0.001 | 0.088 2.3
LKT s Clostridium innocuum 0.040 | 0.253 1.9
LKT s Anaeromassilibacillus Unclassified An250 0.002 | 0.088 3.1
LKT s Mediterraneibacter glycyrrhizinilyticus 0.020 | 0.192 1.9
LKT g Eggerthella 0.026 | 0.192 2.1
LKT s Blautia Unclassified Marseille P3201T 0.041 | 0.253 2
LKT s Ruminococcus gnavus 0.024 | 0.192 1.8
LKT s Streptococcus thermophilus 0.021 | 0.192 3.6
LKT s Clostridium spiroforme 0.017 | 0.192 3.1
LKT s Eggerthella lenta 0.022 | 0.192 2.3
LKT s Sellimonas intestinalis 0.018 | 0.192 2.4
LKT s Frisingicoccus caecimuris 0.020 | 0.192 2.8
LKT g Enterocloster 0.001 | 0.088 2.4
LKT s Anaeromassilibacillus Unclassified Marseille P3371 0.027 | 0.192 2.2
LKT s Oscillibacter Unclassified PEA192 0.011 | 0.192 2.2
LKT s Ruthenibacterium lactatiformans 0.040 | 0.253 1.4
LKT s Clostridium Unclassified MCC334 0.022 | 0.192 2.3
LKT s Parasutterella excrementihominis 0.026 | 0.192 3.9
LKT s Bacteroides xylanisolvens 0.027 | 0.192 2.4
Phylum
Gram
Group
HV
ME/CFS
Phylum
p__Actinobacteria
p__Bacteroidetes
p__Firmicutes
p__Proteobacteria
Gram
negative
positive
scaling
−2.68
0
3.29

Figure S20: Measurements of microbial composition using shotgun metagenomics:
A-B. Boxplots of the Alpha diversity of stool samples as measured by (A) number of observed species-level taxa between the HV (blue; n = 17 independent participants) and PI-ME/CFS (red; n = 15 independent participants) groups using a unadjusted two-sided t-test for independent samples with equal variance (p = 0.002) and (B) Inverse Simpson Index. For box plots a-b boxes depict the median (horizontal line) within quartiles 1–3 (bounds of box). Whiskers extend to minimum and maximum values. C. Beta diversity of stool samples at the species-level as shown by Principal Coordinate Analysis (PCoA) of Bray-Curtis dissimilarity. D. Scaled relative abundance heatmap of the species-level taxa responsible for the differences in diversity between the HV (blue; n = 18 independent participants) and PI-ME/CFS (red; n = 15 independent participants) groups. P-values were calculated using the Mann-Whitney U test. Adjusted p-values were obtained by False Discovery Rate (FDR) adjustment. Log2 fold changes were calculated using the geometric mean relative abundance of each feature in each group. Only features with a raw p-value ≤ 0.05 are shown, and with a minimum log2 foldchange of 1. Source data are provided as a Source Data file.

SUPPLEMENTARY RESULTS

Stringent Study Recruitment Process identified Individuals with post-infectious Myalgic encephalomyelitis/chronic fatigue syndrome (PI-ME/CFS)

A stringent recruitment process reduced PI-ME/CFS case heterogeneity. Study recruitment took place between December 2016 and February 2020 (Figure 1A). Inclusion and exclusion criteria are listed in Data S1. A total of 484 inquiries for participation were made. The majority of these were self-referrals. Of these, 267 persons were screened out during the initial telephone screening interview (Data S2A). A total of 217 persons underwent a medical record review and a telephone interview by a study physician. Of those, 146 were excluded after review (Data S2B) and 44 persons did not complete the review process prior to closing recruitment due to the pandemic.

A total of 27 PI-ME/CFS participants underwent research evaluation at the National Institutes of Health (NIH) Clinical Center. Two participants withdrew after providing informed consent and completing a history and physical exam without undergoing any research investigations. Of the remaining 25 PI-ME/CFS participants, four were found to have previously undiagnosed medical conditions that were related to ME/CFS symptoms. This included one case each of desmoplastic small round cell tumor, atypical inflammatory myositis, primary biliary cholangitis, and parkinsonism. Of the remaining 21 PI-ME/CFS participants, four participants were determined not to have a demonstrable infectious association to their ME/CFS symptoms during the case adjudication process (Data S2C). A total of 17 adjudicated PI-ME/CFS participants completed the initial deep phenotyping measurements; eight of these participants also completed the exercise stress measurements.

PI-ME/CFS participants had a variety of well documented infections that precipitated the illness

These included 10 cases of mixed upper respiratory tract infections (eight pharyngitis, two sinusitis, two bronchitis and two otitis), three cases of acute Epstein-Barr Virus infection, one case of gastroenteritis, one case of atypical hepatitis, one case of herpes zoster ophthalmicus, and one case of Ramsay Hunt syndrome due to herpes zoster. The time lapse between infection onset and research evaluation ranged between 13 to 59 months (33 ± 15 months).

All individuals met criteria used for diagnosis of ME/CFS

All PI-ME/CFS participants met at least one published ME/CFS criterion. 14 met the 1994 Fukuda Criteria, nine met the 2003 Canadian Consensus Criteria, and all 17 met the 2015 Institute of Medicine Criteria (Data S2D).

Healthy Volunteers were thoroughly screened for underlying illnesses

A total of 25 healthy volunteers (HVs) were recruited to serve as comparator participants. Of these, one participant withdrew prior to completing deep phenotyping measurements. Three participants were excluded after evaluation related to asymptomatic medical conditions that were identified. One had leukodystrophy, another had pleocytosis in the cerebrospinal fluid, and the third had early onset dementia. A total of 21 healthy volunteer comparators completed

the initial deep phenotyping measurements; nine of these comparators also completed the exercise stress measurements.

Healthy Volunteers were carefully matched to the PI-ME/CFS individuals

Recruited PI-ME/CFS cases and HV comparators were matched on demographic characteristics. The demographic characteristics of the HV and PI-ME/CFS participants are detailed in Figures 1B-C and Data S5. Participants were matched with regards to age (42 ± 13 versus 38 ± 15 years), body mass index (25.8 ± 3.4 versus 25.9 ± 5.3 kg/m^2) and sex (10 versus 6 men; 12 versus 11 women). The HV group consisted of three multiracial participants, of which one was Hispanic, and 18 White participants. The PI-ME/CFS group consisted of two Asians and 15 White participants, of which two were Hispanic. All participants except for one HV had a high school diploma or higher education. Thirteen PI-ME/CFS participants were on long-term disability while none of the HVs were disabled.

Characteristics of Individuals who Underwent a Second Study Visit

All participants were invited to return for a second study visit to measure exercise response. Eight PI-ME/CFS and nine HVs were able to return for participation prior to the study being closed due to the SARS-CoV-2 pandemic. The PI-ME/CFS participants (age: 42±13 years, female: 50%, BMI: 26.7±5.0) were older and more often male than those that could not participate (age: 34.5±13.8 years, female %: 78%, BMI: 24.8±5.4).

The Participants did not have a Significant History of Use of Drugs of Abuse

None of the PI-ME/CFS participants ever used tobacco products while three HVs were former tobacco users and two occasionally used tobacco products socially. Cannabis products were used occasionally by one PI-ME/CFS and one HV. None of the participants were using or abusing illicit drugs or alcohol at the time of evaluation.

The Participants had Valid Performances on Neurocognitive Testing

The Word Memory Test (WMT), the B Test, and the Dot Test were administered as part of the neuropsychological testing battery (Data S6). Of the 18 HVs tested, two failed a validity test, none failed more than one validity test, and none failed the WMT. Of the 15 PI-ME/CFS participants tested, one failed a validity test, none failed more than one validity test, and none failed either the WMT or B test. No participants had an invalid performance profile on the Test of Variables of Attention (TOVA). Thus, PI-ME/CFS and HV participants had equally valid performances on neurocognitive testing.

Physical Symptoms were common in PI-ME/CFS

PI-ME/CFS participants reported magnitudes of physical symptoms more than HVs (Figure 1C; Data S7). This was seen with the SF-36 Physical Component Score (PCS), which was more than 2.5 standard deviations lower in PI-ME/CFS participants compared to both the HVs and US population norms (56.7 ±3.1 versus 23.5 ±10.0, p=0.00000000007). Thus, PI-ME/CFS had more physical symptoms, in both number and severity, than HV participants.

Fatigue was a prominent complaint of PI-ME/CFS participants

Fatigue was similarly worse (on the magnitude of two to three standard deviations) for PI-ME/CFS participants on the Brief Fatigue Inventory (1.7±2.4 versus 16.9±4.9, p=0.0000003), PROMIS fatigue (56.5±8.7 versus 66.4±4.7, p=0.000002), and all Multidimensional Fatigue Inventory subscales (all p≤0.0001). The qualitative experience of this level of fatigue is evoked in this participant description: "…I call it Groundhog Day, except it's not a good day … You wake up feeling like you already ran a marathon. [That's] how I start every day. [So] every day is [like] how do I get through it? How do I do the basic things like go to the bathroom by myself or shower by myself. Or maybe this day I'm not going to shower because I just feel too terrible."

PI-ME/CFS Individuals had Multiple types of Pain

PI-ME/CFS participants reported more clinically substantial pain (Data S7) on PROMIS Pain Behavior (38.6±8.4 versus 53.3+9.8, p=0.00007), PROMIS Pain Interference (42.4±3.6 versus 58.9 ±11.3, p=0.00004), PROMIS Pain Intensity (39.7±5.7 versus 56.2±12.4, p=0.00005), Polysymptomatic Distress Scale (1.6±1.6 versus 13.2 ±7.2, p=0.0000002), the McGill Pain Questionnaire (1.0±3.5 versus 19.9±16.0, p=0.00003), and tender point counts (1± 1.6 versus 5.2±4.3, p=0.0002). Two PI-ME/CFS participants met 1990 American College of Rheumatology (ACR) fibromyalgia criteria and seven met modified 2010 ACR fibromyalgia criteria. The qualitative experience of PI-ME/CFS pain is evoked in this participant description: "I think the pain is what drives me batty because there's so many different kinds of pain. They fluctuate… The nerve pain [is] all over. [There is] Stomach pain. The headaches [are] a fact of life. Torso pain is kind of a spinoff of that, [and] muscle pain."

PI-ME/CFS participants complained of significantly more neuropathic symptoms than HVs, with significantly increased scores on the Neuropathic Pain Scale Overall Unpleasantness (0.6± 1.7 versus 5.1±3.4, p=0.000008), Deep Severity (0.5±1.6 versus 5.6±3.5, p=0.00001), and Surface Severity (0.2±0.7 versus 2.6±2.5, p=0.0001) scores (Data S7). The qualitative experience of this level of neuropathic sensations is evoked in this participant description: "I get cramps, and this burning, electrical, stinging sensation. It's not pleasant. [It] goes through my body in other forms but my legs are the worst."

PI-ME/CFS Individuals had Multiple types of Motor Symptoms

PI-ME/CFS participants frequently reported motor symptoms (14.3% versus 70.6%), including weakness (9.5% versus 82.4%), difficulty initiating movements (0% versus 29.4%), paralysis (0% versus 11.8%), tremors (9.5% versus 23.5%), dystonia (4.7% versus 23.5%), dysarthria (0% versus 23.5%) and gait/balance problems (4.7% versus 41.2%). Weakness severity, as measured by weakness Visual Analogue Scale (VAS), was greater in PI-ME/CFS participants (3.9 ± 6.8 versus 27.3± 26.7, p=0.02). The qualitative of experiences of weakness is evoked in this participant description: "It's muscle pain and weakness. [After] each setback… my muscles get wimpier and wimpier. [If] I'm totally relaxed, it feels like my muscles are pureed. [If] I try to do anything, they cramp up like you've been gripping something too long...". Other types of motor symptoms are also described: "The shaking is annoying… When I feel that first twinge [of back pain], I lie down… and I can stop [what is coming] which will be dragging feet, my head hanging forward, the shakes, and difficulty in projecting my voice."

General Physical Examination was unrevealing in PI-ME/CFS

There were few clinically substantial findings on physical examination for both PI-ME/CFS and HVs. Two PI-ME/CFS participants and none of the HVs met Beighton criteria for hypermobility. One PI-ME/CFS participant had a myofascial pain disorder of the rectus abdominus muscles. One PI-ME/CFS participant had tinea corporis that resolved with therapy. No preauricular, anterior cervical, posterior cervical, supraclavicular, submandibular, axillary, epitrochlear, inguinal, or popliteal lymphadenopathy was noted in any of the participants.

Neurological Examination was unrevealing in PI-ME/CFS

Three PI-ME/CFS participants had abnormalities on neurological examination. One PI-ME/CFS participant had findings consistent with a small fiber peripheral neuropathy confirmed by a QSART test (however, intraepidermal nerve fiber and sweat gland nerve fiber density measures were normal). Another had residual facial weakness from Ramsay-Hunt syndrome. A third had a clinical history suggestive of an untreated migraine disorder. All HVs had normal neurological examinations.

Magnetic Resonance Imaging of Brain showed Non-Specific Findings

There were few abnormalities noted on brain magnetic resonance imaging (MRI). No structural abnormalities were noted except for incidental findings of non-specific punctate white matter lesions in five HV and five PI-ME/CFS participants. Three HVs were noted to have mild cerebral atrophy and one HV had an asymptomatic displaced disc at C2-C3. One PI-ME/CFS participant did not undergo MRI due to presence of an intrauterine device.

Nerve Fiber Densities in Skin were Normal

A subset of HV (n=9) and PI-ME/CFS participants (n=11) underwent skin biopsies for assessment of epidermal and sweat gland nerve fiber densities. No group differences were noted in intraepithelial nerve fiber density of the distal leg (Figure S1A) or distal thigh (Figure S1B). No group differences were noted in sweat gland nerve fiber density of the distal leg (Figure S1C) or distal thigh (Figure S1D). No PI-ME/CFS participants were below the normative values of small fiber density for age and gender[1].

Biomarkers of Neuronal Injury were Not Elevated in PI-ME-CFS

Plasma and cerebrospinal fluid samples were measured for markers of neuronal injury. No group differences were noted in plasma for tau (Figure S1E), neurofilament light chain (NfL; Figure S1F), or glial fibrillary acidic protein (GFAP; Figure S1G). Plasma ubiquitin carboxyl-terminal esterase L1 (UCHL1) are not reported as 38% of the cohort had CV values greater than 20%. No group differences were noted in cerebrospinal fluid for tau (Figure S1H), NfL (Figure S1I), GFAP (Figure S1J) or UCHL1 (Figure S1K).

Detailed Clinical Laboratory Evaluation was Unrevealing in PI-ME/CFS

There were few clinically relevant findings on clinical laboratory evaluation. A large panel of parameters were tested in blood and urine that included hematology, chemistry, endocrinology, immunology, virology, and heavy metal panels (Data S8, S9). There were no group differences in any laboratory parameter except for Mean Corpuscular Volume (MCV: 90.0

± 3.6 versus 87.8 ± 2.7 fL, p=0.04) and Mean Corpuscular Hemoglobin (MCH: 30.2 ± 1.0 versus 29.3 ± 1.5 pg, p=0.05). No participants had low levels of iron, transferrin, or transferrin saturation. Three PI-ME/CFS participants had low and seven had high ferritin levels. No group differences were observed in iron levels in cerebrospinal fluid by inductively coupled plasma-mass spectroscopy (46404.4 ±19425.0 versus 48700.1 ±21886.1 parts per trillion, p=0.7).

No individual participants had clinical abnormalities in blood chemistries, blood cell counts, creatinine clearance, or glucose metabolism. Fasting lipid measures were elevated in six HV and seven PI-ME/CFS participants. No participants had Vitamin B12 or D deficiency and two participants from each group had low folate levels. No participants had evidence of active Epstein-Barr Virus (EBV) or Cytomegalovirus (PCR), Lyme disease (C6 peptide), syphilis (Enzyme immune assay), Hepatitis B or C, or HIV (antibody testing). All participants had IgG antibodies to EBV capsid antigen. All participants were negative for IgM antibodies to EBV capsid antigen except for one PI-ME/CFS participant with an equivocal result. All participants had EBV nuclear antigen antibodies except for one HV. No PI-ME/CFS participants had overt evidence of mast cell disease as measured by total serum tryptase. No participants had evidence of heavy metal toxicity as measured by 24-hour urine assay.

There were few laboratory abnormalities noted in the participants. One PI-ME/CFS participant was found to have asymptomatic duloxetine-induced hepatotoxicity that normalized with treatment cessation. One HV had mildly abnormal liver function tests related to non-alcoholic fatty liver disease. Four PI-ME/CFS participants had a positive ANA without other signs of autoimmune or rheumatic disease compared to one HV. Two PI-ME/CFS participants had thyroid autoantibodies (one thyroid peroxidase and one anti-thyroglobulin antibody) in the setting of normal thyroid stimulating hormone (TSH) and triiodothyronine levels. Two PI-ME/CFS participants had mild normocytic anemia, one in the setting of elevated inflammatory parameters (CRP 7.9, ESR 48, normal ferritin, normal platelets) and the other in the setting of subclinical autoimmunity (Positive ANA and thyroid peroxidase, low ferritin, normal CRP and ESR). One PI-ME/CFS participant had asymptomatic leukopenia. One PI-ME/CFS participant had a positive urine test for non-toxic arsenobetaine. One HV had urine leukocytosis of unclear origin.

Cerebrospinal fluid cell counts, protein, glucose and IgG index were normal in both groups (Data S8). One healthy participant had *pattern 3* oligoclonal bands. Two PI-ME/CFS participants had *pattern 2* (both of the normocytic anemia participants) and two had *pattern 4* oligoclonal bands (one participant with duloxetine-induced hepatotoxicity).

Mood Disorders were Present in PI-ME-CFS

PI-ME/CFS participants were more often depressed and anxious than HV participants but not severely so. Psychological impact on function was measured using the SF-36 Mental Component Score (MCS). Although the PI-ME/CFS group had lower MCS scores (54.8 ± 3.7 versus 49.1 ± 6.8, p=0.003), they were equal to the average population norm.

Using the Beck Depression Inventory-II, five PI-ME/CFS participants had mild depression and one participant had moderate depression. Using the Beck Anxiety Inventory, three PI-ME/CFS participants had mild and three had moderate anxiety. PI-ME/CFS participants had significantly more self-reported depressive (2.1 ± 3.4 versus 12.0 ± 4.6, p=0.000002) and anxiety (1.9 ± 2.0 versus 7.8 ± 7.4, p=0.007) symptoms.

Somatization was evaluated with the Patient Health Questionnaire 15 (PHQ-15), with markedly increased scores in PI-ME/CFS participants (2.0 ± 2.6 versus 11.6 ± 4.6, p< 0.0001). Fatigue catastrophizing was greater in PI-ME/CFS participants (13.2 ± 5.8 versus 21.1 ± 6.9, p<0.0001).

Participants were also evaluated with the Structured Clinical Interview – DSM 5 (SCID-5). Three PI-ME/CFS participants had past depression predating the development of PI-ME/CFS, none had past anxiety, two had current depression, one had current anxiety, and two had subclinical anxiety. In comparison, three HVs had past depression, none had current depression, none had past anxiety, two had current anxiety, and two had subclinical anxiety. The lifetime exposure to trauma was similar in the groups (76.2% versus 70.6%, p=0.7). One participant in each group met diagnostic criteria for Post-Traumatic Stress Disorder.

PI-ME/CFS participants were also asked to describe their emotional states. Some descriptions, such as "I was like suddenly bed bound after being really active... I became so depressed that I couldn't do everything that I wanted to do. [For a while] I just felt like giving up, like I wasn't going to get better ever" place their mood in the context of adjustment to PI-ME/CFS. Others, such as "When I [crash … when I'm not] feeling good, it's hard to keep my emotional state stable because I [get really discouraged] or frustrated" place mood in the context of their function in the moment.

Use of Medications and Supplements was Common in PI-ME/CFS

PI-ME/CFS participants used many more medications than HV participants. PI-ME/CFS participants were taking on average 2.9 ± 2.5 medications and 4.6 ± 5.2 supplements daily. Ten PI-ME/CFS participants were taking drugs with central nervous system activity. Two PI-ME/CFS participants were taking antiviral medications (acyclovir and valacyclovir). One HV was taking pre-exposure HIV prophylaxis. Four PI-ME/CFS participants were on β-blockers (n=3) or hyperpolarization-activated cyclic nucleotide-gated channel blocker (n=1), all for symptomatic treatment of orthostatic complaints. PI-ME/CFS participants used a range of vitamins, nutraceuticals, and dietary supplements, including Vitamin D (n=7), Vitamin B12 (n=5), Magnesium (n=4), multivitamins (n=5), CoQ10 (n=5), and probiotics (n=2).

Body Mass Composition was Normal in PI-ME/CFS

There were no differences in body mass composition and Physiological Capacity Measures between PI-ME/CFS and HV participants. A subset of the cohort (HV 11, PI-ME/CFS 16) underwent whole-body composition and distribution measurements by dual-energy x-ray absorptiometry. No group differences in whole-body lean body mass (Figure S2A), fat mass (Figure S2B), bone mineral content (Figure S2C), % body fat (Figure S2D), or visceral fat mass (Figure S2E) were observed. The lean mass of the dominant arm (Figure S2F) and both legs

(Figure S2G) were also not different between the groups. There were also no differences in body composition between HV and PI-ME/CFS when sex-specific analyses were conducted.

Mitochondrial Genetics and Mitochondrial Function was Unrevealing in PI-ME/CFS

Mitochondrial genetics and a measure of mitochondrial function were normal in PI-ME/CFS. All PI-ME/CFS participants had variants in mitochondrial genes, however, most were of uncertain significance and of low (<15%) heteroplasmy. Some mutations in mitochondrial proteins were found in genes encoded by nuclear DNA. Of these, three individuals had variants in DNA polymerase gamma, two in methionyl-tRNA formyltransferase, two in lysosomal associated membrane protein-2, and two in arginyl-tRNA synthetase. The significance of these variants is uncertain.

Variants in the mitochondrial genome were annotated to the 162 mitochondrial genes in the PI-ME/CFS cohort (Data S10). Of these, 17 mitochondrial genes had missense variants in >90% of the patient cohort and eight mitochondrial genes had non-coding intronic variants in >90% of the patient cohort, with three genes, *EARS2*, *GARS*, and *OPA1,* having both missense and intronic variants in the patient cohort. All the variants identified were reported as non-deleterious.

Using a mitochondrial flux assay on peripheral blood mononuclear cells (PBMCs) in resting participants, no median group differences in basal (Figure S3A), maximal respiration (Figure S3B), or non-mitochondrial (Figure S3C) oxygen consumption were observed.

Muscle Histology was Unrevealing in PI-ME/CFS

A measure of muscular deconditioning was not different between the PI-ME/CFS and HV groups but some PI-ME/CFS participants had low values. Type2:Type1 muscle fiber median Feret diameter ratio (Type 2:1 mFd): Muscle biopsy samples from the vastus lateralis were stained with ATPase pH 9.4 stain and measured. No group differences were seen in Type 2:1 mFd (0.92 [0.88, 1.0] versus 0.83 [0.76, 1.03], p=0.1). The variance of Type 2:1 mFd was notably wider in PI-ME/CFS participants (Figure S3D).

No correlations were noted between median Type2:1 mFd and dominant lean arm mass (r(5)=0.14, p=0.8 versus r(7)=0.43, p=0.2) and lean leg mass (r(6)=0.1, p=0.8 versus r(7)=0.44, p=0.2).

Consumption of Dietary Fat was High and Fiber was Low in PI-ME/CFS

The Diet History Questionnaire II (DHQII) results indicated there were no group differences in total energy intake (1960.6 ± 790.9 versus 1640.2 ± 571.4 kcal, p=0.2), or energy intake derived from fat, carbohydrate, protein, or alcohol (Data S11). Dietary fiber intake was greater in HVs (14.5 ± 5.8 versus 11.1 ± 5.8 gm/1000 kcal; p=0.03). Seven-day food records results were similar to DHQII results except no differences were found between groups for dietary fiber intake and less energy from saturated fat was identified in HVs compared to PI-ME/CFS participants (9.6 ± 2.9 versus 13.5 ± 2.8 % kcals/day; p=0.01; (Data S11). Thus, PI-ME/CFS consume less dietary fiber and more saturated fat than HV participants.

No group differences in dietary intake of calcium, sodium, vitamin D, gluten or caffeine were noted. However, food records showed HVs had higher intake of dietary iron (8.3±2.6 versus 6.4 ± 0.9 mg/1000 kcal; p=0.05) and folate (279.5 ± 87.0 versus 207.6 ± 45.6 mcg/1000 kcal; p=0.03).

PI-ME/CFS individuals had Disruption of Sleep Patterns Unconfirmed by Polysomnography

PI-ME/CFS participants complained of significantly more disordered sleep symptoms than HVs, with increased scores on the Pittsburgh Sleep Quality Index (3.2 ± 2.5 versus 7.8 ± 3.4, p=0.0001), PROMIS Sleep Disturbance (40.4 ± 6.0 versus 55.3 ± 9.5, p=0.00002), and PROMIS Sleep Related Impairment (40.0 ± 7.1 versus 61.3 ± 6.9, p=0.000001). The qualitative experience of disordered sleep is evoked in this participant description: "There are times since I've been ill, I'm so sleepy I can't stay awake. I sleep a few hours and then I wake up [and feel like] I have a hangover for a couple of hours. But [the vast majority of the time] I don't sleep but I'm horizontal."

All PI-ME/CFS participants underwent polysomnography in a sleep laboratory. Two had mild periodic limb movements (PLM Index ≥ 5, < 25), two had mild sleep apnea (Apnea Hypopnea Index ≥ 5, < 15 per hour), and one had moderate sleep apnea (Apnea Hypopnea Index ≥ 15, < 30 per hour). None of these individuals noted substantial improvement after a six-week trial of CPAP. Sleep fragmentation was noted in 10 PI-ME/CFS participants (three mild, five moderate, two severe). Thus, PI-ME/CFS participants reported moderate sleep dysfunction not explained by polysomnographic evaluation.

Dysautonomia was Common in PI-ME-CFS

Twenty-four hour ambulatory ECG (8am to 8am) was used to assess heart rate variability (HRV) in the time and frequency domains as well as through non-linear methods. PI-ME/CFS participants showed diminished HRV by three time domain indices (SDNNi: 67.1 [IQR 58.9-77.1] versus 54.2 [46.1-64.5], p = 0.01; rMSSD: 38.7 [30.1-52.2] versus 25.1 [24.5-36.5], p = 0.02; pNN50: 12.5 [IQR 6.1-19.4] versus 3.7 [3.1-7.4], p=0.01; Figures 2B-2D).

PI-ME/CFS participants further showed altered frequency domain differences, including decreased high frequency (HF) power (Estimated mean 607 ± SE 7.1 versus 326 ± SE 7.1 ms2, p = 9.9E-85) and decreased low frequency (LF) power (1273 ± SE 13.8 versus 763 ± SE 13.8 ms2, p = 9.3E-78) when compared to HVs (Figures 2E-F, data shown in log scale).

Non-linear analyses further demonstrated group differences in SD1, SD2, and SD1/SD2. Group heart rates averaged over five-minute intervals (Estimated mean 71.7 ± 0.13 (SE) versus 74.5 ± 0.13 (SE) beats per minute, p = 03.2E-41) displayed two notable trends (Figure 2G). Increased heart rate in PI-ME/CFS participants throughout the twenty-four hour period suggests comparatively increased sympathetic activity. HVs had a comparatively significant decline in nighttime heart rate.

Head-up tilt table testing at 70 degrees from horizontal for up to 40 minutes was performed during which finger blood pressure (BP) was monitored continuously and upper arm blood pressure measured with a cuff every four minutes. Orthostatic BP decreases of ≥ 20 mmHg were similar for both groups (PI-ME/CFS=9/16, HV=7/17). The frequencies of excessive orthostatic tachycardia at 10 minutes also did not differ (PI-ME/CFS=6/16, HV=3/17)[2]. The occurrences of symptoms by 40 minutes did not differ between groups (PI-ME/CFS=7/16, HV=7/17).

Plasma levels of catecholamines were measured at several time points during the orthostatic challenge. The plasma epinephrine and plasma norepinephrine responses to being tilted are illustrated in Figures S4A and S4B. There was no difference in the epinephrine/norepinephrine ratio between the groups (Figure S4C).

Indices of baroreflex-cardiovagal and baroreflex-sympathoneural gain were obtained based on interbeat intervals and BP responses during Phase II of the Valsalva maneuver. The PI-ME/CFS group had a lower mean baroreflex-cardiovagal gain (baroslope mean ± SE: 5.8 ± 0.6 versus 4.6 ± 0.7 ms/mmHg, p=0.03; Figure 2H). There were several indices of baroreflex-sympathoneural function. The PI-ME/CFS participants had longer blood pressure recovery times (3.0 ± 0.2 versus 4.1 ± 0.4 sec, p=0.01), but the groups did not differ in Phase II (99 ± 19 versus 135 ± 23 mmHg-sec, p=0.2), Phase IV (60 ± 11 versus 87 ± 15 mmHg-sec, p=0.2), or the sum of Phase II+Phase IV baroreflex areas (159 ± 26 versus 222 ± 29 mmHg-sec, p=0.1).

Qualitative experience of dysautonomia was evoked in these participant descriptions. The description "If I try to do something upright even for a few minutes, I feel like I'm going to collapse. I get really hot, overheated, and my heart starts racing" relates to positional nature that these symptoms can have. Other descriptions such as "I have actually had four episodes of dizziness. [And] I just remembered [doctors are] always asking me if I'm dizzy. It's kind of a weird dizziness. If I were to describe it … it feels like my brain is floating lead when it occurs. And that does not make sense at all but it's the only thing I can think of to describe it, because there's this heaviness of a floating dizziness" indicate that not all descriptions of dizziness can be related to dysautonomia.

Effort Preference was Different between PI-ME/CFS and HV

Alterations in the "sense of effort" have been reported in the literature[3-5]. Motivation was assessed using the Effort-Expenditure for Rewards Task (EEfRT) which assesses effort, fatigue, and reward sensitivity (Figure S5A). Graphical examples of differential effects with the EEfRT task are depicted for effort, fatigue, and reward (Figures S5B-D). Replicating the original modeling strategy for the EEfRT[15], multiple models were evaluated to assess for group differences and assess for the presence of potential interaction effects. Model 1 tested the effects of reward value, reward probability, expected value, trial number, sex, and PI-ME/CFS diagnostic status on hard-task choice, without any interaction effects. Given equal levels and probabilities of reward, HVs chose more hard tasks than PI-ME/CFS participants (Odds Ratio (OR) = 1.65 [1.03, 2.65], p = 0.04; Figure 3A). For all of the other replicated models, which tested the significance of the effects of the interactions of diagnostic status and reward

probability (Model 2), diagnostic status and reward value (Model 3), diagnostic status and expected value (Model 4), diagnostic status, reward probability, and reward value (Model 5), and diagnostic status and prior reward feedback (Model 6), none of the interaction terms were significant, so these models were dropped from consideration in favor of Model 1 for the final analysis. A new model, which included the interaction of diagnostic status and trial number to assess whether HVs and PI-ME/CFS participants differed in their rate of fatigue, also produced a non-significant interaction term, and was therefore also dropped from further consideration. As Models 2 and 3 did not show differences in reward sensitivity and probability sensitivity by group, further analysis was not performed[6]. No difference in decision timeliness was observed as measured by task decision timeouts (0.3% versus 0.6%, p = 0.19).

A significant three-way interaction of diagnostic status, task difficulty, and trial number on task performance, measured by button press rate, was observed. Average button press rates declined significantly over time in the PI-ME/CFS participants, but only for easy tasks (Slope = -0.008, SEM = 0.002, p = 0.003; Figure 3B). This decline was not seen during the hard tasks, signifying that the declines were not due to fatigue (Figure 3B). A significant two-way interaction of diagnostic status and task difficulty on task completion, itself a function of button press rate, was also observed. HVs were more likely to complete hard tasks compared to PI-ME/CFS participants by an immense magnitude (OR = 27.23 [6.33, 117.14], p < 0.0001), but were no more or less likely to complete easy tasks compared to PI-ME/CFS participants (OR = 1.61 [0.31, 8.47], p > 0.05). Thus, the decline in button press rate on easy tasks observed over time in the PI-ME/CFS participants did not correspond to increased failure rate on the easy tasks. It appears that the PI-ME/CFS participants reduced their mechanical effort while maintaining performance on the easy tasks in a fashion not observed in HVs.

The Proportion of Hard-Task Choices was used as a correlate representing effort preference, the decision to avoid the harder task when decision-making is unsupervised and reward values and probabilities of receiving a reward are standardized.

The finding of a difference in effort preference is consistent with how participants describe pacing. One participant describes: “You have to make a conscious choice of how much energy [to use and] whether or not something is worth crashing for. It’s hard because no sane person would ever participant to suffer and … that’s what you’re doing [by choosing] an activity that … will make you crash. You are going to suffer… You have to decide what gives you meaning and what is worth it to you.”

Physical Performance Measures showed Differences between PI-ME/CFS and HV groups
A subset of participants (11 HV, 11 PI-ME/CFS) wore activity monitors on their hip and wrist for 12.6 ± 5.2 days at home. HV had greater hip activity as measured in 3-D vector magnitude counts/day (60,4988 ± 29,0887 versus 36,7415 ± 15,1080; p=0.03), steps/day (7,111 ± 2,432 versus 3,618 ± 1,682; p<0.001), and minutes of moderate physical activity (i.e. 3-6 x basal energy expenditure; 40.64 ± 37.4 versus 6.4 ± 7.0; p=0.007). No differences in sedentary (1021 ± 146 versus 988 ± 177 min/day), light movement (283 ± 71 versus 230 ± 93 min/day) vigorous activity (1.6 ± 3.7 versus 0.2 ± 0.6 min/day), or daily wrist activity (1,795,199 ± 582,291 versus

1,901,063 ± 654,127; p=0.7) were noted between the groups. This measured difference is evoked in this participant's description of their physical abilities: "I can't really walk more than half a block … that would be pushing it [and] I probably wouldn't be doing two loads of laundry that same day. I could, on my very best day, [go up and down the stairs a few times to] do two loads of laundry, driving, walking a short distance, and cooking dinner. I do have a stool that I sit on [when cooking]."

Using a dynamometer, maximal grip strength and time to failure to maintain 50% maximal strength was measured. No group difference in maximal grip strength was noted in the dominant hand (37.8 ±11.5 versus 34.5 ±12.0 kg, p=0.35; Figure 3C). Time to failure was shorter in the PI-ME/CFS participants (30.5 ±10.3 versus 15.7 ±10.0 seconds, p=0.0002; Figure 3D). These findings are evoked in this participant's description of their strength: "Interestingly, I can still [grip strong]… Everyone says I'm strong but when it comes to [holding it for a while, I] don't do well at all… Even though I have this incredible fatigue, I can still get up and do something and look normal."

For the single grip test, maximum grip force correlated with lean arm mass (Figure S6A) and did not correlate with effort preference (Figure S6B) or Type2:1 mFd (Figure S6C) for both groups. Time to failure correlated with effort preference in PI-ME/CFS but not in HVs (Figure S6E). Time to failure did not correlate with lean arm mass (Figure S6D) or Type2:1 mFd (Figure S6F) in either group.

To assess physical fatigue, participants maintained their grip at 50% of maximum voluntary contraction (MVC) in successive blocks of 30 seconds interspaced with 30 second rest blocks (Figure S7A). Electromyography (EMG) of the flexor and extensor carpi radialis (FCR, ECR) and abductor pollicis brevis (APB) were recorded to quantify the grip.

Single pulse transcranial magnetic stimulation (TMS) was done to probe excitability of the primary motor cortex (M1) via the motor evoked potential (MEP) of the APB during the rest blocks.

The development of fatigue during the grip task was analyzed by comparing groups and time. For muscle fatigue, we used the Dimitrov index (DI) [7,8] to evaluate the shift in EMG frequency power within blocks.

MVC did not significantly differ between groups (t(12) = 1.4, p = 0.18). Repetitive grip testing showed a significantly different rapid decline in force (-1.2 ±4 versus -6.4 ±4 kilogram-force, t(12) = 2.46, p = 0.03), a significantly lower number of non-fatigued blocks (Figure 4A), and a relative decrease in slope of the DI (0.2 ±0.5 versus -0.43 ±0.3, t(12) = 3.2, p=0.008; Figure 4B) in PI-ME/CFS participants but remained constant in HVs. This indicates that HVs performed consistent muscular work to generate lactate throughout the entire task while PI-ME/CFS participants only performed enough muscular work to generate lactate early in the task but then subsequently stopped performing enough muscular work. The amplitude of the MEPs of HVs significantly decreased over the course of the task, consistent with post-exercise

depression [9], while the amplitudes of the MEPs of PI-ME/CFS participants significantly increased (-0.13 ±0.2 versus 0.13 ±0.2 MEP units; $t(12) = 2.4$, $p = 0.03$; Figure 4C). The M-max remained constant in both groups. This indicates that M1 remained more excitable for PI-ME/CFS, suggesting reduced motor engagement from this group [10].

Total motor output, the sum of all the forces generated during the entire task, did not correlate with effort preference (Figure S7B) or lean arm mass (Figure S7C). No significant correlation was noted between total motor output and Type2:1 mFd (Figure S7D), suggesting that muscular deconditioning was not responsible for group differences noted in muscle strength measures.

The repetitive grip force paradigm was repeated in the fMRI environment and blood oxygen level dependent (BOLD) signal of the brain was measured. Grip force (decline rate: -6.3 ±5 versus -11.9 ±3, $t(16) = 2.83$, $p = 0.01$; Figure S7E) and fatigue VAS score ($t(11) = 4.6$, $p = 0.0007$) were consistent with the results above.

BOLD signal on fMRI of PI-ME/CFS participants decreased across blocks in the bilateral temporo-parietal junction (TPJ), bilateral superior parietal lobule, and right temporal gyrus in contradistinction to the increase observed in HVs, resulting in significant group differences ($F(3,45) = 5.4$, voxel threshold $p \leq 0.01$, corrected for multiple comparisons $p \leq 0.05$, $k > 65$; Figures 4D-E).

Cardiopulmonary Exercise Test showed Differences between PI-ME/CFS and HV groups

A subset of the cohort underwent a cardiopulmonary exercise test (CPET) (HV=nine; PI-ME/CFS =nine) by upright cycle ergometry (Figure S8A). To achieve the peak work rate during an optimum testing time of eight to twelve minutes for both groups, PI-ME/CFS participants were tested at a lower ramp protocol of 15 Watts/min compared to 15-25 Watts/min for HVs. All but one PI-ME/CFS participant reached the peak respiratory exchange ratio (RER) of 1.1 or higher and RER was similar between the groups (1.30 ± 0.08 versus 1.24 ± 0.12, p=0.2), which demonstrates that all participants performed at a level sufficient to require anaerobic respiration. Effort preference did not correlate with peak power in PI-ME/CFS participants ($r(6)=0.13$, $p=0.8$) which suggests that effort preference did not impact CPET performance, perhaps related to experimental incentives to push their limits. This is opposite to the behavior of the HVs, where effort preference trends towards a moderately strong correlation with peak power on CPET despite not correlating with grip strength tasks (Figure S8B). This seems congruent with participant descriptions of performing CPET such as: “I was biking and I wanted to push myself as hard as I could for the research ... and there was this point where I almost stopped [and] thought I was going to stop… My revolutions per minute dipped [and they were] ready to catch me. I [then asked] if I could lean forward on my elbows. They let me, so I did, and then I kept going… Then there was this point where [I could not go] any longer and [I] made a hand motion and slumped off the bike with their help… I don't know if I can remember a time where I felt [that fatigued] immediately in that moment.”

During CPET, the ratio of Ventilation/VCO_2, oxygenation of the quadricep muscle as measured by Near Infrared Spectroscopy, or in gross mechanical efficiency were not different between groups (Figures S8C-E).

Peak power (222.1 ± 46.5 versus 158.3 ± 91.1 Watts, p=0.08), peak respiratory rate (38.9 ± 6.8 versus 28.7 ± 6.2 breaths/minute, p=0.005), and peak heart rate (absolute: 172.6 ± 12.3 versus 149.7 ± 29.7, p=0.07) were all lower in the PI-ME/CFS participants, reflecting a differential of cardiorespiratory capacity between the groups (Figure 5A-C). Peak power highly correlated to lean leg mass in both groups (r(9)= 0.76, p=0.02 versus r(8)=0.91, p=0.001; Figure S8G). Peak power did not correlate with Type2:1 mFd in either group (r(7)= 0.05, p=0.9 versus r(5)=0.82, p=0.09; Figures S8H).

Peak VO2 was lower in PI-ME/CFS participants (32.8 ± 6.6 versus 20.1 ± 7.9 mL/kg/min, p=0.004; Figure 5D), a difference of approximately 3.3 metabolic equivalent of task units (METs). Peak VO_2 strongly correlated with Type2:1 mFd (r(7)=-0.08, p=0.9 versus r(5)=0.94, p=0.02; Figure S8I) in PI-ME/CFS but not HVs, and did not correlate with lean leg mass (r(7)= -0.08, p=0.8 versus r(6)=0.66, p=0.08) in either group.

PI-ME/CFS participants achieved a lower percent of their predicted VO_{2peak} (96.2 ± 33.7% versus 71.4 ± 15.9%, p=0.004) as determined by the Wasserman-Hansen cycling equation[11,12], based on each participant's age, sex, height, and weight (Figure 5E). Similarly, PI-ME/CFS participants reached a lower percent of their age predicted maximal heart rate (220 beats·min^{-1} - age in yrs; 95.9 ± 5.3% versus 83.7 ± 13.6%, p=0.02; Figure S8F).

PI-ME/CFS participants had a higher resting heart rate (absolute: 64.6±7.8 versus 72.9+9.8, p=0.07) and a lower peak heart rate (absolute: 172.6+12.3 versus 149.7+29.7, p=0.07), leading to a lower heart rate reserve (107.9 ± 15.4 versus 76.8 ± 28.1 bpm, p = 0.02; Figure 5F). Chronotropic incompetence, as measured by age-predicted HRR (%pHRR), was noted in five of eight PI-ME/CFS and one of nine HVs (X^2 (1, n=17) = 4.9, p = 0.03). The slope of the heart rate response during the CPET was also lower in PI-ME/CFS participants compared to HVs (1.03 ± 0.16 versus 0.70 ± 0.27, $p < 0.01$; Figure 5G). PI-ME/CFS participants have less cardiovascular capacity to respond to an exercise challenge.

The anaerobic threshold (AT), the point where the body switches from mostly aerobic to anaerobic metabolism, was achieved within a similar time period in both groups (298.3 ± 99.6 versus 279.3 ± 195.7 seconds, p=0.8). The switch to anaerobic metabolism occurred at lower peak VO2 levels in the PI-ME/CFS than in HVs (16.0 ± 4.3 versus 11.0 ± 3.9 mL/kg/min, p=0.02; Figure 5H), a difference of approximately 1.4 METs. This data demonstrates less efficient oxidative respiration in PI-ME/CFS participants. Type2:1 mFd strongly correlated with VO2 at AT (r(5)= -0.33, p=0.5 versus r(3)=0.92, p=0.03; Figure S8J) and trended to correlate with power at AT (r(5)= 0.026, p=0.6 versus r(3)= 0.75, p=0.1; Figure S8K) in PI-ME/CFS but not HVs.

Salivary Cortisol Levels were Lower in PI-ME/CFS in Response to Exercise

No group differences in morning (0.49 ± 0.3 versus 0.37 ± 0.15 µg/dL, p = 0.18), noon (0.16 ± 0.11 versus 0.18 ± 0.05 µg/dL, p = 0.4), or nighttime (0.07 ± 0.02 versus 0.08 ± 0.04 µg/dL, p = 0.7) measurements of salivary cortisol were noted. After CPET, PI-ME/CFS participants had a lower cortisol response than HVs (0.43±0.28 versus 0.22±1.2 µg/dL, p=0.03; Figure S8L).

Measures of Energy Expenditure and Respiratory Exchange were Similar in PI-ME/CFS and HV
The subset of the cohort that underwent a cardiopulmonary exercise test (CPET) (HV=nine; PI-ME/CFS =nine) underwent whole-room indirect *calorimetry* that started the day prior to CPET and was completed 72 hours after CPET.

All participants spent this time at rest and there were no differences in activity while in the chamber, as measured by microwave activity (8.9 ± 3.6 vs 7.6 ± 3.5 % of time active; p = 0.4), hip actigraphy (86.4 ± 45.9 versus 95.2 ± 71.9 3-D vector magnitude counts/min; p=0.7) and wrist actigraphy (630.3 ± 335.3 versus. 775.2 ± 362.6 3-D vector magnitude counts/min; p=0.3).

No differences in total (1947 ± 249 versus 1976 ± 437 kcal/day; p=0.4), sleeping energy expenditure (1497 ± 210 versus 1554 ± 335 kcal/day; p=0.2), or respiratory quotient (VCO2/VO2) (Daily: 0.86 ± 0.03 versus 0.88 ± 0.04, p=0.2; Sleep: 0.84 ± 0.03 versus 0.86 ± 0.03, p=0.1) were observed between the groups after a baseline day of rest, even after adjustment for known covariates, i.e. age, sex, fat mass, and fat-free mass [13] (Figures S9A-D, Data S12).

In the CPET cohort, no group differences were noted in energy intake or in total body energy use, sleeping energy use from baseline in the 72 hours following the exercise challenge after controlling for age, sex, fat mass, and fat-free mass. Similarly, no group differences in the respiratory quotient or activity level in the calorimeter were observed 48 hours after exercise (Data S12).

No group differences in basal, maximal respiration, or non-mitochondrial oxygen consumption were observed at baseline, 24, 48, or 72 hours after CPET (Figures S9E-G).

Thus, there were no differences in energy expenditure and respiratory exchange between the PI-ME/CFS and HV groups.

PI-ME/CFS Individuals had Substantial Cognitive Symptoms but Normal Neurocognitive Testing
Perception of cognitive function was measured with the Multiple Ability Self-Report Questionnaire (MASQ). Total cognitive complaints were greater in PI-ME/CFS (66.9 ± 14.7 versus 92.1 ± 17.6, p=0.00006). PI-ME/CFS participants had more self-reported deficits in all domains measured, including attention (14.3 ± 4.7 versus 20.6 ± 4.1, p=0.0005), verbal memory (15.0 ± 3.4 versus 20.9 ± 4.9 versus, p=0.0002), visuoperceptual (9.9 ± 3.2 versus 13.0 ± 4.1, p=0.02), language (12.6 ± 2.7 versus 18.9 ± 4.6 versus 12.6 ± 2.7, p=0.00004), and visual memory (13.3 ± 3.1 versus 16.1 ± 4.3, p=0.09; Figure 1C). The qualitative experience of cognitive symptoms is evoked in this participant description: “It feels like, thinking is really slowed, like my head is really fuzzy, full of cotton. It feels like my brain’s shutting down. Not

sure how to describe it, just kind of like a low power state, low energy state. And also I guess there's a heaviness. There's kind of a sensation of my head feels kind of heavy."

Participants performed a neuropsychological testing battery which took on average three hours to complete. There were no group differences on performance of any of the 15 neuropsychological tests administered (Data S13). Over the course of the testing battery, there was no evidence of a differential degradation of performance between the groups. Performance of the Test of Variables of Attention (TOVA), a sensitive measure of sustained attention which was completed after two hours of testing, was no different between the groups (1.1±3.6 versus 0.9±4.7, p=0.9). No correlations were noted between any of the 15 neuropsychological tests administered and effort preference.

A 0-100 points visual analog scale (VAS) was used to assess fatigue, effort, and performance over the testing period in a subgroup of 8 HV and 10 PI-ME/CFS participants (Figures S10A-D). PI-ME/CFS participants noted substantially more mental (15.6 ± 22.0 versus 34.9 ± 23.6, p=0.04) and physical (19.2 ± 23.7 versus 41.8 ± 25.1, p=0.02) fatigue immediately prior to starting the testing battery. Both groups reported incremental increases in mental and physical fatigue over the testing period. The two-way interaction of diagnostic status and trial number was not significant, signifying that both groups endorsed self-reported fatigue at an equal rate. Both groups reported giving a high level of effort (87.6 ± 16.8 versus 97.4 ± 4.7, p=0.05) and a similar level of perceived performance (76.8 ± 25.0 versus 74.2 ± 18.9, p=0.9) over the entire testing period.

More limited testing was also performed before and 48 hours after exercise stress in the CPET cohort. Performance on the BVMT and TOVA did not change for either group 48 hours after CPET.

Cerebrospinal Fluid Catechol levels Corelated with Perception and Behavior, but not Cognitive Performance

A schematic of the catechol pathway can be viewed in Figure S11A. The PI-ME/CFS group had mean decreases in cerebrospinal fluid levels of DOPA (746 ± 200 versus 605 ± 138 pg/ml; p=0.02; Figure 6A), DOPAC (350 ± 130 versus 263 ± 111 pg/mL, p=0.04; Figure 6B), and DHPG (1922 ± 547 versus 1466 ± 334 pg/ml; p=0.006; Figure 6C). Levels of norepinephrine (NE) (145 ± 71 versus 116 ± 43 pg/mL, p=0.16), cys-DOPA (278 ± 61 versus 249 ± 107 pg/mL, p=0.3), and dopamine (8.5 ± 4.6 versus 9.7 ± 6.2pg/mL, p=0.5) did not differ between the groups.

Catechol data was reanalyzed to determine the impact of central acting medications on the results. Excluding participants using amphetamines did not alter the above results. Excluding participants using benzodiazepines or tricyclic compounds, the significant differences in DHPG and DOPA remained. Excluding participants using serotonin-norepinephrine uptake inhibitors, DHPG, DOPA, DOPAC, and Cys-DOPA were significantly lower in the PI-ME/CFS group.

In the grip strength task, NE correlated with both Time to Failure (r(13)= 0.61, p=0.02; Figure 6D) and effort preference (r(12)= 0.65, p=0.01; Figure 6E) for PI-ME/CFS participants. Dopamine

correlated with Time to Failure on the grip strength task (r(12) = 0.65, p=0.01; Figure 6F) but not effort preference (Figure S11B). For HVs, DHPG correlated with effort preference (r(12)= -0.60, p=0.02; Figure 6G) but not Time to Failure (r(16)=0.02, p=0.9; Figure S11C).

Several cognitive symptom measures correlated with catechols in PI-ME/CFS participants. Specifically, NE correlated with MMPI total cognitive complaints (Figure S12A). Dopamine correlated with visual-spatial memory (Figure S12B). cys-DOPA correlated with MMPI total cognitive complaints (Figure S12C), language (Figure S12D) and visuoperception (Figure S12E). DOPAC correlated with MMPI total cognitive complaints (Figure S12F) and attention/concentration (Figure S12G). DHPG correlated with attention/concentration complaints (Figure S12H) and visuoperception (Figure S12I). No significant correlations with cognitive symptom measures were seen in HVs. In contradistinction, few catechols correlated with objective neurocognitive performance in PI-ME/CFS. Only the Wisconsin Card Sort Test correlated with DOPA in PI-ME/CFS participants (Figure S12J). For HVs, several correlations between catechols and neuropsychological tests were observed. Norepinephrine correlated with performance on the WAIS Symbol Search Scale (Figure S12K), DOPA with both the Brief Visual Memory Test-Revised Recall (Figure S12L) and Hopkins Verbal Learning Test-Revised Total score (Figure S12M), and cys-dopamine (Figure S12N) and DOPAC (Figure S12O) with the WAIS Coding Scale. These correlations suggest that perception and behavior, but not cognitive performance, are related to catechols in PI-ME/CFS participants.

Cerebrospinal Fluid Metabolites were Dysregulated in PI-ME/CFS

Catechols metabolites were different in PI-ME/CFS compared to the HV group, with a downregulation of tryptophan metabolites.

Metabolites, 388 named and 57 unnamed, in cerebrospinal fluid were analyzed. A group difference in these central nervous system compounds was observed (Figure 6H). Univariate analysis identified several differentially expressed metabolites that were statistically significant after multiple comparison correction (Figure 6I). Of the top 15 differentially expressed metabolites, several metabolites in the tryptophan pathway were downregulated in PI-ME/CFS participants. Other metabolites that were decreased included glutamate and dopamine 3-O-sulfate. Analysis of branch chain amino acids showed a decrease in butyrate, as well as decreased metabolites related to the tricarboxylic acid pathway in PI-ME/CFS participants (Data S14A). There was also an overall decrease in polyamine metabolites. Partial least square discriminant analysis (PLSDA) of metabolites in case-control analysis identified eight metabolites with variable importance in prediction (VIP) score >1. No pathway level inference could be made, and a subset of VIP metabolites were also differentially expressed metabolites as noted above (Figures S13A-C, Data S14B).

PLSDA of metabolites in the sex-separated cohorts were also performed. In the male cohort, the VIP metabolites included threonine and glutamine whose average expression was lower in PI-ME/CFS patients compared to HVs. Since the tryptophan pathway metabolites were not identified in this analysis, all samples were included irrespective of the males' selective serotonin reuptake inhibitor (SSRI) drug intake history (Figures 6J, Figure S13D, Data S14C). In

the female cohort, VIP metabolites functioning in the tryptophan pathway and butyrate metabolism were differentially expressed (Figures 6K, Figure S13E, Data S14D). Since several participants had a history of using selective serotonin reuptake inhibitor drugs, the PLSDA analysis of metabolite profile in the female cohort was performed after excluding the participants using SSRIs. The metabolites in tryptophan metabolism were identified as important predictors in this smaller cohort, suggesting that the changes in the tryptophan metabolism is not a result of the drug uptake.

PI-ME/CFS Individuals had Immune Activation and Immune Exhaustion

Alterations in B-cells and PD-1 positive CD8 T-cells were observed with flow cytometry of blood and cerebrospinal fluid.

Immunophenotyping by flow cytometry was performed on blood and cerebrospinal fluid (Figures 7A-F, Data S15A-D). No group differences in CD4/CD8 ratio, activated CD4 T cells (CD25), memory follicular helper T cells ($CD45RA^-$, $CXCR5^+$), CD4 and CD8 effector memory T cells, CD4 and CD8 effector T cells, B cells, or B cells/monocyte ratio were observed. An increase in percentage of naïve and decrease in switched memory B-cells in blood were observed in PI-ME/CFS participants (Figures 7A-B). Two PI-ME/CFS participants had detectable antibody secreting B cells in the cerebrospinal fluid; these participants did not have measured oligoclonal bands or autoantibodies. There was no difference in the frequency of natural killer (NK) cells between the groups, but a subset of PI-ME/CFS participants had elevated NK CD56 bright/dim ratio in the cerebrospinal fluid.

Markers of T-cell activation, $PD\text{-}1^+$ CD8 T-cells, were elevated in the cerebrospinal fluid of PI-ME/CFS participants (Figure 7C) but not in blood. $PD\text{-}1^+$ CD4 T-cells did not change in blood or cerebrospinal fluid. To expand on these observations, other T-cell markers (TIGIT, CD244, and CD226) were added to the flow cytometry panel and performed on a subset of participants. $CD226^+$ CD8 T-cells were decreased in blood (Figure 7D) of PI-ME/CFS participants, with no change in expression of CD244 or TIGIT (Data 15B and 15D).

When the group differences in CD8+ T-cells were assessed after stratification by sex, male PI-ME/CFS participants had increased CXCR5 expression on CD8+ T-cells in cerebrospinal fluid (Figure 7E). Female PI-ME/CFS participants were noted to have increased CD8+ naïve T-cells in blood (Figure 7F).

Sex-based Differences in Gene Profiles of Peripheral Blood Mononuclear Cells

Peripheral blood mononuclear cell gene expression profiles are different in PI-ME/CFS compared to HV participants, but only when stratified by sex.

Gene expression of peripheral blood mononuclear cells (PBMC) was profiled in HV and PI-ME/CFS. Analysis of PBMC gene expression for all participants by principal component analysis (PCA) revealed no outliers (Figure 8A). 614 differentially expressed (DE) genes clustered the samples based on the disease status. However, exploration of PCA plot showed that samples clustered based on their sex (Figure 8B). Assessment of interaction between sex and disease

status were not significant. This suggested sex as a potential confounder impacting gene expression. Hence DE analysis in male and female cohorts were performed which identified distinct subset of DE genes, with only 34 (<5%) genes overlapping between the cohorts (Figure 8C, Data S16A-B). Further, PCA of DE genes in each sex cohort clustered the samples into distinct HV and PI-ME/CFS groups (Figures 8D, 8F).

887 DE genes in the male HV and PI-ME/CFS cohorts enriched ($p<0.05$) in ubiquitin, IL-10, T-cell, and NF-kB pathways (Figures 8H-I). A subset of DE genes was part of the STAT4-TLR9 protein-protein interactome and were upregulated in male PI-ME/CFS participants (Figure S14A). 849 DE genes identified in the female HV and PI-ME/CFS cohorts, primarily enriched in B-cell proliferation processes (Figures 8J-K), and a subset of DE genes identified in the B-cell interactome were upregulated in female PI-ME/CFS participants (Figure S14B). Additionally, DE genes were also enriched in cytokine and lymphocyte proliferation processes. These data are consistent with the expansion of naïve B-cells observed in PI-ME/CFS participants by flow cytometry.

Sex-based Differences in Proteomics of Serum and Cerebrospinal Fluid

Proteomics did not distinguish PI-ME/CFS from HV participants but did suggest differences exist after stratification by sex. 1317 aptamers were used for detection of various proteins in serum and cerebrospinal fluid. PCA analysis of aptamers measured in serum and cerebrospinal fluid did not identify outliers. Univariate analysis of the aptamer datasets did not identify any FDR-corrected statistically significant differential features between PI-ME/CFS and HV participants (Data S17A-B). Within each sex separately, exploratory multivariate analyses suggested a subset of aptamers were predictive of PI-ME/CFS status which are reported for potential validation (Figures S15A-D; Figures S16A-D; Data S17C-F).

Sex-based differences in Gene Expression were Validated in other Data Sets

Sex stratification was observed to impact the ability to distinguish ME/CFS from HV participants in two independent publicly deposited datasets:

To understand if this observation of sex confounding was generalizable to other ME/CFS populations, publicly deposited ME/CFS datasets with RNASeq data were identified and analyzed. One study, NCBI GEO GSE130353, contained RNA sequencing generated from monocytes on HV and ME/CFS male and female participants. Sex was inferred using XIST gene expression[14] . This data set had nine males and 11 females (HV 10 and ME/CFS 10) of which three were excluded from analysis due to low gene assignment. Groupwise, 315 DE genes were observed in ME/CFS and HV groups (PCA shown in Figure S17A). Concordant with the PBMC gene expression profile, PC3 and PC4 clustered samples into groups that corresponded to the sex of the participants (Figure S17B). 638 DE genes were identified from the ME/CFS and HV comparison in the male cohort (PCA shown in Figure S17C) and 420 DE genes in the female cohort (PCA shown in Figure S17D). Of these, only 23 genes were differentially expressed in both male and female ME/CFS participants when compared to HVs (Figure S17E).

To further investigate whether the perturbations in the IL10, T cell and lymphocyte processes identified in the PBMC gene expression profiling could be observed in male ME/CFS participants in external studies, the methylation of CD3+ T cells from the dataset GSE156792 was assayed in sex-separated cohorts. First, the sex of the samples in this study were inferred based on their Y chromosome coverage. Second, differentially methylated positions (DMPs) were identified in each of the cohort, which were annotated to the genomic loci. The PCA computed using the methylation fractions of the DMPs in the male cohort distinctly separated the HV from ME/CFS participants. 138 genes with DMPs overlapped with the DE genes from the PBMC HV versus PI-ME/CFS comparison. Notably, pathway enrichment analysis of genes with DMPs nominally enriched in IL10 cytokine and NF-kB related processes, which is concordant with the pathway enrichment results from the male group PBMC analysis (Figures S17F-G). The DMPs identified in the female cohort, on the other hand, moderately clustered the samples in HV and PI-ME/CFS groups. However, pathway enrichment analysis of genes with DMPs in the female cohort enriched in T cell proliferation and differentiation processes (Figures S17H-I). Consistent with the DMP signature in the external dataset, the flow cytometric analysis of immune cells population in this study's female cohort identified increased naïve CD8+ T cells, and decreased CD225+ T cells in PI-ME/CFS participants. Since the methylation signature was assayed in the CD3+ T cells, further analysis of the B cell subpopulation will be required to test their role in PI-ME/CFS pathology in female patients.

Natural Killer (NK) Cell function was Normal

No difference in Natural Killer cell function was observed between PI-ME/CFS and HV groups. No group differences were noted in NK lytic units (5.0 ± 2.8 versus 4.4 ± 3.3, p=0.58) or CD16/CD56 ratio (10.1 ± 3.0 versus 11.0 ± 5.0, p=0.53).

Cellular Senescence was not seen in PI-ME/CFS

No difference in cellular senescence was observed between PI-ME/CFS and HV groups. Growth differentiation factor-15 (GDF-15) level was measured as a marker of senescence in plasma and no difference was found between groups (101.8 ± 36.4 versus 97.6 ± 34.6 pg/mL, p = 0.84).

Detailed Autoantibody Testing was Unrevealing in PI-ME/CFS

No consistent pattern of autoimmunity was observed in PI-ME/CFS. Autoantibodies against several other known or potential autoantigen targets were measured by LIPS as coded samples from the participants. From testing, occasional rare low-level seropositive autoantibodies against the target proteins were detected in both groups. For example, one HV and one PI-ME/CFS participant had antibodies to tyrosine hydroxylase and one HV had antibodies to Gad65. However, statistical analysis of the seropositivity by Fisher's exact testing with contingency tables revealed no significant differences in seroprevalence between the two groups.

Transposable Elements and Endogenous Retroviruses were Not Activated in PI-ME/CFS

The PBMC RNAseq data was used to assess the expression levels of transposable elements (TE), which included the human endogenous retroviruses (HERVs) derived from exogenous retrovirus infection in HV and PI-ME/CFS samples. Differential expression analysis did not identify HERVs

or other autonomous (Long INterspersed Elements (LINEs)) or non-autonomous (Short INterspersed Elements (SINEs)) TEs to be differentially expressed between HV and PI-ME/CFS (Data S18).

Sex-based Differences in Gene Expression Patterns in Muscle

Muscle cell gene expression profiles are different in PI-ME/CFS compared to HV participants, but only when stratified by sex. RNA Sequencing was performed on muscle samples collected from the vastus lateralis muscle. PCA analysis of muscle gene expression did not identify any outliers (Figure 9A, Data S19A). The PCA identified a PC that clustered the samples based on sex (Figure 9B, Data S19B-C). Only 15 DE genes were common in male and female cohorts (Figure 9C), consistent with prior observations. In the male cohorts, PCA of the 593 DE genes robustly clustered samples based on the disease status (Figures 9D-E). Genes upregulated in the PI-ME/CFS males were enriched in gene expression and mRNA processes (Figure 9H) and the downregulated genes were enriched in hexose metabolism and mitochondrial processes (Figure 9I). Several upregulated genes involved in fatty acid beta-oxidation were noted to be part of a protein-protein interactome (Figure S18A). In the female cohorts, PCA of the 328 DE genes showed distinct clusters of HV and PI-ME/CFS samples (Figures 9F-G). DE Genes upregulated in the PI-ME/CFS females enriched in growth hormone receptor signaling and ubiquitin transferase function (Figure 9J). Downregulated genes were involved in fatty acid oxidation and mitochondrial processes (Figure 9K). A subset of these downregulated fatty acid oxidation genes was highly inter-connected (Figure S18B).

Sex-based Differences in Lipid Profiles

Lipidomic profiles are different in PI-ME/CFS compared to HV participants, but only when stratified by sex. Univariate analysis of the lipidomic data did not identify statistically significant differentially expressed lipids between HV and PI-ME/CFS groups (Data S20). Multivariate analysis in all participants as well as in male and female cohorts separately identified several lipid molecules as important variables in prediction (Figures S19A-C). Consistent with the gene expression analysis, sex differences were observed here too. In the male cohorts, greater than 50 variables were identified as important in predicting the cases and controls. Triacyl-glycerol (TAG) species with different carbon lengths were increased in PI-ME/CFS participants (Figure S19B). In the female cohort, a smaller subset of lipid species (<20) classified the group status. TAG species were again identified among the top variables but were decreased in PI-ME/CFS females (Figure S19C). The sex specific lipid signatures found in this study are consistent with a prior lipidomic analysis[15].

Gut Microbiome was Different in PI-ME/CFS

The constitution of the gut microbiome in PI-ME/CFS participants is less diverse and dissimilar from that of HV participants without any discernible difference in metabolites.
Stool samples were collected from PI-ME/CFS and HV participants for microbial metagenomic analysis. For the HVs that were sampled, the primary residences were mostly in Maryland and Virginia (76% of HV group) with two individuals from South Carolina and two from Utah. For the PI-ME/CFS participants sampled, the primary residences were diverse, representing twelve different US states and Canada.

Alpha diversity differences (measured by the number of present taxa) were observed between groups, with HV having a greater number of taxa than the PI-ME/CFS participants (p=0.002; Figure S20A). There was no significant difference observed by the Inverse Simpson Index (p= 0.8; Figure S20B) between the two groups. When alpha diversity was assessed within sex, the HV males had a higher number of taxa than the PI-ME/CFS males (p=.008). This difference was not observed within the females (p=0.7). Beta diversity, as measured by Bray-Curtis dissimilarity, demonstrated significant differences in microbial community composition between the groups (p<0.008; Figure S20C). This finding remained when beta diversity was assessed within sex for the males (p= 0.04), but not within the females (p= 0.3). When analyzed to determine which taxa were responsible for differences in diversity, 22 species were significantly higher and 26 species were significantly lower in the PI-ME/CFS participants (Figure S20D).

Stool Metabolites were Normal in PI-ME/CFS

A total of 34 metabolites were identified within the stool samples for the HV and PI-ME/CFS participants using nuclear magnetic resonance spectroscopy (Data S21). Untargeted stool metabolomics did not show any significant differences between the HV and PI-ME/CFS participants.

Clinical Course of PI-ME/CFS revealed Spontaneous Improvement in Some

Some PI-ME/CFS participants had clinically notable improvements over time. All participants were contacted between 11/2021 and 7/2022 to inquire about changes in their clinical condition. One PI-ME/CFS and one healthy participant were unable to be contacted. Three PI-ME/CFS participants reported full recovery without being able to attribute it to a specific intervention and one attributed full recovery to being infected with SARS-CoV-2. All other PI-ME/CFS participants reported being better at accommodating their illness but remained unchanged in their level of disability. One PI-ME/CFS participant reported having an in-situ breast cancer that was treated with excision and radiation therapy. No other new medical diagnoses were reported by the PI-ME/CFS participants. One healthy volunteer reported developing post-Lyme disease and chronic fatigue symptoms, one reported having low grade breast cancer treated with excision and radiation therapy and a single episode of nephrolithiasis, and one reported developing finger psoriasis that resolved with topical therapy. No other new medical diagnoses were reported by the HVs.

REFERENCES:

1 Bakkers, M. *et al.* Intraepidermal nerve fiber density and its application in sarcoidosis. *Neurology* **73**, 1142-1148 (2009). https://doi.org:10.1212/WNL.0b013e3181bacf05

2 Freeman, R. *et al.* Consensus statement on the definition of orthostatic hypotension, neurally mediated syncope and the postural tachycardia syndrome. *Clin Auton Res* **21**, 69-72 (2011). https://doi.org:10.1007/s10286-011-0119-5

3 Lawrie, S. M., MacHale, S. M., Power, M. J. & Goodwin, G. M. Is the chronic fatigue syndrome best understood as a primary disturbance of the sense of effort? *Psychol Med* **27**, 995-999 (1997). https://doi.org:10.1017/s0033291797005370

4 Metzger, F. A. & Denney, D. R. Perception of cognitive performance in patients with chronic fatigue syndrome. *Ann Behav Med* **24**, 106-112 (2002). https://doi.org:10.1207/S15324796ABM2402_07

5 Barhorst, E. E. *et al.* Elevated Perceived Exertion in People with Myalgic Encephalomyelitis/Chronic Fatigue Syndrome and Fibromyalgia: A Meta-analysis. *Med Sci Sports Exerc* **52**, 2615-2627 (2020). https://doi.org:10.1249/MSS.0000000000002421

6 Cooper, J. A. *et al.* Effortful goal-directed behavior in schizophrenia: Computational subtypes and associations with cognition. *J Abnorm Psychol* **128**, 710-722 (2019). https://doi.org:10.1037/abn0000443

7 Dimitrova, N. A., Hogrel, J. Y., Arabadzhiev, T. I. & Dimitrov, G. V. Estimate of M-wave changes in human biceps brachii during continuous stimulation. *J Electromyogr Kinesiol* **15**, 341-348 (2005). https://doi.org:10.1016/j.jelekin.2005.01.005

8 Arabadzhiev, T. I., Dimitrov, G. V. & Dimitrova, N. A. Simulation analysis of the ability to estimate motor unit propagation velocity non-invasively by different two-channel methods and types of multi-electrodes. *J Electromyogr Kinesiol* **13**, 403-415 (2003). https://doi.org:10.1016/s1050-6411(03)00036-1

9 Samii, A., Wassermann, E. M. & Hallett, M. Post-exercise depression of motor evoked potentials as a function of exercise duration. *Electroencephalogr Clin Neurophysiol* **105**, 352-356 (1997). https://doi.org:10.1016/s0924-980x(97)00033-7

10 Samii, A. *et al.* Decreased postexercise facilitation of motor evoked potentials in patients with chronic fatigue syndrome or depression. *Neurology* **47**, 1410-1414 (1996). https://doi.org:10.1212/wnl.47.6.1410

11 Hansen, J. E., Sue, D. Y. & Wasserman, K. Predicted values for clinical exercise testing. *Am Rev Respir Dis* **129**, S49-55 (1984). https://doi.org:10.1164/arrd.1984.129.2P2.S49

12 Wasserman K, S. W., Sun XG, Sue DY, Hansen JE, Whipp BJ, Sietsma KE. *Principals of Exercise Testing and Interpretation*. 5th edn, (Lippincott, Williams & Wilkins, 2011).

13 Pontzer, H. *et al.* Daily energy expenditure through the human life course. *Science* **373**, 808-812 (2021). https://doi.org:10.1126/science.abe5017

14 van den Berge, M. & Sijen, T. A male and female RNA marker to infer sex in forensic analysis. *Forensic Sci Int Genet* **26**, 70-76 (2017). https://doi.org:10.1016/j.fsigen.2016.10.018

15 Nkiliza, A. *et al.* Sex-specific plasma lipid profiles of ME/CFS patients and their association with pain, fatigue, and cognitive symptoms. *J Transl Med* **19**, 370 (2021). https://doi.org:10.1186/s12967-021-03035-6
