# Supplementary material for: Deep phenotyping of post-infectious myalgic encephalomyelitis/chronic fatigue syndrome
Source: Nat Commun. 2024 Feb 21;15:907. doi: 10.1038/s41467-024-45107-3 (PMC10881493; doi:10.1038/s41467-024-45107-3)
Supplement: Supplementary file 3 — Description of Additional Supplementary Files [file 41467_2024_45107_MOESM3_ESM.pdf]

Description of Additional Supplementary Files

File Name: Supplementary Data 1 - 24
Description:

Supplementary Data 1: Inclusion and Exclusion Criteria. Listing of the inclusion and exclusion criteria for PI-ME/CFS and HV participants as listed in the study protocol.

Supplementary Data 2: Summary of PI-ME/CFS participant screening and recruitment exclusions. Listing of the number of study inquiries and the number of excluded participants and the reasons for exclusion. 2A: A total of 484 PI-ME/CFS participant inquires were received. Of these, 267 were excluded at the time of initial screening. The number of persons excluded and the reasons are listed. Data 2B: A total of 217 participant inquires underwent medical record review. Of these, 146 were excluded after review. The number of persons excluded and the reasons are listed. Data 2C: A total of 27 participants underwent an in-person evaluation. Of these, 10 were excluded on the completion of the evaluation. The number of persons excluded and the reasons are listed. Data 2D: A total of 17 PI-ME/CFS participants were included in the study. All participants were evaluated according to the 2015 Institute of Medicine, 1994 Fukuda, and 2003 Canadian Consensus Criteria for ME/CFS. The number of participants meeting each criteria and reasons why participants failed to meet a criteria are listed.

Supplementary Data 3: List of research procedures. Listing of the individual research procedures performed in the study on all participants with additional brief details.

Supplementary Data 4: Statistical methods. Listing of the statistical methods used for analysis of each of the research procedures performed in the study. Further details regarding statistics can be found in the Methods section.

Supplementary Data 5: Participant demographics. Listing of the demographic characteristics of the HV (n=21) and PI-ME/CFS (n=17) groups. P-values calculated using Mann Whitney U tests are provided for select variables.

Supplementary Data 6: Validity testing Description. Listing of the group performance on neuropsychological validity tests for HV and PI-ME/CFS participants. The number of participants in each group is listed for each test. P-values were calculated with unadjusted two-sided t-test for independent samples with equal variance. An * denotes where equality of variance could not be assumed due to a significant Levene's test. WMT: Word Memory Test.

Supplementary Data 7: Patient reported outcome measures. Listing of the Patient reported outcome measures collected in the study. The mean and standard deviations are reported for the HV (n=21) and PI-ME/CFS (n=17) groups. P-values were calculated with unadjusted two sided Mann Whitney U tests. * denotes where HV n = 18. ** denotes where HV n = 20. *** denotes where PIME/CFS n = 16.

Supplementary Data 8: Clinical blood cell laboratory evaluation. Listing of all the clinical blood cell laboratory results collected in the study. The mean and standard deviations are reported for the HV (n=21) and PI-ME/CFS (n=17) groups. P-values were calculated with unadjusted two sided Mann Whitney U tests. * denotes the exclusion of one HV due to a RBC count of 3985, WBC 2. CBC: Complete blood count. WBC: White blood cells. RBC: Red blood cells. HGB: Hemoglobin. HCT: Hematocrit. MCV: Mean corpuscular volume. MCH: Mean corpuscular hemoglobin. MCHC: Mean corpuscular hemoglobin concentration. RDW: Red cell distribution width. MPV: Mean platelet volume. CSF: Cerebrospinal fluid. NK: Natural killer.

Supplementary Data 9: Clinical laboratory evaluation and screening measurements. Listing of all the clinical laboratory results collected in the study. The mean and standard deviations are reported for the HV (n=21) and PI-ME/CFS (n=17) groups. P-values were calculated with unadjusted two-sided Mann Whitney U tests. CO2: Carbon dioxide. BUN: Blood urea nitrogen.

Supplementary Data 10: Mitochondrial genetics in PI-ME/CFS. Listing of the variants in the mitochondrial genome were annotated to the 162 mitochondrial genes from PI-ME/CFS (n= 12) participants collected from muscle cells. The identity of each variant is noted by its gene name, position on the human chromosome or mitochondrial plasmid and c-dot number. Information about the variant's impact is listed in regards to peptide production, the type of variant, total percentage of the variant measured, variant class, zygosity, refGene functional classification, refGene exonic function, and refGene amino acid change description. Combined Annotation Dependent Depletion (CADD) scores and CADD Phred scores have been calculated for each variant.

Supplementary Data 11: Dietary Evaluation. Listing of the nutrient measures estimated in the study. The mean and standard deviations are reported for the HV (n=10) and PI-ME/CFS (n=11) who completed food records and the HV (n=15) and PI-ME/CFS (n=17) who completed the Diet History Questionnaire II. P-values were calculated with unadjusted two-sided t-test for independent samples with equal variance.

Supplementary Data 12: Total body energy use as measured before and after cardiopulmonary exercise testing (CPET). Listing of the energy expenditure, respiratory quotient, and movement measured during indirect calorimetry performed before, 3-19 hours, 27-43 hours, and 51-67 hours after a cardiopulmonary exercise test (CPET). The number of participants and the number of females is reported for each measurement. The mean and standard deviations are reported. For baseline total energy expenditure and sleeping energy expenditure, P-values were calculated with two-sided t-test for independent samples with equal variance adjusted for age, sex, ln(fat mass), and ln(fat free mass). For total energy expenditure and sleeping energy expenditure after CPET, P-values were calculated with two-sided t-test for independent samples with equal variance adjusted for age, sex, ln(fat mass), ln(fat free mass), and baseline energy expenditure value. For movement measures, P-values were calculated with unadjusted two-sided t-test for independent samples with equal variance.

Supplementary Data 13: Neuropsychological testing. Listing of neuropsychological tests performed in the study. The mean and standard deviations are reported for the HV (n=18) and PI-ME/CFS (n=16) groups. Pvalues were calculated with unadjusted two-sided t-test for independent samples with equal variance. An * denotes where equality of variance could not be assumed due to a significant Levene's test. The normative values for each instrument are listed as (M=X, SD=Y) where relevant. WTAR: Wechsler Test of Adult Reading. HVLT-R: Hopkins Verbal Learning Test-Revised. WAIS-IV: Wechsler Adult Intelligence Scale – Fourth Edition. BVMT-R: Brief Visual Memory Test-Revised. COWA: Controlled Oral Word Association Test. PASAT: Paced Auditory Serial Addition Test. TOVA: Test of Variables of Attention

Supplementary Data 14: Metabolomics of cerebrospinal fluid. Listing of the results obtained from cerebrospinal fluid metabolomics. 14A: Listing of analysis of group differences of individual metabolites collected from cerebrospinal fluid for HV (n = 21) and PI-ME/CFS (n=17) participants). The identity of each metabolite is noted by its CHEM ID number, Human Metabolome Database (HMDB) number, KEGG Pathway database number, the chemical name of the metabolite, and the related sub pathway and super pathway. P-values and False Discovery Rate corrected P-values were calculated with two-sided Wilcoxon tests for independent samples with equal variance. 14B: Variable importance in prediction (VIP) scores of indicated metabolites for HV (n = 21) and PI-ME/CFS (n=17) participants from cerebrospinal fluid samples. VIP scores were calculated using partial least squares discrimination analysis (PLSDA). 14C: Variable importance in prediction (VIP) scores of indicated metabolites in the male cohort for HV (n = 10) and PI-ME/CFS (n=7) participants from cerebrospinal samples. VIP scores were calculated using partial least squares discrimination analysis (PLSDA). 14D: Variable importance in prediction (VIP) scores of metabolites in the female cohort for HV (n = 11) and PI-ME/CFS (n=10) participants from cerebrospinal fluid samples. VIP scores were calculated using partial least squares discrimination analysis (PLSDA).

Supplementary Data 15: Flow cytometry analysis of the blood and cerebrospinal fluid samples. Listing of the results obtained from flow cytometry of blood and cerebrospinal fluid. 15A: Listing of individual flow cytometry values measured in blood for HV (n= 19) and PI-ME/CFS (n=15) participants. Results are listed in terms of percentages and ratios as indicated. P-values were calculated with unadjusted two-sided t-test for independent samples with equal variance. 15B: Listing of individual flow cytometry values measured in blood for HV (n= 7) and PI-ME/CFS (n=10) participants. Results are listed in terms of percentages and ratios as indicated. P-values were calculated with unadjusted two-sided t-test for independent samples with equal variance. These measurements were collected on independent samples from those used in 15A. 15C: Listing of individual flow cytometry values measured in cerebrospinal fluid for HV (n= 19) and PI-ME/CFS (n=16) participants. Results are listed in terms of percentages and ratios as indicated. P-values were calculated with unadjusted two-sided t-test for independent samples with equal variance. 15D: Listing of individual flow cytometry values measured in blood for HV (n= 7) and PI-ME/CFS (n=9) participants. Results are listed in terms of percentages and ratios as indicated. P-values were calculated with unadjusted two-sided t-test for independent

samples with equal variance. These measurements were collected on independent samples from those used in 15C.

Supplementary Data 16: Differential expression analysis of peripheral blood mononuclear cells. Listing of the results obtained from RNA sequencing of peripheral blood mononuclear cells. 16A: Listing of analysis of group differences of individual expressed genes collected from peripheral blood mononuclear cells for HV (n = 15) and PI-ME/CFS (n=14) participants. The identity of each expressed gene is noted by its Ensembl gene number and gene name. P-values and False Discovery Rate corrected P-values were calculated with two-sided Wilcoxon tests for independent samples with equal variance. 16B: Listing of analysis of group differences in the male cohort of individual expressed genes collected from peripheral blood mononuclear cells for HV (n = 7) and PI-ME/CFS (n=6 participants). The identity of each expressed gene is noted by its Ensembl gene number and gene name. P-values and False Discovery Rate corrected P-values were calculated with two-sided Wilcoxon tests for independent samples with equal variance. 16C: Listing of analysis of group differences in the female cohort of individual expressed genes collected from peripheral blood mononuclear cells for HV (n = 8) and PI-ME/CFS (n=8 participants). The identity of each expressed gene is noted by its Ensembl gene number and gene name. P-values and False Discovery Rate corrected P-values were calculated with two-sided Wilcoxon tests for independent samples with equal variance.

Supplementary Data 17: Individual data, univariate and multivariate analysis of aptamers measured on the SomaLogic platform in the serum and cerebrospinal fluid. Listing of the results obtained from Somalogic assay of serum and cerebrospinal fluid. 17A: Listing of individual Somalogic aptamers for HV (n = 18) and PIME/CFS (n=15) participants from serum samples measured in Relative Fluorescence Units (RFU). The identity of each aptamer is noted by seqID number, SomaID number, the common abbreviation of the aptamer (Target), and the full name of the aptamer (Target). P-values and False Discovery Rate corrected P-values were calculated with two-sided Wilcoxon tests for independent samples with equal variance. 17B: Listing of individual Somalogic aptamers for HV (n = 18) and PI-ME/CFS (n=15) participants from cerebrospinal fluid samples measured in Relative Fluorescence Units (RFU). The identity of each aptamer is noted by seqID number, SomaID number, the common abbreviation of the aptamer (Target), and the full name of the aptamer (Target Name). P-values and False Discovery Rate corrected P-values were calculated with twosided Wilcoxon tests for independent samples with equal variance. 17C: Variable importance in prediction (VIP) scores of indicated soma protein aptamers in the male cohort for HV (n = 8) and PI-ME/CFS (n=5) participants from serum samples. VIP scores were calculated using partial least squares discrimination analysis (PLSDA). 17D: Variable importance in prediction (VIP) scores of indicated soma protein aptamers in the male cohort for HV (n = 8) and PI-ME/CFS (n=5) participants from cerebrospinal fluid samples. VIP scores were calculated using partial least squares discrimination analysis (PLSDA). Matching UniProt and EntrezGeneID Name and ID are also provided. 17E: Variable importance in prediction (VIP) scores of indicated soma protein aptamers in the female cohort for HV (n = 10) and PI-ME/CFS (n=10) participants from serum samples. VIP scores were calculated using partial least squares discrimination analysis (PLSDA). Matching UniProt and EntrezGeneID Name and ID

are also provided. 17F: Variable importance in prediction (VIP) scores of indicated soma protein aptamers in the female cohort for HV (n = 10) and PI-ME/CFS (n=10) participants from cerebrospinal fluid samples. VIP scores were calculated using partial least squares discrimination analysis (PLSDA). Matching UniProt and EntrezGeneID Name and ID are also provided.

Supplementary Data 18: Transposable element differential expression analysis. Listing of analysis of group differences in transposable elements of individual expressed genes collected from peripheral blood mononuclear cells for HV (n = 15) and PI-ME/CFS (n=14) participants. The identity of each transposable element is noted by gene ID. P-values and False Discovery Rate corrected P-values were calculated with the Wald test.

Supplementary Data 19: Differential expression (DE) analysis muscle. Listing of the results obtained from RNA sequencing of muscle.19A: Listing of analysis of group differences of individual expressed genes collected from muscle cells for HV (n = 12) and PI-ME/CFS (n=13) participants). The identity of each expressed gene is noted by its Ensembl gene number and gene name. P-values and False Discovery Rate corrected P-values were calculated with two-sided Wilcoxon tests for independent samples with equal variance. 19B: Listing of analysis of group differences in the male cohort of individual expressed genes collected from muscle cells for HV (n = 7) and PI-ME/CFS (n=6 participants). The identity of each expressed gene is noted by its Ensembl gene number and gene name. P-values and False Discovery Rate corrected Pvalues were calculated with two-sided Wilcoxon tests for independent samples with equal variance. 19C: Listing of analysis of group differences in the female cohort of individual expressed genes collected from muscle cells for HV (n = 5) and PI-ME/CFS (n=7 participants). The identity of each expressed gene is noted by its Ensembl gene number and gene name. P-values and False Discovery Rate corrected P-values were calculated with two-sided Wilcoxon tests for independent samples with equal variance.

Supplementary Data 20: Individual data and univariate lipidomics analysis. Listing of individual lipid composition measured in plasma for HV (n = 18) and PI-ME/CFS (n=15) participants from serum samples measured in in µM. P-values values were calculated with two-sided Wilcoxon tests for independent samples with equal variance. DAG: diacylglycerol. TAG: triacylglycerol. FFA: free fatty acid. CER: ceramide. HCER: hexosylceramide. LPE: lysophosphatidylethanolamine. DCER: dihydroceramide. CE: cholesteryl ester. SM: sphingomyelin. LCER: lactosylceramide. PE: phosphatidylethanolamine. LPC: lysophosphatidylcholine. PC: phosphatidylcholine.

Supplementary Data 21: Individual data and univariate analysis of stool metabolomics. Listing of individual metabolites for HV (n = 17) and PI-ME/CFS (n=16) participants from stool samples measured in µM. P-values values were calculated with two-sided Wilcoxon tests for independent samples with equal variance.

Supplementary Data 22: Negative findings. Listing of the negative findings in the study and how they were measured.

Supplementary Data 23: Data exclusions. Listing of all of the types of data, the number of data points excluded, and the reasons why each data point was excluded.

Supplementary Data 24: Abbreviations. Listing of all of the abbreviations used within the main manuscript and supplementary information.
