# Supplementary material for: Deep phenotyping of post-infectious myalgic encephalomyelitis/chronic fatigue syndrome
Source: Nat Commun. 2024 Feb 21;15:907. doi: 10.1038/s41467-024-45107-3 (PMC10881493; doi:10.1038/s41467-024-45107-3)
Supplement: Supplementary file 5 — Reporting Summary [file 41467_2024_45107_MOESM5_ESM.docx]

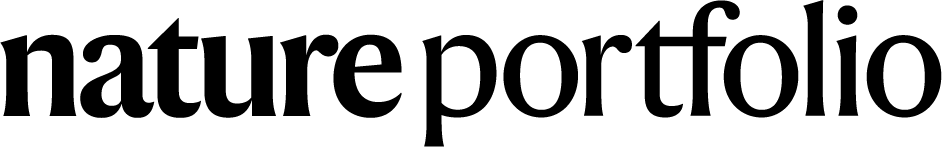
Corresponding author(s): Avindra Nath

Last updated by author(s): 2024/1/04

Reporting Summary

Nature Portfolio wishes to improve the reproducibility of the work that we publish. This form provides structure for consistency and transparency in reporting. For further information on Nature Portfolio policies, see our Editorial Policies and the Editorial Policy Checklist.

Please do not complete any field with "not applicable" or n/a. Refer to the help text for what text to use if an item is not relevant to your study. For final submission: please carefully check your responses for accuracy; you will not be able to make changes later.

## Statistics

For all statistical analyses, confirm that the following items are present in the figure legend, table legend, main text, or Methods section.

n/a


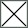


Confirmed


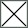
 The exact sample size (*n*) for each experimental group/condition, given as a discrete number and unit of measurement


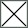
 A statement on whether measurements were taken from distinct samples or whether the same sample was measured repeatedly The statistical test(s) used AND whether they are one- or two-sided

*Only common tests should be described solely by name; describe more complex techniques in the Methods section.*


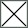
 A description of all covariates tested


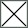
 A description of any assumptions or corrections, such as tests of normality and adjustment for multiple comparisons

A full description of the statistical parameters including central tendency (e.g. means) or other basic estimates (e.g. regression coefficient) AND variation (e.g. standard deviation) or associated estimates of uncertainty (e.g. confidence intervals)

For null hypothesis testing, the test statistic (e.g. *F*, *t*, *r*) with confidence intervals, effect sizes, degrees of freedom and *P* value noted

1

nature portfolio | reporting summary

*April 2023*

*Give P values as exact values whenever suitable.*

For Bayesian analysis, information on the choice of priors and Markov chain Monte Carlo settings

For hierarchical and complex designs, identification of the appropriate level for tests and full reporting of outcomes Estimates of effect sizes (e.g. Cohen's *d*, Pearson's *r*), indicating how they were calculated

*Our web collection on statistics for biologists contains articles on many of the points above.*

## Software and code

Policy information about availability of computer code Data collection

Recruitment: Microsoft Excel version 2302

Demographics: NICHD Clinical Trials Database system (CTDB) Qualitative Interviews: Voice recorder

Validity Testing: Pen and Paper tests

Physical Symptoms: NICHD Clinical Trials Database system (CTDB) Clinical Evaluation: NIH Clinical Research Information System (CRIS) Neurological Exam: NIH Clinical Research Information System (CRIS) Brain MRI: 3.0 tesla Philips Achieva

Neuron injury markers: Simoa HD-X Automated Immunoassay Analyzer Small nerve fiber analysis: Confocal microscopy

Clinical labs: NIH Clinical Research Information System (CRIS) Iron Plasma Spectroscopy ICP-MS: Agilent Model 7900

Psychological Outcomes: NICHD Clinical Trials Database system (CTDB) SCID-V: NICHD Clinical Trials Database system (CTDB)

Medications: NIH Clinical Research Information System (CRIS)

Body Composition: Scale-Tronix 5702 digital balance, Seca 242 stadiometer, iDXA scanner with Encore 15.0 software Mitochondrial Genetics: Gene DX proprietary NGS-CNV

Muscle fiber analysis: Fiji ImageJ

DHQII: On-line data collection (Diet History Questionnaire II (DHQ II) for U.S. and Canada | EGRP/DCCPS/NCI/NIH (cancer.gov)) Diet interview: Pen and paper interview

Heart rate variability: Spacelabs Evo 4 ECG Recorder with 4-lead Cables

2

nature portfolio | reporting summary

*April 2023*

Orthostatic Challenge: LabChart Pro v. 7

Baroreflex function: LabChart Pro v. 7

Effort Measures: EEfRT test (v 8/2014): Tools — TReAD Lab Actigraphy: ActiGraph GT3X+ accelerometers

Grip Strength: Jamar hand-held dynamometer, NICHD Clinical Trials Database system (CTDB)

Repetitive Grip – TMS: Biopac rigid-frame dynamometer, 3m surface electrodes, Cambridge Electronic Design amplifiers and software, Rogue Research Neuronavigation system,

Repetitive Grip – fMRI: 3T Prisma SIEMENS scanner equipped with a 64-channel head-coil

Cardiopulmonary Exercise Test: CardiO2 Ultima computerized metabolic cart ; NIRO-200NX infrared spectrometer

Chamber - respiratory exchange: NIH Whole room indirect calorimeter using meters for CO2 (ABB AO2000) and O2 (Siemens Oxymat 6E) concentration recorded each minute using CalRQ (MEI Research)

Mitochondrial Flux Assay: Seahorse XF mini analyzer Cortisol: Proprietary Salimetrics ELIZA

Neuropsychological testing: Paper and pen tests; computerized tests

CSF and blood catechols: Waters Model 710B (WISP) chromatography system; ESA Model 590 Coulochem electrochemical detector and triple electrode system (Models 5021 and 5011)

CSF metabolomics: Proprietary Metabolon Ultrahigh Performance Liquid Chromatography-Tandem Mass Spectroscopy Flow cytometry: Becton Dickenson LSR II

RNA Seq – PBMCS: Qubit 3.0 fluorometer, Agilent 2100 Bioanalyzer, HiSeq 3000 sequencer Somalogic: SOMAscan 1.3k Assay

External Validations: NA

NK cell function, Cincinnati Children's Hospital: 51-Chromium NK cell assay GDF-15: R&D Systems ELIZA

LIPS: Berthold LB 960 Centro luminometer

Endogenous retroviral: Qubit 3.0 fluorometer, Agilent 2100 Bioanalyzer, HiSeq 3000 sequencer RNA Seq – muscle: DeNovix DS-11 spectrophotometer, Illumina NovaSeq 6000 sequencer

Lipidomics: Selexion on the 5500 QTRAP for the Lipidyzer platform, with Analyst 1.6.3 and the Lipidomics Workflow Manager 1.0.5.0 Microbiome metagenomics: NoneIllumina Nextera Flex kit, and then sequenced on the Illumina NovaSeq platform with a 2 x 150 bp length configuration

Stool nMRS: VNMR (Agilent, Inc.)

Data analysis

Codes for the bioinformatic analysis of shotgun metagenomics are available at: https://github.com/johnmcculloch/JAMS_BW. The JAMS compatible Kraken2 taxonomic classification database used for shotgun metagenomics is available at: https://hpc.nih.gov/~mccullochja/ JAMSdb202201.tar.gz. RNA sequence quality control was performed with FastQC: [http://www.bioinformatics.babraham.ac.uk/projects/fastqc,](http://www.bioinformatics.babraham.ac.uk/projects/fastqc) trimmomatic: https://github.com/timflutre/trimmomatic, and multiqc: https://multiqc.info/. Codes for differential gene expression analysis are available at: COVID-19_Transcriptomics/Differential_gene_expression.R at master · NHLBI-BCB/COVID-19_Transcriptomics · GitHub. Codes for Pathway enrichment analysis are available at: COVID-19_Transcriptomics/PathwayEnrichment_clusterProfiler.R at master · NHLBI-BCB/ COVID-19_Transcriptomics · GitHub. Transposable Seqeunce analysis performed with STAR: https://github.com/alexdobin/STAR, samtools: https://github.com/samtools/samtools, and TEcount tool: (https://github.com/mhammell-laboratory/TEtranscripts.

Additionally, all the above codes and additional R scripts used in this study are available at: https://github.com/docwalitt/National-Institutes- of-Health-Myalgic-Encephalomyelitis-Chronic-Fatigue-Syndrome-Code-Repository.

For manuscripts utilizing custom algorithms or software that are central to the research but not yet described in published literature, software must be made available to editors and reviewers. We strongly encourage code deposition in a community repository (e.g. GitHub). See the Nature Portfolio guidelines for submitting code & software for further information.

## Data

Policy information about availability of data

All manuscripts must include a data availability statement. This statement should provide the following information, where applicable:

- Accession codes, unique identifiers, or web links for publicly available datasets
- A description of any restrictions on data availability
- For clinical datasets or third party data, please ensure that the statement adheres to our policy

The Map ME/CFS databank (accession code: [https://www.mapmecfs.org/group/post-infectious-mecfs-at-the-nih)](http://www.mapmecfs.org/group/post-infectious-mecfs-at-the-nih)) contains demographics, performance validity testing, patient reported outcomes, small nerve fiber measures, neuronal injury markers, clinical lab data, heart rate variability measures, orthostatic challenge data, psychological scales, body composition measures, extracellular flux measures of PBMCs, muscle fiber measures, actigraphy and strength measures, cardiopulmonary exercise test data, whole room calorimetry, neuropsychological testing, biological measures of blood and cerebrospinal fluid, 51 Chromium release assay, metabolomics, proteomics, lipidomics, mitochondrial sequencing, and stool nuclear magnetic resonance spectroscopy data. Accessing Map ME/CFS data requires signing up for an account but otherwise access to the data is unrestricted (Creative Commons BY 4.0). PBMC gene expression data (GEO Accession viewer (nih.gov): Accession Code GSE251872), muscle gene expression data ( GEO Accession viewer (nih.gov): Accession Code GSE245661), and proteomics data (GSE251790 - GEO DataSets - NCBI (nih.gov): Accession Code: GSE251790; GSE251792 - GEO DataSets - NCBI (nih.gov): Accession Code: GSE251792 ) is available at Gene Expression Omnibus (GEO) and stool shotgun metagenomic data (SRA Links for BioProject (Select 954397) - SRA - NCBI (nih.gov), Accession Code SRP467038) are available at Sequence Read Archive, which are all available at BioProject (Homo sapiens (ID 954397) - BioProject - NCBI (nih.gov): Accession Code PRJNA954397). All neurophysiology data from transcranial magnetic stimulation and functional magnetic resonance imaging experiments are available at Pennsieve (Deep phenotyping of Post-infectious Myalgic Encephalomyelitis-Chronic Fatigue Syndrome - Blackfynn Discover (pennsieve.io); DOI: 10.26275/ile7-wrsk). External datasets analyzed include GEO GSE13033 (GEO Accession viewer (nih.gov)) and GEO GSE156792 (GEO Accession viewer (nih.gov)). Source data are provided with this paper.

3

nature portfolio | reporting summary

*April 2023*

## Research involving human participants, their data, or biological material

Policy information about studies with human participants or human data. See also policy information about sex, gender (identity/presentation), and sexual orientation and race, ethnicity and racism.

Reporting on sex and gender

Birth sex analysis was performed throughout our analysis and differences based on birth sex are a central part of our manuscript. As there were no participants with differing gender from birth sex, gender based analysis were not possible. Corresponding birth sex and gender information is available for all data listed above in the data availability section, which can be used to perform further stratified analyses.

Reporting on race, ethnicity, or other socially relevant groupings

Race and ethnicity demographic information was collected on all participants using self-report collection during the intake interview. The sample sizes in this study are too small to perform sub-analyses of data based on race or ethnicity.

Corresponding race and ethnicity information is available for all data listed above in the data availability section, which can be used to perform further stratified analyses.

Population characteristics

Population characteristics for each participant are provided in Table S5. These include age at evaluation, birth sex, gender, race, ethnicity, marital status, education, disability status, and body mass index.

Recruitment

The PI-ME/CFS group was selected based on medical record documentation of persistent and severe fatigue and post- exertional malaise as the consequence of an acute infection within the last five years without a prior history of explanatory medical or psychiatric illness. The recruitment process entailed a series of filtering reviews, including a telephone screen, medical record review, and physician telephone interview to determine eligibility. Of 484 inquiries and 217 medical reviews, only 27 participants met criteria for research evaluation. Of those, only 17 were found to meet all eligibility requirements for study inclusion. The stringent inclusion and exclusion criteria served to mitigate the self-referral bias inherent in the study recruitment.

Ethics oversight

National Institutes of Health Institutional Review Board (NCT 02669212)

Note that full information on the approval of the study protocol must also be provided in the manuscript.

# Field-specific reporting

Please select the one below that is the best fit for your research. If you are not sure, read the appropriate sections before making your selection.


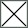
 Life sciences
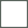
 Behavioural & social sciences
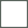
 Ecological, evolutionary & environmental sciences

For a reference copy of the document with all sections, see nature.com/documents/nr-reporting-summary-flat.pdf

# Life sciences study design

All studies must disclose on these points even when the disclosure is negative. Sample size

The current sample size of 17 PI-ME/CFS and 21 healthy volunteers can achieve a power of 80% with an effect size of 0.94.

Data exclusions

Recruitment: No excluded data Demographics: No excluded data Qualitative Interviews: No excluded data Validity Testing : No excluded data Physical Symptoms: No excluded data Clinical Evaluation: No excluded data Neurological Exam: No excluded data Brain MRI: No excluded data

Neuron injury markers: 16 of 39 of the plasma measures of UCHL1 had Coefficient of Variance scores ≥ 0.2, suggesting a technical issue with measurement. For this reason, the results of the UCHL1 analysis were excluded from the manuscript.

Small nerve fiber analysis: 9 of 48 skin samples did not contain a sweat gland needed for measuring sweat gland nerve fiber density. These nine samples were not included in the analysis.

Clinical labs: No excluded data

Iron Plasma Spectroscopy: No excluded data Psychological Outcomes : No excluded data SCID-V: No excluded data

Medications: No excluded data

Body Composition: No excluded data Mitochondrial Genetics: No excluded data

Muscle fiber analysis: 1 set of samples were excluded due to poor quality of the tissue section across the entire area. DHQII: No excluded data

Diet interview: No excluded data Polysomnography: No excluded data

Heart rate variability: 1 HV excluded due to file corruption during data import; 2 PI-ME/CFS excluded due to beta blocker use. Orthostatic Challenge: No excluded data

Baroreflex function: No excluded data

Effort Measures: 1 participant excluded due to invalid data.

4

nature portfolio | reporting summary

*April 2023*

Actigraphy: No excluded data

Grip Strength: No excluded data Repetitive Grip – TMS: No excluded data

Repetitive Grip – fMRI: 1 HV excluded due to rhythmic breathing yielding lots of head movement impacting the quality of 80% of fMRI data. Cardiopulmonary exercise test: No excluded data

Chamber - respiratory exchange : No excluded data Mitochondrial Flux Assay: No excluded data Cortisol: No excluded data

Neuropsychological testing: No excluded data CSF catechols: No excluded data

CSF metabolomics: No excluded data Flow cytometry: No excluded data RNA Seq – PBMCs: No excluded data Somalogic: No excluded data

External Validations: No excluded data NK cell: No excluded data

GDF-15: No excluded data LIPS: No excluded data

Endogenous retroviral: No excluded data

RNA Seq – muscle: 1 PI-ME/CFS participant excluded due to extreme outlying data compared to both the PI-ME/CFS and HV groups. Lipidomics: No excluded data

Microbiome metagenomics: No excluded data Stool nMRS: No excluded data

Replication

Two or more technical replicates were used for all biological samples analyzed. Clinical evaluations were performed without replicates.

Randomization

There was no randomization in this study. There were no interventions used in this study.

Blinding

All researchers performing the experimental analysis of biological samples were blinded to diagnostic group. Evaluating clinicians were not blinded to diagnostic group.

# Reporting for specific materials, systems and methods

We require information from authors about some types of materials, experimental systems and methods used in many studies. Here, indicate whether each material, system or method listed is relevant to your study. If you are not sure if a list item applies to your research, read the appropriate section before selecting a response.

Materials & experimental systems Methods


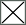

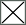

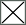

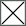

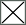


n/a Involved in the study Antibodies Eukaryotic cell lines

Palaeontology and archaeology Animals and other organisms Clinical data

Dual use research of concern

Plants


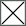

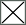


n/a Involved in the study

ChIP-seq

Flow cytometry

MRI-based neuroimaging

## Antibodies

Antibodies used

All antibodies, clones, catalog numbers, manufacturers, and dilutions used in this study are as follow. Dilutions were determined according to our own staining protocols.

anti-human CD3 (clone: UCHT1, Cat. 558117, BD Biosciences, 1:30 dilution (1:100 dilution for CSF cells)) anti-human CD3 (clone: UCHT1, Cat. 555335, BD Biosciences, 1:100 dilution)

anti-human CD4 (clone: RPA-T4, Cat. 557922, BD Biosciences, 1:30 dilution (1:100 dilution for CSF cells)) anti-human CD4 (clone: RPA-T4, Cat. 555346, BD Biosciences, 1:100 dilution)

anti-human CD8 (clone: SK1, Cat. 341051, BD Biosciences, 1:30 dilution (1:100 dilution for CSF cells)) anti-human CD8 (clone: RPA-T8, Cat. 557746, BD Biosciences, 1:100 dilution)

anti-human CD14 (clone: M5E2, Cat. 555399, BD Biosciences, 1:30 dilution (1:100 dilution for CSF cells)) anti-human CD16 (clone: 3G8, Cat. 338426, BD Biosciences, 1:30 dilution (1:100 dilution for CSF cells)) anti-human CD19 (clone: HIB19, Cat. 555413, BD Biosciences, 1:30 dilution (1:100 dilution for CSF cells)) anti-human CD25 (clone: M-A251, Cat. 557741, BD Biosciences, 1:30 dilution (1:100 dilution for CSF cells)) anti-human CD27 (clone: M-T271, Cat. 560222, BD Biosciences, 1:30 dilution (1:100 dilution for CSF cells)) anti-human CD45 (clone: HI30, Cat. 560777, BD Biosciences, 1:30 dilution (1:100 dilution for CSF cells))

anti-human CD45RA (clone: HI100, Cat. 555488, BD Biosciences, 1:30 dilution (1:100 dilution for CSF cells)) anti-human CD56 (clone: B159, Cat. 557747, BD Biosciences, 1:30 dilution (1:100 dilution for CSF cells)) anti-human CD226 (clone: 11A8, Cat. 338318, BioLegend, 1:30 dilution (1:100 dilution for CSF cells))

anti-human CD244 (clone: C1.7, Cat. 329522, BioLegend, 1:30 dilution (1:100 dilution for CSF cells)) anti-human PD-1 (clone: EH12.2H7, Cat. 329906, BioLegend, 1:30 dilution (1:100 dilution for CSF cells))

5

nature portfolio | reporting summary

*April 2023*

anti-human CXCR5 (clone: RF8B2, Cat. 558113, BD Biosciences, 1:30 dilution (1:100 dilution for CSF cells))

anti-human HLA-DR (clone: G46-6, Cat. 555811, BD Biosciences, 1:30 dilution (1:100 dilution for CSF cells)) anti-human IgD (clone: IA6-2, Cat. 561302, BD Biosciences, 1:30 dilution (1:100 dilution for CSF cells))

anti-human TIGIT (clone: A15153G, Cat. 372714, BioLegend, 1:30 dilution (1:100 dilution for CSF cells)) anti-human CD127 (clone: HIL-7R-M21, Cat. 560551, BD Biosciences, 1:30 dilution)

anti-FOXP3 (clone: 236A/E7, Cat. 17-4777-42, Thermo Fisher Scientific, 1:50 dilution) anti-human CD152 (clone: BNI3, Cat. 555853, BD Biosciences, 1:50 dilution)

anti-Stat5 (pY694) (clone: 47/Stat5(pY694), Cat. 612567, BD Biosciences, 1:50 dilution)

Validation

All the antibodies used for flow cytometry in this study are commercially available, and their specificities have been well validated by the manufacturers and other users.

## Clinical data

Policy information about clinical studies

All manuscripts should comply with the ICMJE guidelines for publication of clinical research and a completed CONSORT checklist must be included with all submissions.

Clinical trial registration

NCT02669212

Study protocol

The most recent version of the research protocol (16-N-0058) is attached to the submission

Data collection

All data was collected at the National Institutes of Health Clinical Center or at the participant's place of residence. Data was collected between December 2016 and February 2020

Outcomes

This is a case-control study in which no intervention was done. Hence, there are no primary or secondary outcome measures.

## Plants

Seed stocks

*Report on the source of all seed stocks or other plant material used. If applicable, state the seed stock centre and catalogue number. If plant specimens were collected from the field, describe the collection location, date and sampling procedures.*

*Describe the methods by which all novel plant genotypes were produced. This includes those generated by transgenic approaches, gene editing, chemical/radiation-based mutagenesis and hybridization. For transgenic lines, describe the transformation method, the number of independent lines analyzed and the generation upon which experiments were performed. For gene-edited lines, describe the editor used, the endogenous sequence targeted for editing, the targeting guide RNA sequence (if applicable) and how the editor was applied.*

*Describe any authentication procedures for each seed stock used or novel genotype generated. Describe any experiments used to*

*assess the effect of a mutation and, where applicable, how potential secondary effects (e.g. second site T-DNA insertions, mosiacism, off-target gene editing) were examined.*

Novel plant genotypes

Authentication

## Magnetic resonance imaging

### Experimental design

Design type

Brain activity was also assessed during the grip strength task with fMRI. Volunteers lay supine in the scanner and performed repeated 30-second blocks of grip strength with a dynamometer (isometric muscle contractions) at 50% of their maximum voluntary contraction (MVC) interspaced with 30-second blocks of rest. MVC of the forearm muscles was determined from the best of three brief squeezes on the dynamometer. Volunteers used visual feedback from a computer to monitor force generation.

Design specifications

The number of grip blocks performed was dependent on how fast the participants tired. Participants were free to stop the task when they felt too tired to continue. Most participants performed 16 blocks of grip/rest, thus 16 minutes.

Behavioral performance measures

We measured force in Kilograms and calculated the number of performed blocks. The metric to ensure participants compliance is to track performance be above the 50% of MVC mark

### Acquisition

Functional

Imaging type(s)

Field strength

3 Tesla

Sequence & imaging parameters

T2*-weighted EPI with TR = 2 sec, TE = 30 ms, image matrix = 64 x 64, flip angle: 70, FoV: 100, voxel size 3.5 x 3.5 x 3.5 mm

Area of acquisition

Whole brain

Diffusion MRI
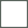
 Used
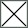
 Not used

### Preprocessing

Preprocessing software

For fMRI and resting-state we used AFNI (https://afni.nimh.nih.gov/; version AFNI_23.0.04) and Matlab [(www.mathworks.com/;](http://www.mathworks.com/%3B) version 9.10.0.1602886 (R2021a)) and Matlab for grip force analysis

Normalization

We used the Montreal Neurological Institute brain template to non-linearly warp (normalize) anatomical and functional data or the force task.

Normalization template

We used the Montreal Neurological Institute brain template: MN1152_2009_template_SSW.nii.gz

Noise and artifact removal

We used standard noise removal including spike removal, motion parameters are set to 0.3 mm. We also removed non-brain signal by regressing out cerebrospinal fluid and white-matter signals from the timeseries of grip force task.

Volume censoring

We used AFNI to censor volumes based on head motion (above 0.3 mm) and data quality (outliers as detected by 3dToutcount in AFNI) and volumes are censored if more than 10% of the voxels in a volume are outliers.

### Statistical modeling & inference

Model type and settings

We used mass univariate. We used a two-level analysis, a the subject-level and then a group-level. At subject level, we used a box-car block design analysis to estimate brain activation related to groups of 4 grip force blocks. At group-level, we used linear mixed-effects 2-way ANOVA with groups (healthy volunteers and PI-ME/CFS; fixed effect) and blocks (1,2,3,4; repeated measures) with participants as random factor.

Effect(s) tested

We compared brain activation between grip blocks and rest blocks with 2-way ANOVA with groups (healthy volunteers and

PI-ME/CFS; fixed effect) and blocks (1,2,3,4; repeated measures) with participants as random factor. We used the 3dLME tool in AFNI

Specify type of analysis:
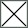
 Whole brain
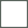
 ROI-based
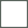
 Both Statistic type for inference

We used 2-stage voxels and cluster threshold with voxel threshold set p 0.01 and then corrected for multiple comparisons with a p 0.05, yielding a cluster threshold of > 65 voxels, as implemented in AFNI

(See Eklund et al. 2016)

Correction

We used family-wise error correction. As implemented in AFNI we use a voxel and then a cluster threshold to correct for multiple comparisons. We choose p 0.01 at voxel level and p 0.05 for cluster threshold.

### Models & analysis

n/a


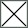

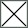

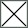


Involved in the study


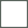
 Functional and/or effective connectivity
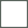
 Graph analysis

Multivariate modeling or predictive analysis

6

nature portfolio | reporting summary

*April 2023*


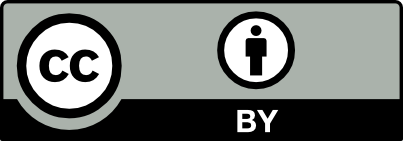
This checklist template is licensed under a Creative Commons Attribution 4.0 International License, which permits use, sharing, adaptation, distribution and reproduction in any medium or format, as long as you give appropriate credit to the original author(s) and the source, provide a link to the Creative Commons license, and indicate if changes were made. The images or other third party material in this article are included in the article's Creative Commons license, unless indicated otherwise in a credit line to the material. If material is not included in the article's Creative Commons license and your intended use is not permitted by statutory regulation or exceeds the permitted use, you will need to obtain permission directly from the copyright holder. To view a copy of this license, visit <http://creativecommons.org/licenses/by/4.0/>
